# Total Synthesis of the Dihydrooxepine-Spiroisoxazoline Natural Product Psammaplysin A

**DOI:** 10.1021/jacs.2c10010

**Published:** 2022-10-21

**Authors:** Jan Paciorek, Denis Höfler, Kevin Rafael Sokol, Klaus Wurst, Thomas Magauer

**Affiliations:** † Institute of Organic Chemistry and Center for Molecular Biosciences, Leopold-Franzens-University Innsbruck, Innrain 80-82, 6020 Innsbruck, Austria; § Institute of General, Inorganic & Theoretical Chemistry, Leopold-Franzens-University Innsbruck, Innrain 80-82, 6020 Innsbruck, Austria

## Abstract

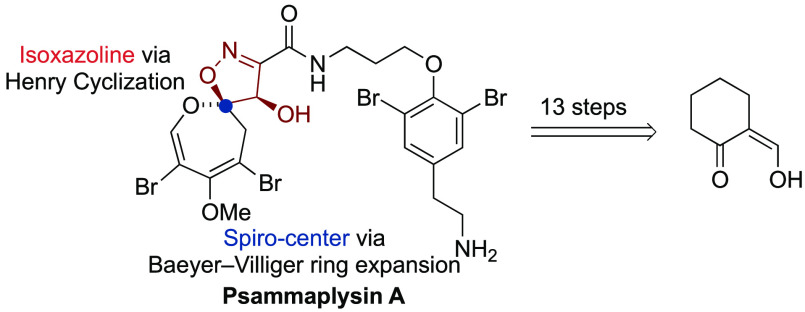

We report a general synthetic entry to dihydrooxepine-spiroisoxazoline (DOSI) natural products that culminated in the first racemic total synthesis of psammaplysin A. For the synthesis of the unique spirocyclic fragment we employed a strategy that features two key transformations: (1) a diastereoselective Henry reaction/cyclization sequence to access the C7 hydroxylated isoxazoline scaffold in one step and (2) a regioselective Baeyer–Villiger ring expansion to install the fully substituted dihydrooxepine and avoid the risk of a previously observed oxepine-arene oxide rearrangement. The overall synthesis proceeds in 13 steps from an inexpensive starting material.

Dihydrooxepine-spiroisoxazoline (DOSI) natural products are a structurally unique family of marine alkaloids that comprises the psammaplysins, ceratinamides, ceratinadins, and frondoplysins.^1^ Among them, the psammaplysins stand out as the largest class (>35 members), with many displaying anticancer, antimalarial, anti-HIV, or antibiotic activities.^2^ In 1982, psammaplysin A (**1**) was isolated as the first member from the marine sponge *Psammaplysilla purpurea* by Kashman (Scheme 1A).^3^ The structure of **1** was initially proposed to feature a C6 cyclohexadiene-spiroisoxazoline motif as present in aerothionin (**2**). While this assignment was revised in 1985 by Clardy based on detailed NMR studies and single-crystal X-ray crystallographic analysis,^4^ it took another 30 years to validate the proposed absolute configuration.^5^ The structurally unique dihydrooxepine motif is thought to be biosynthetically derived from the oxidation of dibromotyrosine (**3**) followed by ring opening.^4^ For the formation of aerothionin (**2**), a direct intramolecular opening of the putative epoxide intermediate at C6 was postulated and a 6π-electrocyclic ring-opening event that initiates the rearrangement of the arene oxide to the oxepine might be involved in the formation of **1** (compare with Scheme 1B).^6^ This divergent process was investigated in the seminal work by Clardy, which enabled the formal synthesis of **2**, but it did not lead to **1**.^7^ The *S*-adenosylmethionine (SAM, **4**) derived three-carbon amide linker of **1** connects the DOSI to a modified dibromotyrosine and distinguishes it from its natural congeners, some of which possess an additional C19 stereocenter.^6c,8^ The intriguing biological properties and unique functionalization pattern of the molecular framework render **1** a formidable synthetic target.

**Scheme 1:**
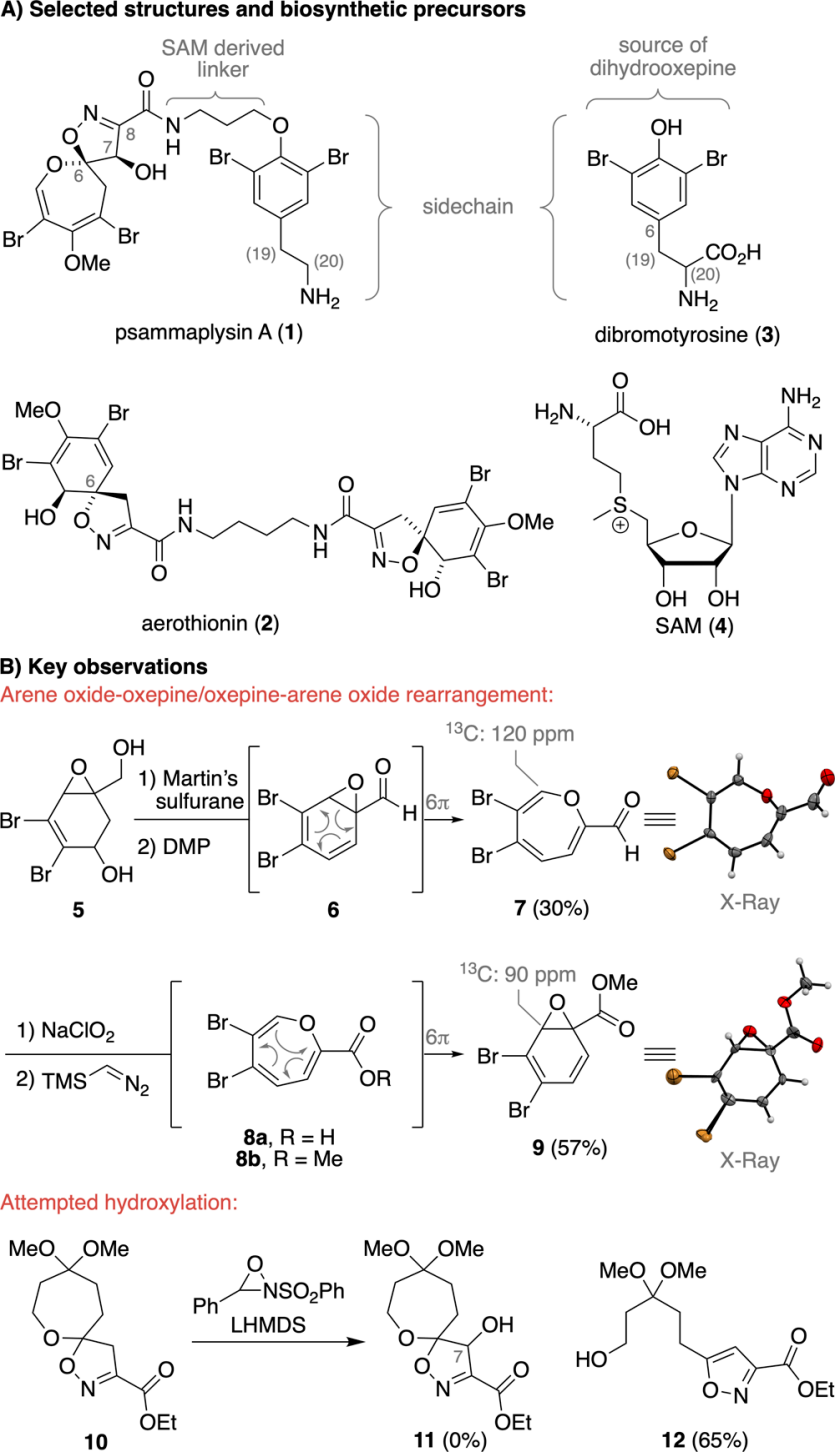
Dihydrooxepine-spiroisoxazoline (DOSI) Natural Products and Preliminary Insights

Surprisingly, despite the early studies by Clardy,^7^ an increasing number of powerful synthetic methods for construction of dihydrooxepines,^9^ and more recent work by Vanderwal and Yang,^10^ a synthesis of DOSI natural products has yet to be accomplished. As part of our ring expansion program,^11^ we set out to develop a general synthetic route to **1** that would permit access to the entire DOSI family.

During preliminary studies in our laboratories, we found that aldehyde **6**, obtained from the dehydration with Martin’s sulfurane and oxidation of diol **5** with Dess–Martin periodinane (DMP), undergoes spontaneous arene oxide-oxepine rearrangement^12^ to oxepine **7** in 30% yield (Scheme 1B). We initially considered oxepine **7** as a valuable precursor for **1**, but **7** proved to be unstable upon storage and underwent slow decomposition to unidentified aromatic byproducts. Unexpectedly, attempted conversion of oxepine **7** to ester **8b** was even more problematic as arene oxide **9** was obtained as the only product. A second key observation was made when we investigated the hydroxylation of isoxazoline **10**, the product of a preceding nitrile oxide [3 + 2]-cycloaddition reaction (see Supporting Information for details).^10b,13^ In this case, hydroxylation to isoxazoline **11** was outcompeted by rapid ring opening of the aza enolate to give isoxazole **12** as the sole product in 65% yield.^14,15^ These key insights were crucial for our further synthetic planning and ultimately led us to the development of the revised retrosynthesis outlined in Scheme 2.

**Scheme 2:**
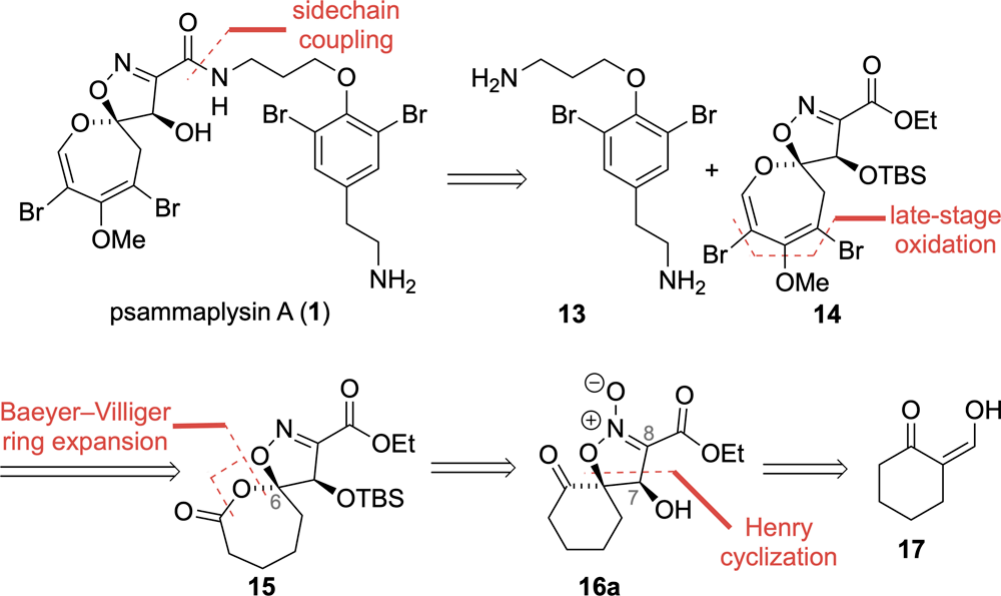
Retrosynthetic Strategy for Psammaplysin A

We began with the disconnection of the known linker side chain moloka’iamine (**13**)^16^ from **1** and focused our further analysis on the resulting DOSI **14**. We anticipated that the lack of a fully decorated oxepine should eliminate the risk of unwanted arene oxide formation. Sequential removal of the enol ether decoration of ester **14** revealed structurally simplified lactone **15**. The lactone acetal contains the retron for a Baeyer–Villiger oxidation of C6 producing spirocyclic ketone **16a**.^17,11a^ To avoid potential ring opening as observed for isoxazoline **10**, we considered simultaneous installation of the requisite C7 hydroxy group and the isoxazoline scaffold in one step employing a powerful diastereoselective Henry reaction/cyclization protocol.^18^ To this end, nitronate **16a** was traced back to commercially available 2-hydroxymethylenecyclohexanone (**17**).

We began our forward synthesis with the bromination of compound **17** (Scheme 3A), which was obtained on large scale from the formylation of cyclohexanone in 89% yield. The use of *N*-bromosuccinimide (NBS) afforded the crude α-bromo ketoaldehyde in excellent yields; however, we found it to be air-sensitive. For this reason, we decided to telescope the following step and conduct the overall process as a one-pot reaction. According to the seminal report of Rosini,^18^ the intermediate bromide was treated with ethyl nitroacetate and triethylamine to give nitronate **16a** and its C7 diastereomer **16b** as a 3:1 mixture as determined from the crude ^1^H NMR (see Supporting Information for details). After chromatographic separation of the highly polar **16b**, we obtained nitronate **16a** in 51% yield. The relative configuration of the desired diastereomer **16a** was validated by single crystal X-ray analysis. Performing the reaction in acetonitrile at −25 °C was crucial, as lower diastereoselectivities were observed when performing the reaction at higher temperatures or changing the solvent to methanol in which an equimolar mixture of nitronates **16a** and **16b** was obtained. We then proceeded with the deoxygenation of nitronate **16a** by employing triethyl phosphite in 1,4-dioxane at 100 °C to isolate the spirocyclic isoxazoline **18** in up to 99% yield.

**Scheme 3:**
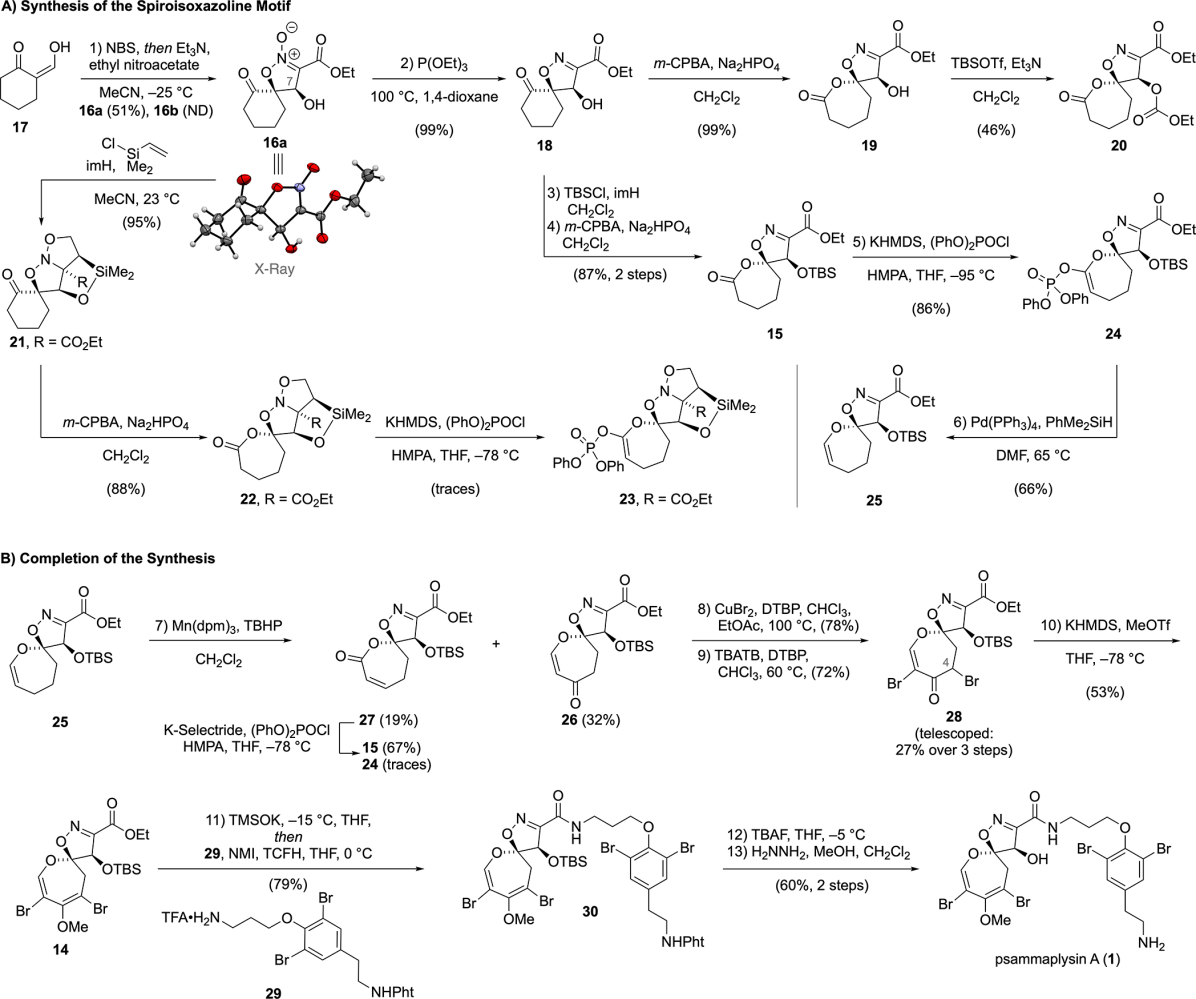
Synthesis of Psammaplysin A See the Supporting Information for detailed procedures and characterization data.

Having installed the 4-hydroxyisoxazoline motif and with multigram quantities of ketone **18** in hand, our attention was directed toward investigation of the Baeyer–Villiger oxidation to enable installation of the DOSI motif. We were pleased to see that exposure of ketone **18** to *m*-chloroperoxybenzoic acid (*m-*CPBA) in the presence of disodium hydrogen phosphate afforded the corresponding lactone **19** in nearly quantitative yield. To our surprise, the protection of the hydroxy group in intermediate **19** with *tert*-butyldimethylsilyl trifluoromethanesulfonate (TBSOTf) and triethylamine failed to give the desired silyl ether.^19^ Instead, formation of carbonate **20** was observed. This product might be derived from the reaction of lactone **19** with ethyl cyanoformate, which could form via decomposition of lactone **19**. As a part of the biosynthesis of ceratinamine A, a similar isoxazoline fragmentation was studied by Ganem.^16a^

In parallel, we took advantage of the reaction of nitronate **16a** with dimethylvinylsilyl chloride in the presence of imidazole.^20^ Under these conditions, clean silylation of the C7 hydroxy group followed by intramolecular 1,3-dipolar cycloaddition of the vinyl group with the nitronate took place to furnish the tricyclic acetal **21** in 95% yield. This motif serves a dual role as it (1) protects the hydroxy group and (2) enables one-step unmasking of the isoxazoline as described by Rosini.^20^ The following Baeyer–Villiger oxidation provided lactone **22** as the only regioisomer in 88% yield. However, subsequent attempts to form the ketene acetal phosphate **23** by employing Nicolaou’s conditions (diphenyl chlorophosphate, KHMDS, THF, HMPA, −78 °C),^21^ were low yielding due to decomposition of the product during isolation.

For this reason, we returned to isoxazoline **18** and protected the hydroxy group (imH, TBSCl) prior to the ring expansion. The subsequent Baeyer–Villiger oxidation proceeded with excellent regioselectivity and furnished the desired lactone **15** in 87% yield over two steps on a 6-g scale. When performing the following ketene acetal phosphate formation at −78 °C, varying yields between 36–67% of **24** were obtained and large amounts of lactone **15** were recovered. After further optimization, we found that slow addition of potassium hexamethyldisilazide (KHMDS) solution in THF at −95 °C was crucial for complete consumption of the starting material and to give ketene acetal phosphate **24** in reproducible 86% yield.

The palladium-catalyzed reduction of the ketene acetal phosphate to enol ether **25** also required some optimization. The use of Pd(PPh_3_)_4_ and Et_3_Al gave enol ether **25** as a complex mixture together with an unstable byproduct resulting from a competing cross-coupling reaction with Et_3_Al. We then switched to LiBH_4_ as the reductant;^21^ however, this led to competing reduction of the ester moiety. The chemoselectivity issue could be avoided by replacing LiBH_4_ with formic acid/triethylamine buffer. This allowed for clean conversion of ketene acetal phosphate **24** to tetrahydrooxepine-spiroisoxazoline **25** for the first time. Further screening revealed PhMe_2_SiH as the ideal reagent to give enol ether **25** in 66% yield.

With a scalable route to enol ether **25** in hand, we continued with the investigation of its sequential functionalization (Scheme 3B). We exposed enol ether **25** to a panel of oxidation conditions, most of which employed *tert*-butyl hydroperoxide (TBHP) in combination with a transition metal catalyst (CuBr; CuI; RuCl_3_; Mn(OAc)_3_; Mn(dpm)_3_; Pd(OH)_2_/C; see Supporting Information for details).^22^ The screening revealed that the desired vinylogous lactone **26** was formed under most conditions accompanied only by its regioisomeric α,β-unsaturated lactone **27**. The use of Pd(OH)_2_/C as the catalyst afforded vinylogous lactone **26** and lactone **27** as a 2.5:1 mixture of regioisomers; however, the reaction suffered from low conversion. The best performing conditions in terms of conversion involved Mn(dpm)_3_ and dropwise addition of TBHP overnight to give **26** in 32% isolated yield. Direct conversion of the undesired regioisomer **27** (19%) to ketene acetal phosphate **24** was unsuccessful in our hands but allowed for the isolation of lactone **15** in 67% yield.

Subsequent α-bromination of vinylogous lactone **26** with CuBr_2_ and 2,6-di-*tert*-butylpyridine (DTBP) in a mixture of chloroform and ethyl acetate at 100 °C proceeded cleanly to deliver the C4-brominated product in 78% yield. The second bromination was accomplished upon exposure to tetra-*n*-butylammonium tribromide (TBATB) and DTBP at 60 °C in chloroform to afford vinylogous lactone **28** in 72% yield.^10b^ We found that the overall yield of vinylogous lactone **28** could be improved by telescoping the three oxidations without chromatographic purification of the intermediates. In this way, we obtained vinylogous lactone **28** in 27% yield over three steps from **25**. Finally, sequential treatment of vinylogous lactone **28** with KHMDS and methyl trifluoromethanesulfonate (MeOTf) gave the fully substituted DOSI **14** in 53% yield.

For the late-stage attachment of the protected moloka′iamine unit **29**, which was prepared from tyramine in four steps (see Supporting Information), we first attempted conversion of ethyl ester **14** into the corresponding carboxylic acid. Saponification under aqueous conditions (LiOH, THF, H_2_O, 23 °C) followed by isolation of the acid turned out be impossible in our hands as decomposition, presumably via decarboxylation, was observed even at low temperatures.^23^ For this reason, we decided to treat ester **14** with potassium trimethylsilanolate (TMSOK) at −15 °C under nonaqueous conditions followed by direct addition of ammonium salt **29**, *N*-methylimidazole (NMI), and chloro-*N*,*N*,*N*′,*N*′-tetramethylformamidinium hexafluorophosphate (TCFH).^10b^ This one-pot procedure bypassed the problematic isolation of the acid and allowed us to obtain the desired amide **30** in 79% yield from ester **14**. The following deprotections of the C7 oxygen and C20 nitrogen proceeded smoothly to furnish psammaplysin A (**1**) in 60% yield. The analytical data (^1^H NMR, ^13^C NMR, HRMS) for **1** and its bisacetylated derivative fully matched those reported for the natural compound.^4^ Overall, psammaplysin A (**1**) was synthesized in 13 steps from commercially available starting materials.

In summary, we have accomplished the first synthesis of psammaplysin A (**1**) nearly 40 years after its first isolation. The key to success of the developed strategy was the use of a Henry addition/O-alkylation sequence instead of a nitrile oxide [3 + 2]-cycloaddition. This granted access to the fully substituted C7 hydroxylated heterocyclic scaffold in one step. For the construction of the 7-membered ring-system a high-yielding Baeyer–Villiger oxidation turned out to be the method of choice. This avoided the risk of a previously encountered oxepine-arene oxide rearrangement and paved the way for the selective installation of the fully intact DOSI motif. The late-stage attachment of the side chain ensures the high flexibility and modularity of the synthesis. This approach should allow for the preparation of several structurally related members as well as analogs with deep-seated structural modifications and facilitate future bioactivity studies.

## Supporting Information Available

Experimental details, spectroscopic data, and X-ray data (PDF)
