# Supplementary material for: Total Synthesis of the Dihydrooxepine-Spiroisoxazoline Natural Product Psammaplysin A
Source: J Am Chem Soc. 2022 Oct 21;144(43):19704–8. doi: 10.1021/jacs.2c10010 (PMC9634798; doi:10.1021/jacs.2c10010)
Supplement: Supplementary file 1 — ja2c10010_si_001.pdf [file ja2c10010_si_001.pdf]

Supporting Information

Total Synthesis of the Dihydrooxepine-Spiroisoxazoline Natural Product Psammaplysin A

Jan Paciorek†, Denis Höfler†, Kevin Rafael Sokol†, Klaus Wurst§, Thomas Magauer*,†

†Institute of Organic Chemistry and Center for Molecular Biosciences, Leopold-Franzens-University Innsbruck, Innrain 80–82, 6020 Innsbruck, Austria

§Institute of General, Inorganic & Theoretical Chemistry, Leopold-Franzens-University Innsbruck, Innrain 80–82, 6020 Innsbruck, Austria

Corresponding author: thomas.magauer@uibk.ac.at

Content

General Information S1
Experimental Procedures S3
Comparison of Natural and Synthetic Psammaplysin A S41
NMR Spectra S46
Crystallographic Data S86
References S89

General Information

Unless otherwise noted, all reactions were magnetically stirred and carried out in oven-dried glassware fitted with rubber septa. Air– and moisture–sensitive liquids were transferred via syringes through the rubber septa. Solids were added either using standard Schlenk techniques or were dissolved in appropriate solvents. Reactions performed at low temperatures were cooled by immersing reaction flasks in an acetone bath cooled to the desired temperature by a cryostat, or in a mixture of acetone/dry ice (–78 °C), acetone/liquid nitrogen (–95 °C), water/ice (0 °C). Reactions at temperatures above 23 °C were performed in flasks placed in an oil bath or an aluminum metal block heated to the appropriate temperature. The reactions were monitored by NMR spectroscopy or thin-layer chromatography (TLC) using aluminum sheets precoated with silica gel (Merck, 0.25 mm, 60 Å pore, impregnated with fluorescent indicator). The TLC plates were visualized by exposure to UV light (254 nm) or by immersing into an aqueous potassium permanganate staining solution followed by heating with a heat gun. Flash chromatography was performed using Merck silica gel 60 (0.040–0.063 mm).

Reagents were obtained from commercial sources (Sigma Aldrich, TCI, BLDpharm, Fisher etc.) and were used as such without further purification unless otherwise noted. Reaction grade solvents were obtained from commercial sources (Acros Organics, Thermo Fisher, Sigma Aldrich) as extra dry and were transferred to reaction mixtures under argon via syringe. Tetrahydrofuran and diethyl ether were additionally dried over 4Å molecular sieves. Solvents for flash chromatography were obtained as crude and were distilled under reduced pressure prior to use.

NMR spectra (^{1}H NMR, ^{13}C NMR, ^{31}P NMR) were recorded in deuterated chloroform ($CDCl_3$), dichloromethane (CD_2Cl_2), benzene (C_6D_6), methanol (CD_3OD) or dimethylsulfoxide (DMSO-d_6) on a Bruker Avance Neo 400MHz spectrometer and are reported as follows: chemical shift δ in ppm (multiplicity, coupling constant J in Hz, number of protons) for ^{1}H NMR spectra and chemical shift δ in ppm for ^{13}C NMR, ^{19}F and ^{31}P NMR spectra. Multiplicities are abbreviated as follows: s = singlet, d = doublet, t = triplet, q = quartet, m = multiplet, br = broad, or combinations thereof. Residual solvent peaks of $CDCl_3$ (δ_H = 7.26 ppm, δ_C = 77.16), CD_2Cl_2 (δ_H = 5.32 ppm, δ_C = 53.84), C_6D_6 (δ_H = 7.16 ppm, δ_C = 128.06 ppm), CD_3OD (δ_H = 3.31 ppm, δ_C = 49.00 ppm) and DMSO-d_6 (δ_H =

2.50 ppm, δ_C = 39.52 ppm) were used as internal reference. The ^{19}F and ^{31}P NMR spectra are reported without a reference peak. NMR spectra were assigned using information ascertained from COSY, HMBC, HSQC and NOESY experiments.

Infrared spectra were recorded from 4000 cm^{-1} to 450 cm^{-1} on a Bruker™ ALPHA FT-IR Spectrometer. Samples were prepared as a neat film or a film by evaporation of a solution in $CDCl_3$, C_6D_6, CD_2Cl_2 or CD_3OD. IR data in wavenumber $\tilde{\nu}$ (cm^{-1}) is reported as follows: *w* = weak, *m* = medium, *s* = strong, *br* = broad or combinations thereof.

High-resolution mass spectra were recorded on a Thermo Scientific™ LTQ Orbitrap XL™ Hybrid Ion Trap-Orbitrap Mass Spectrometer at the Institute of Organic Chemistry and Center for Molecular Biosciences, University of Innsbruck.

Melting points were measured with an SRS MPA120 EZ-Melt Melting Point Apparatus in open glass capillaries and are uncorrected.

X-ray diffraction analysis was carried out by Prof. Dr. Klaus Wurst at the Institute of Inorganic and Theoretical Chemistry and Center for Molecular Biosciences, University of Innsbruck. The data collections were performed on a Bruker D8Quest using MoKα-radiation (λ = 0.71073 Å, Incoatec Microfocus). The Bruker Apex III software was applied for the integration, scaling and multi-scan absorption correction of the data. The structures were solved by direct methods with SHELXTL-XT-2014 and refined by least-squares methods against F^2 with SHELXL-2014/7. All non-hydrogen atoms were refined anisotropically. The hydrogen atoms were placed in ideal geometry riding on their parent atoms. Further details are summarized in the tables at the corresponding sections. Plotting of thermal ellipsoids in this document and in the main text was carried out using MERCURY for Windows at 50% probability level.

All yields were calculated from amounts of isolated pure products.

Experimental Procedures

Synthesis of esters S1a and S1b

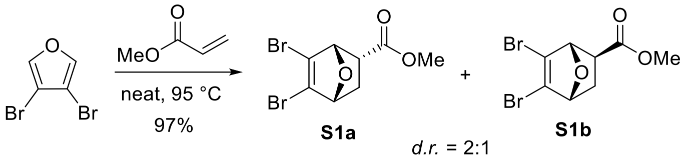

A mixture of 3,4-dibromofuran (5.00 g, 22.1 mmol, 1 equiv) and methyl acrylate (20 mL, 220 mmol, 10.0 equiv) was stirred at 95 °C in a pressure tube for 24 h. The solution was allowed to cool to 23 °C and excess methyl acrylate was removed under reduced pressure. The residue was purified by flash chromatography on silica gel (10% diethyl ether in petroleum ether) which afforded the product as a 2:1 mixture of diastereomers **S1a** and **S1b** (6.70 g, 21.5 mmol, 97%).

Note: In the next step the mixture of diastereomers was used. However, for characterization purposes an analytical sample of each diastereomer was prepared by flash chromatography of their mixture on silica gel with toluene as the eluent.

Characterization of ester **S1a** (major):

TLC (20% diethyl ether in pentane): R_f = 0.34 ($KMnO_4$).

^{1}H NMR (400 MHz, $CDCl_3$, 25 °C): δ 5.08 (dd, J = 4.8, 1.0 Hz, 1H), 4.92 (dd, J = 4.6, 1.0 Hz, 1H), 3.68 (s, 3H), 3.28 – 3.22 (m, 1H), 2.11 (ddd, J = 11.8, 8.9, 4.6 Hz, 1H), 1.98 (dd, J = 11.9, 3.9 Hz, 1H) ppm.

^{13}C NMR (101 MHz, $CDCl_3$, 25 °C): δ 170.5, 127.2, 122.1, 85.5, 85.1, 52.1, 44.5, 28.5 ppm.

IR (ATR, neat): $\tilde{\nu}$ = 2953 (*w*), 1738 (*s*), 1595 (*w*), 1436 (*w*), 1345 (*w*), 1293 (*w*), 1201 (*s*) 1179 (*m*), 1085 (*m*), 1025 (*w*), 972 (*w*), 894 (*m*), 854 (*w*), 815 (*m*) cm^{-1}.

HRMS (ESI): calcd. for $C_8H_8Br_2NaO_3$ [M+Na]$^+$: 332.8732; found: 332.8727.

Characterization of ester **S1b** (minor):

TLC (20% diethyl ether in pentane): R_f = 0.34 ($KMnO_4$).

^{1}H NMR (400 MHz, $CDCl_3$, 25 °C): δ 5.10 (d, J = 0.9 Hz, 1H), 4.93 (dd, J = 4.5, 0.9 Hz, 1H), 3.75 (s, 3H), 2.69 (dd, J = 8.7, 3.9 Hz, 1H), 2.28 – 2.22 (m, 1H), 1.86 (dd, J = 12.1, 8.7 Hz, 1H) ppm.

^{13}C NMR (151 MHz, $CDCl_3$, 25 °C): δ 172.5, 127.4, 124.5, 86.8, 84.5, 52.6, 43.7, 30.2 ppm.

IR (ATR, neat): $\tilde{\nu}$ = 2953 (*w*), 1734 (*s*), 1596 (*w*), 1435 (*w*), 1347 (*w*), 1281 (*m*), 1199 (*s*) 1169 (*m*), 1083 (*m*), 1046 (*w*), 931 (*w*), 889 (*m*), 867 (*m*), 817 (*m*), 781 (*w*) cm^{-1}.

HRMS (ESI): calcd. for $C_8H_8Br_2NaO_3$ [M+Na]$^+$: 332.8732; found: 332.8727.

Synthesis of ester S2

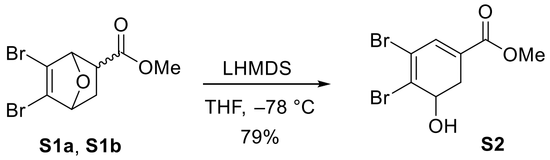

To a stirred solution of esters **S1a** and **S1b** (6.7 g, mixture of diastereomers 2:1, 21 mmol, 1 equiv) in tetrahydrofuran (200 mL) at –78 °C was added dropwise a solution of lithium bis(trimethylsilyl)amide (1.0 M in tetrahydrofuran, 30 mL, 30 mmol, 1.4 equiv) over 10 min and the resulting solution was stirred at –78 °C for 3 h. A saturated aqueous ammonium chloride solution (80 mL) was added and the mixture was warmed up to 23 °C. The mixture was extracted with diethyl ether (4 × 100 mL). The combined organic solutions were dried over sodium sulfate and filtered. The dried filtrate was concentrated under reduced pressure and the residue was purified by flash chromatography on silica gel (20% diethyl ether in pentane grading to 40% diethyl ether in pentane) which afforded the ester **S2** as a colorless amorphous solid (5.3 g, 17 mmol, 79%).

TLC (20% diethyl ether in petroleum ether): R_f = 0.31 (UV, $KMnO_4$).

^{1}H NMR (400 MHz, $CDCl_3$, 25 °C): δ 7.11 (dd, J = 2.9, 0.5 Hz, 1H), 4.57 (br m, 1H), 3.80 (s, 3H), 3.02 (ddd, J = 19.2, 3.3, 0.6 Hz, 1H), 2.76 (ddd, J = 19.2, 7.1, 2.9 Hz, 1H), 2.27 (br d, J = 5.0 Hz, 1H) ppm.

^{13}C NMR (101 MHz, $CDCl_3$, 25 °C): δ 166.1, 134.7, 130.8, 127.5, 121.6, 71.6, 52.4, 31.7 ppm.

IR (ATR, neat): $\tilde{\nu}$ = 3420 (*w*, *br*), 2951 (*w*), 1711 (*s*), 1624 (*w*), 1557 (*m*), 1436 (*m*), 1383 (*w*), 1268 (*s*), 1204 (*m*), 1143 (*w*), 1099 (*m*), 1038 (*m*), 964 (*w*), 861 (*w*), 748 (*w*), 677 (*w*) cm^{-1}.

HRMS (ESI): calcd. for $C_8H_7Br_2O_2$ $[M{-}H_2O{+}H]^+$: 292.8807; found: 292.8795.

Synthesis of ester S3

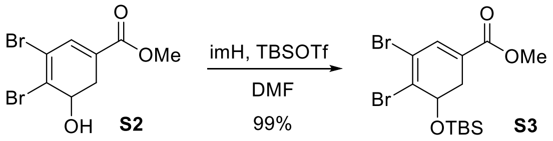

To a solution of ester **S2** (5.3 g, 17 mmol, 1 equiv) in *N*,*N*-dimethylformamide (100 mL) was added imidazole (1.7 g, 25 mmol, 1.5 equiv) followed by dropwise addition of *tert*-butyldimethylsilyl trifluoromethanesulfonate (9.0 g, 34 mmol, 2.0 equiv) over 15 min at 23 °C. The resulting solution was stirred for 20 h at 23 °C. A saturated aqueous ammonium chloride solution (60 mL) was added and the mixture was extracted with diethyl ether (60 mL). The organic solution was washed with a saturated aqueous sodium chloride solution (60 mL). The aqueous solution was extracted with diethyl ether (20 mL). The combined organic solutions were dried over sodium sulfate and filtered. The dried filtrate was concentrated under reduced pressure and the residue was purified by flash chromatography on silica gel (10% diethyl ether in petroleum ether) which afforded the ester **S3** as a colorless solid (7.2 g, 17 mmol, 99%).

TLC (20 % diethyl ether in petroleum ether): R_f = 0.67 ($KMnO_4$).

Melting point: 52 – 54 °C.

^{1}H NMR (400 MHz, $CDCl_3$, 25 °C): δ 7.09 (d, *J* = 2.8 Hz, 1H), 4.53 (dd, *J* = 6.6, 3.8 Hz, 1H), 3.79 (s, 3H), 2.88 (dd, *J* = 18.6, 3.8 Hz, 1H), 2.65 (ddd, *J* = 18.6, 6.6, 2.8 Hz, 1H), 0.87 (s, 9H), 0.14 (s, 3H), 0.12 (s, 3H) ppm.

^{13}C NMR (151 MHz, $CDCl_3$, 30 °C): δ 166.3, 135.0, 131.7, 127.4, 121.1, 72.1, 52.3, 33.0, 25.8, 18.3, –4.3, –4.5 ppm.

IR (ATR, neat): $\tilde{\nu}$ = 2952 (*w*), 2856 (*w*), 1716 (*s*), 1559 (*w*), 1436 (*w*), 1314 (*w*), 1254 (*s*), 1205 (*m*), 1076 (*m*), 1049 (*m*), 984 (*w*), 936 (*w*), 836 (*m*), 778 (*m*) cm^{-1}.

HRMS (ESI): calcd. for $C_{14}H_{23}Br_2O_3Si$ $[M+H]^+$: 424.9778; found: 424.9764.

Synthesis of alcohols S4 and S5

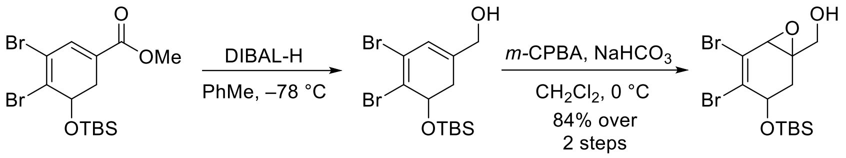

To a stirred solution of ester **S3** (7.20 g, 16.9 mmol, 1 equiv) in toluene (60 mL) cooled to –78 °C was added dropwise a solution of diisobutylaluminium hydride (1.00 M in toluene, 40.0 mL, 40.0 mmol, 2.37 equiv) over 15 min. The resulting solution was stirred for 30 min before ethyl acetate (60 mL) was added at –78 °C. A saturated aqueous sodium potassium tartrate solution (60 mL) was added and the mixture was allowed to warm up to 23 °C. After stirring for 1 h, the organic solution was separated and washed with a saturated aqueous sodium chloride solution (2 × 50 mL). The washed solution was dried over sodium sulfate and filtered. The dried filtrate was concentrated under reduced pressure which afforded crude, unstable alcohol **S4** (6.70 g, 16.8 mmol) which was dissolved in dichloromethane (150 mL). To the stirred solution was added solid sodium bicarbonate (7.07 g, 84.1 mmol, 4.98 equiv), the mixture was cooled to 0 °C and 3-chloroperoxybenzoic acid (4.53 g, 77 wt%, 20.2 mmol, 1.20 equiv) was added. The resulting mixture was stirred at 0 °C for 30 min before a 1 M aqueous sodium thiosulfate solution (150 mL) was added. The organic solution was separated and washed with 1 M aqueous sodium thiosulfate solution (150 mL). The combined aqueous solutions were extracted with dichloromethane (100 mL). The combined organic solutions were dried over sodium sulfate and filtered. The dried filtrate was concentrated under reduced pressure and the residue was purified by flash chromatography on silica gel (30% diethyl ether in petroleum ether) which afforded the alcohol **S5** as a colorless solid (5.90 g, 14.2 mmol, 84% over 2 steps).

TLC (50 % diethyl ether in petroleum ether): R_f = 0.45 (UV, $KMnO_4$).

Melting point: 74 – 76 °C.

^{1}H NMR (400 MHz, $CDCl_3$, 25 °C): δ 4.57 (dd, J = 5.6, 2.7 Hz, 1H), 3.86 – 3.77 (m, 2H), 3.75 – 3.63 (br m, 1H), 2.26 (ddd, J = 15.4, 2.6, 0.9 Hz, 1H), 2.11 (dd, J = 15.4, 5.6 Hz, 1H), 1.69 (br s, 1H), 0.91 (s, 9H), 0.16 (s, 3H), 0.13 (s, 3H) ppm.

^{13}C NMR (101 MHz, $CDCl_3$, 25 °C): δ 131.2, 122.9, 71.9, 64.2, 63.0, 57.6, 33.7, 25.9, 18.2, –4.1, –4.4 ppm.

IR (ATR, neat): $\tilde{\nu}$ = 3429 (*w*, *br*), 2953 (*s*), 2929 (*s*), 2893 (*m*), 2856 (*s*), 1613 (*w*), 1471 (*m*), 1413 (*w*), 1362 (*m*), 1255 (*s*), 1093 (*s*), 1003 (*m*), 904 (*w*), 871 (*s*), 837 (*s*), 811 (*s*), 778 (*s*) cm^{-1}.

HRMS (ESI): calcd. for $C_{13}H_{22}Br_2NaO_3Si$ [M+Na]$^+$: 434.9597; found: 434.9594.

Synthesis of diol 5

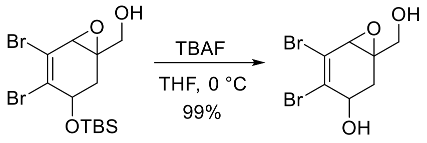

S5 **5**

To a solution of alcohol **S5** (1.00 g, 2.41 mmol, 1 equiv) in tetrahydrofuran (60 mL) was added a solution of tetra-*n*-butylammonium fluoride (1.00 M in tetrahydrofuran, 2.90 mL, 2.90 mmol, 1.20 equiv) at 0 °C and the resulting solution was stirred at that temperature for 40 min. Diethyl ether (80 mL) was added and the mixture was washed with water (4 × 50 mL). To the combined aqueous solutions was added a saturated aqueous sodium chloride solution (60 mL). The resulting aqueous solution was extracted with ethyl acetate (2 × 50 mL). The combined organic solutions were dried over sodium sulfate and filtered. The dried filtrate was concentrated under reduced pressure and the residue was purified by flash chromatography on silica gel (0% methanol in dichloromethane grading to 4% methanol in dichloromethane) which afforded the diol **5** as a colorless oil (715 mg, 2.40 mmol, 99%).

TLC (80 % diethyl ether in petroleum ether): R_f = 0.29 (UV, $KMnO_4$).

^{1}H NMR (400 MHz, C_6D_6, 25 °C): δ 4.04 (ddd, J = 12.0, 4.8, 1.9 Hz, 1H), 3.59 (d, J = 0.8 Hz, 1H), 3.02 (dd, J = 12.9, 3.9 Hz, 1H), 2.89 (dd, J = 12.8, 7.2 Hz, 1H), 2.60 (d, J = 12.1 Hz, 1H), 1.82 (ddd, J = 15.5, 1.8, 1.0 Hz, 1H), 1.06 – 0.99 (m, 1H), 0.96 (dd, J = 15.5, 4.8 Hz, 1H) ppm.

^{13}C NMR (101 MHz, C_6D_6, 25 °C): δ 131.3, 122.6, 72.7, 65.9, 62.3, 59.4, 31.2 ppm.

IR (ATR, neat): $\tilde{\nu}$ = 3387 (*w*, *br*), 2291 (*w*, *br*), 1610 (*m*), 1408 (*m*), 1135 (*m*), 1056 (*s*), 1023 (*m*), 967 (*w*), 845 (*m*), 808 (*m*) cm^{-1}.

HRMS (ESI): calcd. for $C_7H_8Br_2NaO_3$ [M+Na]$^+$: 320.8732; found: 320.8727.

Synthesis of alcohol S6 and aldehyde 7

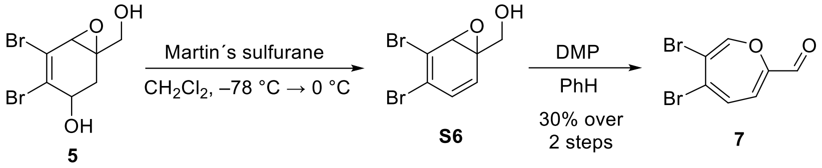

To a solution of Martin´s sulfurane (3.92 g, 5.83 mmol, 4.98 equiv) in dichloromethane (50 mL) at –78 °C was added a solution of diol **5** (0.78 M in dichloromethane, 1.5 mL, 1.17 mmol, 1 equiv). The resulting solution was stirred for 15 min before it was allowed to warm up to 0 °C. After 30 min, a saturated aqueous sodium bicarbonate solution (30 mL) was added, the organic layer was separated and washed with a saturated aqueous sodium chloride solution (150 mL). The washed solution was dried over sodium sulfate and filtered. The dried filtrate was concentrated under reduced pressure which afforded crude alcohol **S6.** Intermediate **S6** was dissolved in benzene (12 mL). The resulting solution was added to solid Dess–Martin periodinane (614 mg, 1.40 mmol, 1.20 equiv) and the resulting slurry was stirred for 5 min. A saturated aqueous sodium bicarbonate solution (1 mL) and a saturated aqueous thiosulfate solution (1 mL) were added. The mixture was extracted with diethyl ether (20 mL). The organic solution was washed with water (3 × 10 mL) and a saturated aqueous sodium chloride solution (10 mL). The washed solution was dried over sodium sulfate and filtered. The dried filtrate was concentrated under reduced pressure and the residue was purified by flash chromatography on silica gel (0% diethyl ether in petroleum ether grading to 2% diethyl ether in petroleum ether) which afforded aldehyde **7** as an orange solid (97 mg, 0.35 mmol, 30% over two steps).

Crystals of aldehyde **7** for single crystal X-ray crystallography were prepared as follows: 47 mg of aldehyde **7** were dissolved in heptane and the solution was stored in a freezer at –25 °C overnight which afforded crystals suitable for X-ray analysis.

TLC (30% diethyl ether in petroleum ether): R_f = 0.12 (UV, $KMnO_4$).

Melting point: 60 – 61 °C.

^{1}H NMR (400 MHz, CD_2Cl_2, 25 °C): δ 9.26 (s, 1H), 6.92 (d, J = 7.2 Hz, 1H), 6.55 (d, J = 7.2 Hz, 1H), 5.94 (s, 1H) ppm.

^{13}C NMR (101 MHz, CD_2Cl_2, 25 °C): δ 187.8, 132.8, 129.0, 127.6, 127.2, 121.8, 117.1 ppm.

IR (ATR, neat): $\tilde{\nu}$ = 2922 (*m*), 2851 (*m*), 1730 (*m*), 1694 (*s*), 1586 (*w*), 1546 (*m*), 1165 (*w*), 1130 (*m*), 1069 (*w*), 1011 (*s*), 830 (*m*), 813 (*m*), 759 (*m*) cm^{-1}.

HRMS (ESI negative): calcd. for $C_9H_7Br_2O_4$ [M+CH_3COO]$^-$: 336.8717; found: 336.8712.

Synthesis of acid 8a and ester 9

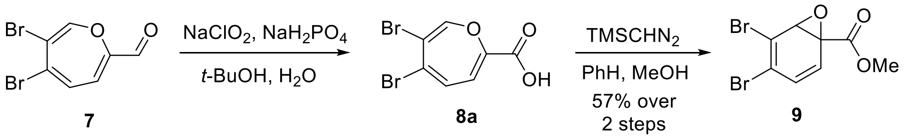

To a solution of **7** (30 mg, 0.11 mmol, 1 equiv) and sodium dihydrogen phosphate (29 mg, 0.21 mmol, 2.0 equiv) in a mixture of *tert*-butanol (0.50 mL), 2-methyl-2-butene (0.16 mL) and water (0.16 mL) was added sodium chlorite (36 mg, 80 wt%, 0.32 mmol, 3.0 equiv) at 0 °C. The resulting mixture was stirred at 0 °C for 4 h before a saturated aqueous ammonium chloride solution (10 mL) was added. The mixture was extracted with diethyl ether (4 × 20 mL) and ethyl acetate (3 × 30 mL). The combined organic solutions were dried over sodium sulfate and filtered. The dried filtrate was concentrated under reduced pressure which afforded a pale-yellow solid **8a** that was dissolved in a mixture of benzene (0.7 mL) and methanol (0.2 mL). To this solution was added a solution of trimethylsilyldiazomethane (2.0 M in hexanes, 64 μL, 0.13 mmol, 1.2 equiv) at 22 °C and the resulting solution was stirred for 5 min. Acetic acid (0.1 mL) was added and the mixture was concentrated under reduced pressure. Flash chromatography of the residue on silica gel (0% diethyl ether in petroleum ether grading to 3% diethyl ether in petroleum ether) afforded ester **9** as a colorless solid (19 mg, 61 μmol, 57% over 2 steps).

Crystals of ester **9** for single crystal X-ray crystallography were prepared as follows: 5 mg of ester **9** were dissolved in 1.5 mL of diethyl ether and the solution was stored in a freezer at –25 °C overnight which afforded crystals suitable for X-ray analysis.

TLC (20% diethyl ether in petroleum ether): R_f = 0.57 (UV, $KMnO_4$).

Melting point: 95 – 97 °C.

^{1}H NMR (300 MHz, $CDCl_3$, 25 °C): δ 6.77 (d, J = 8.8 Hz, 1H), 6.70 (d, J = 8.8 Hz, 1H), 5.22 (s, 1H), 3.85 (s, 3H) ppm.

^{13}C NMR (75 MHz, $CDCl_3$, 25 °C): δ 165.8, 132.9, 125.8, 124.2, 119.9, 88.5, 53.3 ppm.

IR (ATR, neat): $\tilde{\nu}$ = 2955 (*w*), 1737 (*s*), 1438 (*m*), 1308 (*m*), 1276 (*m*), 1146 (*m*), 1120 (*m*), 1060 (*m*), 860 (*w*), 791 (*w*), 758 (*m*), 715 (*w*) cm^{-1}.

HRMS (ESI): calcd. for $C_8H_6Br_2NaO_3$ [M+Na]$^+$: 330.8576 ; found: 330.8570.

Synthesis of dienone S7

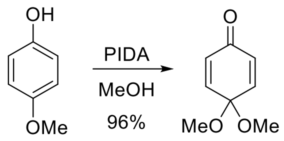

S7

To a solution of 4-methoxyphenol (2.10 g, 16.9 mmol, 1 equiv) in methanol (40 mL) was added solid bis(acetoxy)iodobenzene (5.54 g, 17.2 mmol, 1.02 equiv) in several portions over 5 min at 23 °C and the resulting solution was stirred for 15 min. Methanol was removed under reduced pressure and diethyl ether (40 mL) was added. To the resulting, vigorously stirred solution was slowly added a saturated aqueous sodium bicarbonate solution (40 mL). After evolution of carbon dioxide ceased, the ether layer was separated and the aqueous layer was extracted with diethyl ether (2 × 40 mL). The combined organic solutions were washed with a saturated aqueous sodium chloride solution (80 mL). The washed solution was dried over magnesium sulfate and filtered. The dried filtrate was concentrated under reduced pressure and the residue was purified by flash chromatography on silica gel (20% diethyl ether and 1% triethylamine in pentane) which afforded the dienone **S7** as a yellow oil (2.50 g, 16.2 mmol, 96%).

^{1}H NMR (400 MHz, $CDCl_3$, 25 °C): δ 6.84 – 6.79 (m, 2H), 6.29 – 6.23 (m, 2H), 3.36 (s, 6H) ppm. The analytical data matched those published before.[1]

Synthesis of ketone S8

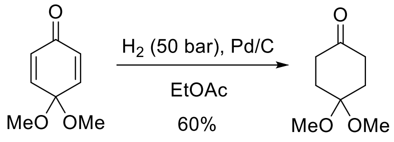

S7 **S8**

To a solution of dienone **S7** (2.50 g, 16.2 mmol, 1 equiv) in ethyl acetate (10 mL) was added palladium on charcoal (200 mg, 10 wt% of Pd, 1 mol%), the flask was put into a stainless-steel high-pressure chamber which was then purged with hydrogen gas (three cycles of pressurizing to 50 bar followed by releasing the pressure). The chamber was pressurized to 50 bar with hydrogen gas and the reaction mixture was stirred at 23 °C for 20 h. The pressure was released and the reaction mixture was filtered. The filtrate was concentrated under reduced pressure and the residue was purified by flash chromatography on silica gel (20% ethyl acetate and 2% triethylamine in pentane) which afforded the ketone **S8** as a colorless oil (1.55 g, 9.80 mmol, 60%).

^{1}H NMR (400 MHz, $CDCl_3$, 25 °C): δ 3.27 (s, 6H), 2.42 – 2.36 (m, 4H), 2.06 – 2.00 (m, 4H) ppm. The analytical data matched those published before.[2]

Synthesis of lactone S9

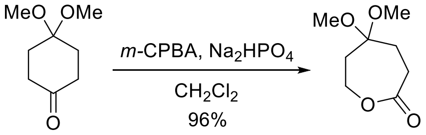

S8 **S9**

To a solution of ketone **S8** (403 mg, 2.55 mmol, 1 equiv) in dichloromethane (10 mL) was added solid disodium hydrogen phosphate (800 mg, 5.64 mmol, 2.21 equiv) and 3-chloroperoxybenzoic acid (1.26 g, 70 wt%, 5.10 mmol, 2.00 equiv) in one portion and the resulting suspension was stirred for 20 h at 23 °C. Water (20 mL) and dichloromethane (10 mL) were added. The organic layer was separated and washed with a saturated aqueous sodium thiosulfate solution (20 mL) and a saturated aqueous sodium bicarbonate solution (20 mL). The combined aqueous solutions were extracted with dichloromethane (40 mL). The combined organic solutions were dried over magnesium sulfate and filtered. The dried filtrate was concentrated under reduced pressure to afford

the lactone **S9** as a colorless solid (426 mg, 2.45 mmol, 96%) which did not require further purification.

TLC (40% ethyl acetate in cyclohexane): R_f = 0.25 ($KMnO_4$).

Melting point: 86 – 87 °C.

^{1}H NMR (400 MHz, $CDCl_3$, 25 °C): δ 4.27 – 4.22 (m, 2H), 3.19 (s, 6H), 2.65 – 2.59 (m, 2H), 2.06 – 2.00 (m, 2H), 1.97 – 1.91 (m, 2H) ppm.

^{13}C NMR (101 MHz, $CDCl_3$, 25 °C): δ 175.6, 99.7, 64.0, 48.1, 36.4, 29.6, 28.3 ppm.

IR (ATR, neat): $\tilde{\nu}$ = 2968 (*w*), 2837 (*w*), 1716 (*s*), 1449 (*m*), 1333 (*m*), 1297 (*m*), 1248 (*m*), 1158 (*s*), 1112 (*s*), 1052 (*s*), 1017 (*s*), 915 (*m*), 885 (*s*) cm^{-1}.

HRMS (ESI): calcd. for $C_8H_{15}O_4$ [M+H]$^+$: 175.0965; found: 175.0964.

Synthesis of enol ether S10

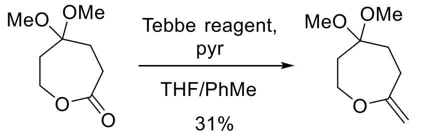

S9 S10

To a solution of lactone **S9** (0.21 g, 1.2 mmol, 1 equiv) in tetrahydrofuran (1 mL) was added pyridine (0.20 mL, 2.4 mmol, 2.0 equiv) and the solution was cooled to 0 °C. Solution of Tebbe reagent (0.50 M in toluene, 2.9 mL, 1.5 mmol, 1.2 equiv) was added and the resulting solution was stirred for 2 h at 0 °C. A 2 M aqueous sodium hydroxide solution (1 mL) was added dropwise over 10 min. After the bubbling ceased, diethyl ether (10 mL) was added. The reaction mixture was filtered through a pad of celite which was then washed with diethyl ether (2 × 10 mL). The resulting solution was concentrated under reduced pressure and the residue was purified by flash chromatography on silica gel (10% diethyl ether and 2% triethylamine in pentane) which afforded the enol ether **S10** as a pale-yellow oil (64 mg, 0.37 mmol, 31%).

TLC (30% ethyl acetate in cyclohexane): R_f = 0.64 (weak UV, $KMnO_4$).

^{1}H NMR (400 MHz, $CDCl_3$, 25 °C): δ 4.09 (s, 1H), 3.97 – 3.93 (m, 2H), 3.88 (s, 1H), 3.18 (s, 6H), 2.39 – 2.34 (m, 2H), 1.96 – 1.92 (m, 2H), 1.90 – 1.85 (m, 2H) ppm.

^{13}C NMR (101 MHz, $CDCl_3$, 25 °C): δ 163.3, 100.8, 87.2, 62.5, 48.1, 37.8, 34.1, 25.7 ppm.

IR (ATR, neat): $\tilde{\nu}$ = 2959 (*m*), 2830 (*w*), 1719 (*w*), 1658 (*w*), 1438 (*w*), 1339 (*w*), 1295 (*w*), 1258 (*w*), 1188 (*w*), 1145 (*w*), 1115 (*s*), 1100 (*m*), 1057 (*m*), 914 (*w*), 876 (*w*), 814 (*w*) cm^{-1}.

HRMS (ESI): calcd. for $C_8H_{13}O_2$ $[M–CH_3OH+H]^+$: 141.0910; found: 141.0906.

Synthesis of ester 10

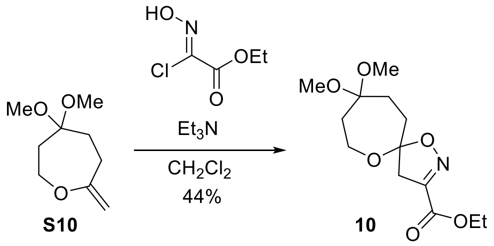

To a solution of enol ether **S10** (60 mg, 0.35 mmol, 1 equiv) in dichloromethane (1 mL) were added triethylamine (0.15 mL, 1.0 mmol, 2.9 equiv) and ethyl 2-chloro-2-(hydroxyimino)acetate (0.15 g, 1.0 mmol, 2.9 equiv) at 23 °C and the resulting solution was stirred for 20 h. Ethyl acetate (10 mL) was added and the resulting solution was washed with a saturated aqueous sodium bicarbonate solution (10 mL), water (10 mL) and a saturated aqueous sodium chloride solution (10 mL). The washed solution was dried over magnesium sulfate and filtered. The dried filtrate was concentrated under reduced pressure and the residue was purified by flash chromatography on silica gel (15% diethyl ether and 1% triethylamine in pentane grading to 30% diethyl ether and 1% triethylamine in pentane) which afforded the ester **10** as a colorless solid (44 mg, 0.15 mmol, 44%).

TLC (30 % ethyl acetate in cyclohexane): R_f = 0.42 (UV, $KMnO_4$).

Melting point: 54 – 56 °C.

^{1}H NMR (400 MHz, $CDCl_3$, 25 °C): δ 4.37 – 4.30 (m, 2H), 4.03 – 3.94 (m, 1H), 3.64 – 3.57 (m, 1H), 3.22 – 3.14 (m, 7H), 3.01 (d, *J* = 18.1 Hz, 1H), 2.44 (dd, *J* = 15.5, 11.8 Hz, 1H), 2.10 – 1.87 (m, 3H), 1.80 – 1.71 (m, 1H), 1.60 (dd, *J* = 14.4, 12.0 Hz, 1H), 1.35 (t, *J* = 7.1 Hz, 3H) ppm.

^{13}C NMR (101 MHz, $CDCl_3$, 25 °C): δ 160.7, 152.6, 114.7, 101.1, 62.2, 59.7, 48.4, 47.9, 45.3, 38.8, 29.4, 28.6, 14.3 ppm.

IR (ATR, neat): $\tilde{\nu}$ = 2942 (*w*), 2830 (*w*), 1715 (*m*), 1586 (*w*), 1299 (*m*), 1258 (*m*), 1216 (*m*), 1104 (*s*), 1058 (*s*), 1020 (*m*), 903 (*m*), 854 (*m*), 807 (*m*), 754 (*s*), 744 (*s*) cm^{-1}.

HRMS (ESI): calcd. for $C_{13}H_{21}NNaO_6$ $[M+Na]^+$: 310.1261; found: 310.1255.

Synthesis of ester 12

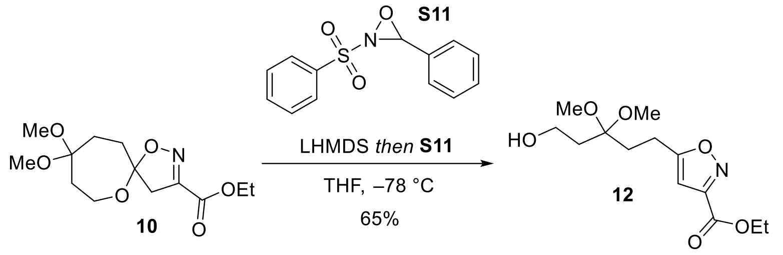

To a stirred solution of ester **10** (37 mg, 0.13 mmol, 1 equiv) in tetrahydrofuran (1 mL) was added dropwise a solution of lithium bis(trimethylsilyl)amide (1.0 M in tetrahydrofuran, 0.13 mL, 0.13 mmol, 1.0 equiv) at –78 °C and the resulting solution was stirred at that temperature for 15 min. A solution of 2-(phenylsulfonyl)-3-phenyl-1,2-oxaziridine (**S11**) (0.26 M in tetrahydrofuran, 0.50 mL, 0.13 mmol, 1.0 equiv) was added and the resulting solution was stirred at –78 °C for 2 h. A saturated aqueous sodium dihydrogen phosphate solution (0.5 mL) was added at –78 °C and the mixture was allowed to warm up to 23 °C. Diethyl ether (5 mL) and water (5 mL) were added. The organic layer was separated and washed with a saturated aqueous sodium chloride solution (5 mL). The washed solution was dried over magnesium sulfate and filtered. The dried filtrate was concentrated under reduced pressure and the residue was purified by flash chromatography on silica gel (50% ethyl acetate in cyclohexane) which afforded the ester **12** as a colorless oil (24 mg, 84 μmol, 65%).

TLC (50% ethyl acetate in cyclohexane): R_f = 0.25 (UV, $KMnO_4$).

^{1}H NMR (400 MHz, $CDCl_3$, 25 °C): δ 6.43 (s, 1H), 4.42 (q, J = 7.2 Hz, 2H), 3.77 – 3.69 (br m, 2H), 3.22 (s, 6H), 2.87 – 2.79 (m, 2H), 2.41 – 2.30 (br m, 1H), 2.12 – 2.05 (m, 2H), 1.95 (t, J = 6.0 Hz, 2H), 1.40 (t, J = 7.2 Hz, 3H) ppm.

^{13}C NMR (101 MHz, $CDCl_3$, 25 °C): δ 174.8, 160.3, 156.6, 102.6, 101.7, 62.3, 58.7, 48.2, 34.5, 31.3, 22.0, 14.3 ppm.

IR (ATR, neat): $\tilde{\nu}$ = 3456 (*w, br*), 2958 (*w*), 2832 (*w*), 1731 (*s*), 1594 (*w*), 1461 (*m*), 1246 (*m*), 1214 (*s*), 1112 (*s*), 1026 (*s*), 930 (*w*), 890 (*w*), 837 (*w*), 779 (*m*) cm^{-1}.

HRMS (ESI): calcd. for $C_{13}H_{21}NNaO_6$ $[M+Na]^+$: 310.1261; found: 310.1252.

Synthesis of ketone 17

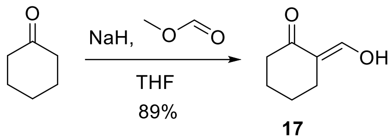

To a stirred suspension of sodium hydride (9.00 g, 60 wt% dispersion in mineral oil, 0.224 mol, 2.20 equiv) in tetrahydrofuran (240 mL) were added cyclohexanone (10.6 mL, 0.102 mol, 1 equiv) and methyl formate (12.6 mL, 0.204 mol, 2.00 equiv) at 23 °C and the resulting suspension was stirred for 20 h. A 2 M aqueous hydrogen chloride solution (120 mL) was added and the mixture was extracted with diethyl ether (2 × 200 mL). The combined organic solutions were dried over sodium sulfate and filtered. The dried filtrate was concentrated under reduced pressure and the residue was purified by flash chromatography on silica gel (7% diethyl ether in pentane) which afforded the ketone **17** as a pink oil (11.4 g, 90.4 mmol, 89%).

^{1}H NMR (400 MHz, $CDCl_3$, 25 °C): δ 14.41 (d, J = 2.7 Hz, 1H), 8.64 (d, J = 1.9 Hz, 1H), 2.40 – 2.31 (m, 4H), 1.75 – 1.63 (m, 4H). The analytical data matched those published before.[3]

Synthesis of nitronate 16a

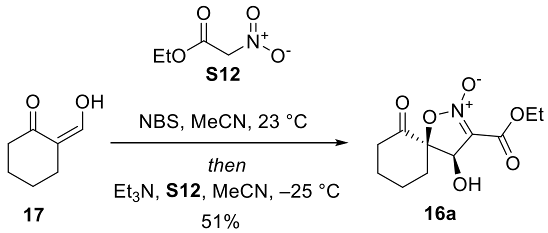

To a stirred solution of ketone **17** (11.4 g, 90.4 mmol, 1 equiv) in acetonitrile (350 mL) was added *N*-bromosuccinimide (16.1 g, 90.5 mmol, 1.00 equiv) at 23 °C and the resulting mixture was stirred for 5 min before being cooled to –25 °C. Ethyl nitroacetate (**S12**) (10.2 mL, 91.9 mmol, 1.02 equiv) was added dropwise over 1 min and triethylamine (13.2 mL, 94.7 mmol, 1.05 equiv) was added dropwise over 5 min. The resulting mixture was stirred at –25 °C for 4 h and then allowed to warm up to 23 °C over 1 h. Acetonitrile was removed under reduced pressure. To the residue was added water (200 mL) and the

mixture was extracted with ethyl acetate (4 × 200 mL). The combined organic solutions were dried over sodium sulfate and filtered. The dried filtrate was concentrated under reduced pressure and the residue was purified by flash chromatography on silica gel (50% diethyl ether in pentane) which afforded nitronate **16a** as a colorless solid (11.8 g, 45.8 mmol, 51%).

Crystals of nitronate **16a** for single crystal X-ray crystallography were prepared as follows: 10 mg of nitronate **16a** in a 4 mL vial were dissolved in 1 mL of diethyl ether and the vial was put into a 20 mL vial containing 5 mL of pentane. The larger vial was sealed with a cap and was left to stand at 23 °C for 24 h after which crystals were found inside the inner vial.

Characterization of intermediate aldehyde **S13**:

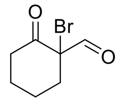

S13

Analytical sample was obtained as follows: An aliquot of the bromination reaction mixture (1 mL) was washed with water (1 mL), the organic solution was dried over sodium sulfate and filtered. The dried filtrate was concentrated in vacuo and the residue was purified by flash chromatography on silica gel (15% diethyl ether in pentane) which afforded the product as a pale-yellow oil that was immediately characterized.

TLC (20% ethyl acetate in cyclohexane): R_f = 0.42 (weak UV, $KMnO_4$).

^{1}H NMR (400 MHz, $CDCl_3$, 25 °C): δ 9.70 (s, 1H), 3.20 – 3.10 (m, 1H), 2.44 – 2.22 (m, 3H), 2.12 – 1.97 (m, 2H), 1.95 – 1.85 (m, 1H), 1.81 – 1.68 (m, 1H) ppm.

^{13}C NMR (101 MHz, $CDCl_3$, 25 °C): δ 203.6, 192.1, 67.1, 38.2, 34.9, 26.7, 21.4 ppm.

IR (ATR, neat): $\tilde{\nu}$ = 2946 (*w*), 2867 (*w*), 1727 (*s*), 1704 (*s*), 1449 (*w*), 1431 (*w*), 1338 (*w*), 1314 (*w*), 1271 (*w*), 1226 (*w*), 1164 (*m*), 1123 (*w*), 1074 (*w*), 1027 (*w*), 982 (*w*), 948 (*w*), 903 (*w*), 855 (*w*) cm^{-1}.

HRMS (ESI): calcd. for $C_8H_{13}BrNaO_3$ $[M+CH_3OH+Na]^+$: 258.9940; found: 258.9934.

Characterization of nitronate **16a**:

TLC (50% ethyl acetate in cyclohexane): R_f = 0.40 (UV, $KMnO_4$).

Melting point: 103 – 104 °C.

^{1}H NMR (400 MHz, $CDCl_3$, 25 °C): δ 5.75 (d, J = 4.2 Hz, 1H), 4.35 (q, J = 7.1 Hz, 2H), 2.90 – 2.73 (m, 2H), 2.57 – 2.48 (m, 1H), 2.38 – 2.29 (m, 1H), 2.14 – 1.73 (m, 5H), 1.36 (t, J = 7.1 Hz, 3H) ppm.

^{13}C NMR (101 MHz, $CDCl_3$, 25 °C): δ 203.7, 159.0, 110.9, 87.2, 72.4, 62.4, 39.1, 31.3, 26.9, 21.5, 14.3 ppm.

IR (ATR, neat): $\tilde{\nu}$ = 3472 (*m, br*), 2953 (*w*), 1687 (*m*), 1617 (*s*), 1422 (*m*), 1390 (*m*), 1249 (*m*), 1151 (*m*), 1017 (*m*), 918 (*m*), 768 (*m*) cm^{-1}.

HRMS (ESI): calcd. for $C_{11}H_{15}NNaO_6$ [M+Na]$^+$: 280.0792; found: 280.0788.

Characterization of nitronate **16b**:

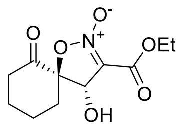

16b

TLC (50% ethyl acetate in cyclohexane): R_f = 0.35 (UV, $KMnO_4$).

Melting point: Decomposition at 169 °C.

^{1}H NMR (400 MHz, DMSO-d_6, 25 °C): δ 6.66 (d, J = 7.7 Hz, 1H), 5.42 (d, J = 7.7 Hz, 1H), 4.23 (q, J = 7.1 Hz, 2H), 2.58 (td, J = 14.1, 6.0 Hz, 1H), 2.44 – 2.35 (m, 1H), 2.34 – 2.26 (m, 1H), 2.04 – 1.69 (m, 4H), 1.67 – 1.51 (m, 1H), 1.25 (t, J = 7.1 Hz, 3H) ppm.

^{13}C NMR (101 MHz, DMSO-d_6, 25 °C): δ 201.2, 158.1, 110.0, 89.7, 75.3, 61.2, 41.8, 34.3, 25.9, 21.4, 14.0 ppm.

IR (ATR, neat): $\tilde{\nu}$ = 3482 (*m, br*), 1732 (*w*), 1685 (*m*), 1618 (s), 1426 (*m*), 1386 (*m*), 1267 (*m*), 1234 (*m*), 1161 (*m*), 1109 (*m*), 1041 (*s*), 930 (*m*), 879 (*m*), 852 (*m*), 755 (*m*), 737 (*m*) cm^{-1}.

HRMS (ESI): calcd. for $C_{11}H_{15}NNaO_6$ [M+Na]$^+$: 280.0792; found: 280.0788.

Screening of conditions for the synthesis of nitronate 16a

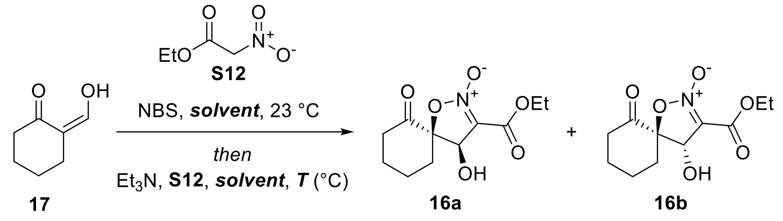

entry	*solvent*	*temperature (°C)*	*ratio (16a : 16b)*[a]
1	pentane	23	1.7 : 1
2	benzene	23	1.5 : 1
3	diethyl ether	23	1.6 : 1
4	dichloromethane	23	1.6 : 1
5	acetonitrile	23	1.9 : 1
6	1,1,1,3,3,3-hexafluoropropan-2-ol	23	1.8 : 1
7	methanol	23	1.2 : 1
8	water	23	1.1 : 1
9	acetonitrile	50	1.5 : 1
10	acetonitrile	0	2.2 : 1
11	acetonitrile	–25	3.3 : 1

[a] The ratios were calculated from crude ^{1}H NMR spectra

Synthesis of ester 18

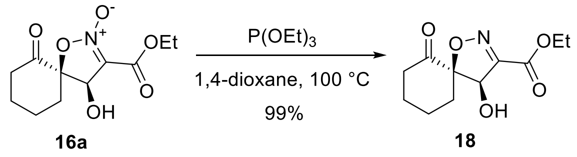

To a stirred solution of nitronate **16a** (6.85 g, 26.6 mmol, 1 equiv) in 1,4-dioxane (20 mL) was added triethylphosphite (7.08 mL, 41.3 mmol, 1.55 equiv) at 100 °C and the resulting solution was stirred at that temperature for 22 h. The solution was allowed to cool to 23 °C and was concentrated under reduced pressure. The residue was purified by flash chromatography on silica gel (35% diethyl ether in pentane grading to 50% diethyl ether in pentane) which afforded the ester **18** as a colorless oil (6.36 g, 26.4 mmol, 99%).

TLC (50% ethyl acetate in cyclohexane): R_f = 0.48 (UV, $KMnO_4$).

¹H NMR (400 MHz, $CDCl_3$, 25 °C): δ 5.72 (d, *J* = 4.7 Hz, 1H), 4.37 (q, *J* = 7.2 Hz, 2H), 2.93 – 2.81 (m, 2H), 2.54 – 2.44 (m, 1H), 2.36 – 2.28 (m, 1H), 2.13 – 1.71 (m, 5H), 1.37 (t, *J* = 7.1 Hz, 3H) ppm.

¹³C NMR (101 MHz, $CDCl_3$, 25 °C): *δ* 203.6, 160.7, 152.8, 94.7, 73.6, 62.6, 39.3, 30.9, 26.9, 21.3, 14.2 ppm.

IR (ATR, neat): $\tilde{\nu}$ = 3421 (*w, br*), 2946 (*w*), 2870 (*w*), 1715 (*s*), 1589 (*w*), 1377 (*w*), 1230 (*m*), 1126 (*m*), 1072 (*m*), 1036 (*m*), 915 (*m*), 829 (*m*), 730 (*m*) cm^{-1}.

HRMS (ESI): calcd. for $C_{11}H_{16}NO_5$ $[M+H]^+$: 242.1023; found: 242.1020.

Synthesis of lactone 19

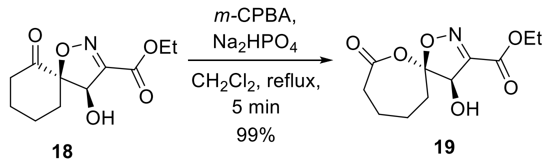

To a solution of ester **18** (170 mg, 0.705 mmol, 1 equiv) in dichloromethane (2.5 mL) were added disodium hydrogen phosphate (150 mg, 1.06 mmol, 1.50 equiv) in one portion and 3-chloroperoxybenzoic acid (164 mg, 77 wt%, 0.732 mmol, 1.04 equiv) in several portions over 5 min upon which the mixture started spontaneously refluxing. After cooling to 23 °C, the mixture was poured into a saturated aqueous sodium bicarbonate solution (10 mL) and a saturated aqueous sodium thiosulfate solution (10 mL) was added. The mixture was extracted with dichloromethane (20 mL). The organic solution was dried over sodium sulfate and filtered. The dried filtrate was concentrated under reduced pressure which afforded the lactone **19** as a colorless oil (180 mg, 0.697 mmol, 99%) which did not require further purification.

TLC (50% ethyl acetate in cyclohexane): R_f = 0.44 (UV, $KMnO_4$).

¹H NMR (400 MHz, $CDCl_3$, 25 °C): *δ* 5.10 (d, *J* = 4.5 Hz, 1H), 4.45 – 4.35 (m, 2H), 3.15 (br s, 1H), 3.04 (td, *J* = 14.0, 2.2 Hz, 1H), 2.81 – 2.71 (m, 1H), 2.63 – 2.54 (m, 1H), 2.05 – 1.84 (m, 4H), 1.73 – 1.60 (m, 1H), 1.39 (t, *J* = 7.1 Hz, 3H) ppm.

¹³C NMR (101 MHz, $CDCl_3$, 25 °C): *δ* 173.0, 160.0, 154.7, 111.4, 81.8, 63.0, 36.2, 29.1, 23.3, 23.1, 14.2 ppm.

IR (ATR, neat): $\tilde{\nu}$ = 3412 (*m*, *br*), 2941 (*w*), 1725 (*s*), 1599 (*w*), 1436 (*w*), 1377 (*m*), 1348 (*m*), 1333 (*m*), 1289 (*m*), 1238 (*m*), 1160 (*m*), 1113 (*m*), 1075 (*m*), 1062 (*m*), 1005 (*m*), 987 (*m*), 919 (*m*), 887 (*m*), 855 (*m*), 801 (w), 769 (w) cm^{-1}.

HRMS (ESI): calcd. for $C_{11}H_{16}NO_6$ $[M+H]^+$: 258.0972; found: 258.0967.

Synthesis of carbonate 20

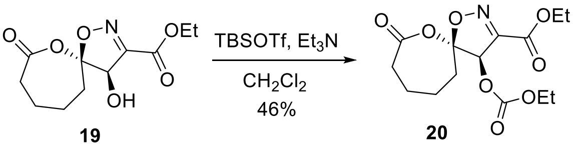

To a stirred solution of lactone **19** (170 mg, 0.658 mmol, 1 equiv) in dichloromethane (2.5 mL) were added triethylamine (0.110 mL, 0.789 mmol, 1.20 equiv) and *tert*-butyldimethylsilyl trifluoromethanesulfonate (0.170 mL, 0.727 mmol, 1.10 equiv) and the resulting solution was stirred for 30 min at 23 °C. A saturated aqueous sodium bicarbonate solution (5 mL) was added and the mixture was extracted with ethyl acetate (5 mL). The organic solution was dried over sodium sulfate and filtered. The dried filtrate was concentrated under reduced pressure and the residue was purified by flash chromatography on silica gel (30% diethyl ether in pentane) which afforded the carbonate **20** as a colorless oil (100 mg, 0.303 mmol, 46%).

TLC (50% ethyl acetate in cyclohexane): R_f = 0.65 (UV, $KMnO_4$).

^{1}H NMR (400 MHz, $CDCl_3$, 25 °C): δ 6.10 (s, 1H), 4.43 – 4.33 (m, 2H), 4.32 – 4.26 (m, 2H), 3.06 – 2.96 (m, 1H), 2.84 – 2.75 (m, 1H), 2.38 – 2.30 (m, 1H), 2.04 – 1.89 (m, 4H), 1.71 – 1.62 (m, 1H), 1.39 – 1.31 (m, 6H) ppm.

^{13}C NMR (101 MHz, $CDCl_3$, 25 °C): δ 171.9, 158.4, 153.2, 151.9, 110.4, 83.3, 65.9, 62.9, 36.2, 29.1, 23.3, 22.8, 14.2, 14.1 ppm.

IR (ATR, neat): $\tilde{\nu}$ = 2941 (*w*), 1762 (*s*), 1449 (*w*), 1373 (*w*), 1336 (*w*), 1291 (*w*), 1253 (*s*), 1157 (*m*), 1121 (*w*), 1079 (*w*), 1020 (*m*), 980 (*w*), 914 (*w*), 874 (*w*), 853 (*w*) cm^{-1}.

HRMS (ESI): calcd. for $C_{14}H_{20}NO_8$ $[M+H]^+$: 330.1183; found: 330.1180.

Synthesis of ester 21

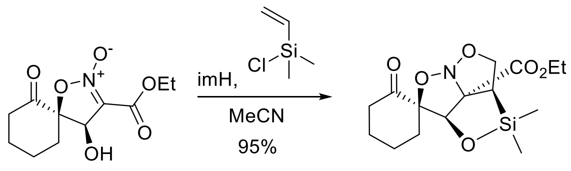

To a solution of nitronate **16a** (1.26 g, 4.90 mmol, 1 equiv) in acetonitrile (10 mL) were added chlorodimethylvinylsilane (740 mg, 6.12 mmol, 1.25 equiv) and imidazole (417 mg, 6.12 mmol, 1.25 equiv) and the resulting suspension was stirred for 20 h. Acetonitrile was removed under reduced pressure. To the residue was added a saturated aqueous sodium bicarbonate solution (20 mL) and the mixture was extracted with ethyl acetate (20 mL). The organic solution was washed with a saturated aqueous sodium chloride solution (20 mL). The washed solution was dried over sodium sulfate and filtered. The dried filtrate was concentrated under reduced pressure which afforded the ester **21** as white solid (1.59 g, 4.66 mmol, 95%) which did not require further purification.

TLC (20% ethyl acetate in cyclohexane): R_f = 0.17 (UV, $KMnO_4$).

Melting point: 96 – 103 °C.

^{1}H NMR (400 MHz, $CDCl_3$, 25 °C): δ 5.35 (s, 1H), 4.46 (dd, J = 9.9, 8.2 Hz, 1H), 4.37 (dd, J = 11.4, 8.2 Hz, 1H), 4.33 – 4.22 (m, 2H), 3.13 (td, J = 13.3, 6.0 Hz, 1H), 2.31 – 2.21 (m, 2H), 2.18 – 2.03 (m, 2H), 1.90 – 1.76 (m, 1H), 1.73 – 1.52 (m, 3H), 1.32 (t, J = 7.2 Hz, 3H), 0.39 (s, 3H), 0.37 (s, 3H) ppm.

^{13}C NMR (101 MHz, $CDCl_3$, 25 °C): δ 210.6, 170.3, 94.8, 85.7, 84.4, 74.0, 62.2, 38.1, 38.0, 31.9, 27.9, 21.4, 14.2, –0.1, –2.9 ppm.

IR (ATR, neat): $\tilde{\nu}$ = 2944 (*w*), 1752 (*m*), 1719 (*s*), 1448 (*w*), 1436 (*w*), 1314 (*w*), 1283 (*w*), 1250 (*s*), 1210 (*w*), 1186 (*w*), 1150 (*w*), 1116 (*m*), 1101 (*m*), 1074 (*m*), 1041 (*m*), 986 (*w*), 906 (*w*), 879 (*m*), 847 (*m*), 802 (*m*), 780 (*w*) cm^{-1}.

HRMS (ESI): calcd. for $C_{15}H_{23}NNaO_6Si$ $[M+Na]^+$: 364.1187; found: 364.1177.

Synthesis of lactone 22

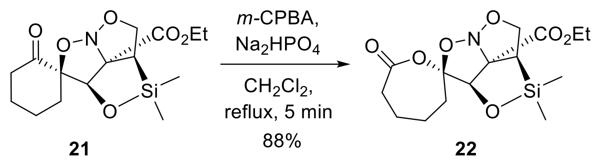

To a stirred solution of ester **21** (1.59 g, 4.66 mmol, 1 equiv) in dichloromethane (5 mL) was added solid disodium hydrogen phosphate (993 mg, 6.99 mmol, 1.50 equiv) in one portion and 3-chloroperoxybenzoic acid (1.09 g, 77 wt%, 4.86 mmol, 1.04 equiv) in several portions over 5 min upon which the mixture heated to reflux spontaneously. After cooling to 23 °C, a saturated aqueous sodium thiosulfate solution (10 mL) and a saturated aqueous sodium bicarbonate solution (10 mL) were added. The mixture was extracted with dichloromethane (20 mL), the organic layer was dried over sodium sulfate and filtered. The dried filtrate was concentrated under reduced pressure which afforded the lactone **22** as a colorless oil (1.47 g, 4.11 mmol, 88%) which did not require further purification.

TLC (50% ethyl acetate in cyclohexane): R_f = 0.46 (UV, $KMnO_4$).

^{1}H NMR (400 MHz, $CDCl_3$, 25 °C): δ 4.93 (s, 1H), 4.52 (dd, J = 9.6, 8.5 Hz, 1H), 4.38 – 4.19 (m, 3H), 3.10 (td, J = 13.6, 2.3 Hz, 1H), 2.71 – 2.63 (m, 1H), 2.48 – 2.39 (m, 1H), 2.26 (dd, J = 12.0, 9.6 Hz, 1H), 1.94 – 1.68 (m, 4H), 1.61 – 1.50 (m, 1H), 1.32 (t, J = 7.2 Hz, 3H), 0.40 (s, 3H), 0.37 (s, 3H) ppm.

^{13}C NMR (101 MHz, $CDCl_3$, 25 °C): δ 173.4, 169.8, 107.4, 93.7, 90.3, 74.3, 62.7, 38.1, 36.4, 30.2, 23.4, 23.3, 14.1, –0.3, –2.9 ppm.

IR (ATR, neat): $\tilde{\nu}$ = 2942 (*w*), 1734 (*s*), 1369 (*m*), 1314 (*m*), 1288 (*m*), 1254 (*m*), 1194 (*m*), 1166 (*m*), 1134 (*m*), 1099 (*m*), 1066 (*m*), 1041 (*m*), 986 (*m*), 898 (*m*), 881 (*m*), 867 (*m*), 839 (*m*) cm^{-1}.

HRMS (ESI): calcd. for $C_{15}H_{24}NO_7Si$ $[M+H]^+$: 358.1317; found: 358.1310.

Synthesis of ester S14 and lactone 15

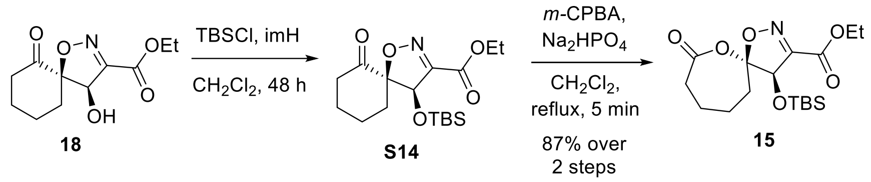

To a solution of ester **18** (4.17 g, 17.3 mmol, 1 equiv) in dichloromethane (25 mL) were added imidazole (1.47 g, 21.6 mmol, 1.25) and *tert*-butyldimethylsilyl chloride (3.26 g, 21.6 mmol, 1.25 equiv) and the resulting suspension was stirred at 23 °C for 48 h. A saturated aqueous sodium bicarbonate solution (50 mL) was added and the mixture was extracted with dichloromethane (2 × 50 mL). The organic solution was dried over sodium sulfate and filtered. The dried filtrate was concentrated under reduced pressure which afforded the crude ester **S14** as a colorless oil which was dissolved in dichloromethane (50 mL). To that stirred solution were added disodium hydrogen phosphate (3.68 g, 25.9 mmol, 1.50 equiv) in one portion and 3-chloroperoxybenzoic acid (4.03 g, 77 wt%, 18.0 mmol, 1.04 quiv) in several portions over 5 min which caused the reaction mixture to spontaneously reflux. The resulting suspension was stirred for 5 min during which it cooled to 23 °C. A saturated aqueous sodium bicarbonate solution (60 mL) and a saturated aqueous sodium thiosulfate solution (40 mL) were added and the mixture was vigorously stirred until all solids had dissolved. The mixture was extracted with dichloromethane (2 × 100 mL), the organic solution was dried over sodium sulfate and filtered. The dried filtrate was concentrated under reduced pressure and the residue was purified by flash chromatography on silica gel (10% diethyl ether in pentane grading to 20% diethyl ether in pentane) which afforded the lactone **15** as a colorless solid (5.57 g, 15.0 mmol, 87% over 2 steps).

Characterization of ester **S14**:

Analytical sample was obtained by flash chromatography on silica gel (10% diethyl ether in pentane).

TLC (20% ethyl acetate in cyclohexane): R_f = 0.64 (UV, $KMnO_4$).

^{1}H NMR (400 MHz, $CDCl_3$, 25 °C): δ 5.77 (s, 1H), 4.43 – 4.25 (m, 2H), 2.93 (td, *J* = 13.4, 6.1 Hz, 1H), 2.48 – 2.40 (m, 1H), 2.39 – 2.30 (m, 1H), 2.18 – 1.96 (m, 2H), 1.88 – 1.78 (m, 1H), 1.75 – 1.61 (m, 2H), 1.36 (t, *J* = 7.2 Hz, 3H), 0.86 (s, 9H), 0.18 (s, 3H), 0.12 (s, 3H) ppm.

^{13}C NMR (101 MHz, $CDCl_3$, 25 °C): δ 204.4, 160.0, 153.7, 94.8, 74.2, 62.2, 39.4, 32.1, 27.2, 25.8, 21.3, 18.3, 14.3, –4.6, –4.8 ppm.

IR (ATR, neat): $\tilde{\nu}$ = 2933 (*w*), 2857 (*w*), 1720 (*s*), 1589 (*w*), 1327 (*w*), 1231 (*s*), 1107 (*m*), 1087 (*s*), 1053 (*m*), 1022 (*m*), 953 (*m*), 933 (*m*), 838 (*s*), 777 (*s*) cm^{-1}.

HRMS (ESI): calcd. for $C_{17}H_{30}NO_5Si$ $[M+H]^+$: 356.1888; found: 356.1883.

Characterization of lactone **15**:

TLC (20% ethyl acetate in cyclohexane): R_f = 0.53 (UV, $KMnO_4$).

Melting point: 69 – 72 °C.

^{1}H NMR (400 MHz, $CDCl_3$, 25 °C): δ 5.05 (s, 1H), 4.46 – 4.28 (m, 2H), 3.10 – 2.99 (m, 1H), 2.79 – 2.70 (m, 1H), 2.54 – 2.45 (m, 1H), 2.06 – 1.93 (m, 3H), 1.82 – 1.59 (m, 2H), 1.38 (t, *J* = 7.1 Hz, 3H), 0.88 (s, 9H), 0.19 – 0.16 (m, 6H) ppm.

^{13}C NMR (101 MHz, $CDCl_3$, 25 °C): δ 173.0, 159.4, 155.3, 111.4, 82.8, 62.6, 36.2, 29.9, 25.7, 23.5, 23.1, 18.2, 14.2, –4.4, –4.9 ppm.

IR (ATR, neat): $\tilde{\nu}$ = 2934 (*w*), 2861 (*w*), 1746 (*m*), 1721 (*m*), 1236 (*m*), 1143 (*s*), 1113 (*s*), 1070 (*m*), 1003 (*m*), 906 (*m*), 864 (*m*), 835 (*s*), 784 (*s*), 726 (*m*) cm^{-1}.

HRMS (ESI): calcd. for $C_{17}H_{30}NO_6Si$ $[M+H]^+$: 372.1837; found: 372.1835.

Synthesis of ketene acetal phosphate 24

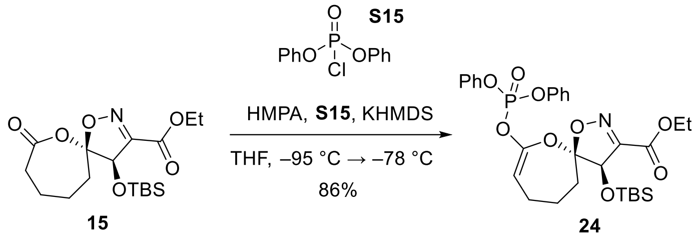

To a stirred solution of lactone **15** (1.00 g, 2.69 mmol, 1 equiv) in tetrahydrofuran (10 mL) cooled to −95 °C was added hexamethylphosphoramide (0.560 mL, 3.23 mmol, 1.20 equiv) followed by diphenyl chlorophosphate (**S15**) (0.670 mL, 3.23 mmol, 1.20 equiv) which solidified upon addition. The mixture was allowed to warm up until a homogeneous solution was obtained which was then cooled back to −95 °C. A solution of potassium bis(trimethylsilyl)amide (1.00 M in tetrahydrofuran, 3.00 mL, 3.00 mmol, 1.11 equiv) was added dropwise over 5 min and the resulting solution was allowed to warm up to −78 °C. After stirring for 1 h, a solution of sodium dihydrogen phosphate (2.69 M in water, 10.0 mL, 26.9 mmol, 10.0 equiv) was added at −78 °C and the mixture was allowed to warm up to 23 °C. The mixture was extracted with ethyl acetate (2 × 20 mL). The organic solution was washed with a saturated aqueous sodium chloride solution (20 mL). The washed solution was dried over sodium sulfate and filtered. The dried filtrate was concentrated under reduced pressure and the residue was purified by flash chromatography on silica gel (20% diethyl ether in pentane grading to 30% diethyl ether in pentane) which afforded the ketene acetal phosphate **24** as a colorless oil (1.40 g, 2.32 mmol, 86%).

TLC (20% ethyl acetate in cyclohexane): R_f = 0.39 (UV, $KMnO_4$).

^{1}H NMR (400 MHz, $CDCl_3$, 25 °C): δ 7.38 – 7.28 (m, 4H), 7.24 – 7.14 (m, 6H), 5.04 – 4.98 (m, 1H), 4.88 (s, 1H), 4.36 – 4.25 (m, 1H), 4.23 – 4.14 (m, 1H), 2.32 – 1.95 (m, 4H), 1.85 – 1.72 (m, 2H), 1.30 (t, J = 7.1 Hz, 3H), 0.86 (s, 9H), 0.17 (s, 3H), 0.13 (s, 3H) ppm.

^{13}C NMR (101 MHz, $CDCl_3$, 25 °C): δ 159.70, 155.11, 150.57 (d, J_{C-P} = 7.4 Hz), 148.47 (d, J_{C-P} = 7.8 Hz), 129.86 (d, J_{C-P} = 6.5 Hz), 125.61 (d, J_{C-P} = 1.2 Hz), 125.56 (d, J_{C-P} = 1.2 Hz), 120.29 (d, J_{C-P} = 1.9 Hz), 120.24 (d, J_{C-P} = 1.9 Hz), 112.05, 96.44 (d, J_{C-P} = 5.6 Hz), 81.27, 62.32, 30.77, 25.76, 23.73, 19.72, 18.22, 14.16, −4.53, −4.77 ppm.

^{31}P NMR (162 MHz, $CDCl_3$, 25 °C): δ −18.3 ppm.

IR (ATR, neat): $\tilde{\nu}$ = 2931 (*w*), 2858 (*w*), 1725 (*m*), 1698 (*w*), 1591 (*w*), 1489 (*m*), 1379 (*m*), 1300 (*m*), 1255 (*m*), 1233 (*m*), 1119 (*s*), 1009 (*m*), 944 (*s*), 900 (*m*), 837 (*s*), 775 (*s*), 687 (*m*) cm^{-1}.

HRMS (ESI): calcd. for $C_{29}H_{39}NO_9PSi$ $[M+H]^+$: 604.2126; found: 604.2121.

Synthesis of enol ether 25

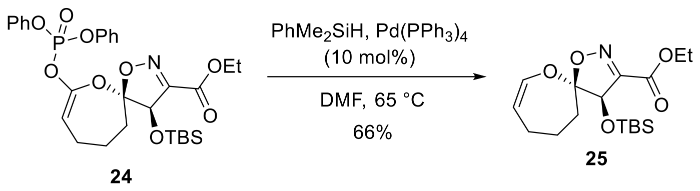

To a stirred solution of ketene acetal phosphate **24** (1.40 g, 2.32 mmol, 1 equiv) in *N*,*N*-dimethylformamide (2 mL) was added a suspension of tetrakis(triphenylphosphine)-palladium (268 mg, 0.232 mmol, 10 mol%) in *N*,*N*-dimethylformamide (8 mL) at 65 °C followed by dimethylphenylsilane (1.78 mL, 11.6 mmol, 5.00 equiv). The resulting solution was stirred at 65 °C for 2 h. The solution was allowed to cool to 23 °C and ethyl acetate (25 mL) was added. The resulting solution was washed with 5% aqueous lithium chloride solution (2 × 25 mL) and a saturated aqueous sodium chloride (25 mL). The washed solution was dried over sodium sulfate and filtered. The dried filtrate was concentrated under reduced pressure and the residue was purified by flash chromatography on silica gel (0% diethyl ether in petroleum ether grading to 5% diethyl ether in petroleum ether) which afforded the enol ether **25** as a colorless oil (544 mg, 1.53 mmol, 66%).

TLC (5% ethyl acetate in cyclohexane): R_f = 0.45 (UV, $KMnO_4$).

^{1}H NMR (400 MHz, $CDCl_3$, 25 °C): δ 5.91 – 5.86 (m, 1H), 5.05 (td, *J* = 6.7, 4.5 Hz, 1H), 4.84 (s, 1H), 4.46 – 4.27 (m, 2H), 2.37 – 2.29 (m, 1H), 2.28 – 2.16 (m, 2H), 2.04 – 1.95 (m, 1H), 1.88 – 1.74 (m, 2H), 1.38 (t, *J* = 7.1 Hz, 3H), 0.88 (s, 9H), 0.19 (s, 3H), 0.15 (s, 3H) ppm.

^{13}C NMR (101 MHz, $CDCl_3$, 25 °C): δ 160.3, 155.3, 140.8, 114.3, 113.0, 80.9, 62.3, 31.0, 27.2, 25.8, 20.2, 18.3, 14.3, –4.6, –4.7 ppm.

IR (ATR, neat): $\tilde{\nu}$ = 2932 (*m*), 2859 (*w*), 1724 (*m*), 1657 (*w*), 1589 (*w*), 1472 (*w*), 1380 (*w*), 1231 (*m*), 1198 (*m*), 1177 (*w*), 1116 (*s*), 1073 (*w*), 1010 (*w*), 901 (*m*), 841 (*s*), 781 (*m*), 738 (*w*) cm^{-1}.

HRMS (ESI): calcd. for $C_{17}H_{30}NO_5Si$ $[M+H]^+$: 356.1888; found: 356.1876.

Synthesis of vinylogous lactone 26 and lactone 27

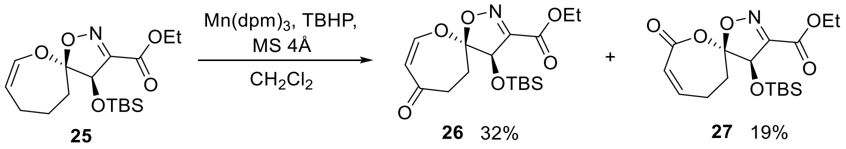

The reaction flask was charged with enol ether **25** (200 mg, 0.563 mmol, 1 equiv), tris(dipivaloylmethanato)manganese(III) (68.0 mg, 0.113 mmol, 20 mol%), molecular sieves (4Å, 550 mg), and dichloromethane (2.5 mL). To the stirred mixture was added a solution of *tert*-butyl hydroperoxide (5.50 M in decane, 1.02 mL, 5.63 mmol, 10.0 equiv) via syringe pump over 24 h at 23 °C. Ethyl acetate (20 mL) was added and the mixture was filtered. The filtrate was washed with a saturated aqueous sodium thiosulfate solution (20 mL) and a saturated aqueous sodium chloride solution (20 mL). The washed solution was dried over sodium sulfate and filtered. The dried filtrate was concentrated under reduced pressure and the residue was purified by flash chromatography on silica gel (15% diethyl ether in pentane grading to 30% diethyl ether in pentane) which afforded lactone **27** as a colorless solid (40.1 mg, 0.109 mmol, 19%) and vinylogous lactone **26** as a colorless oil (66.2 mg, 0.179 mmol, 32%).

Characterization of vinylogous lactone **26**:

TLC (20% ethyl acetate in cyclohexane): R_f = 0.39 (UV, $KMnO_4$).

^{1}H NMR (400 MHz, $CDCl_3$, 25 °C): δ 6.41 (d, J = 8.5 Hz, 1H), 5.35 (dd, J = 8.5, 1.2 Hz, 1H), 5.04 (s, 1H), 4.47 – 4.30 (m, 2H), 3.05 – 2.94 (m, 1H), 2.78 – 2.69 (m, 1H), 2.58 – 2.50 (m, 1H), 2.22 – 2.13 (m, 1H), 1.39 (t, J = 7.2 Hz, 3H), 0.90 (s, 9H), 0.22 – 0.18 (m, 6H) ppm.

^{13}C NMR (101 MHz, $CDCl_3$, 25 °C): δ 199.0, 159.7, 155.3, 149.0, 113.3, 110.8, 81.6, 62.7, 36.9, 25.7, 22.6, 18.2, 14.2, –4.5, –4.9 ppm.

IR (ATR, neat): $\tilde{\nu}$ = 2956 (*w*), 2932 (*w*), 2859 (*w*), 1726 (*m*), 1660 (*m*), 1619 (*m*), 1471 (*w*), 1404 (*w*), 1379 (*w*), 1343 (*w*), 1305 (*m*), 1265 (*m*), 1236 (*m*), 1153 (*m*), 1103 (*s*), 1048 (*w*), 1010 (*w*), 985 (*w*), 899 (*m*), 840 (*s*), 783 (*m*) cm^{-1}.

HRMS (ESI): calcd. for $C_{17}H_{28}NO_6Si$ $[M+H]^+$: 370.1680; found: 370.1674.

Characterization of lactone **27**:

TLC (20% ethyl acetate in cyclohexane): R_f = 0.45 (UV, $KMnO_4$).

^{1}H NMR (400 MHz, $CDCl_3$, 25 °C): δ 6.60 – 6.52 (m, 1H), 6.08 – 6.01 (m, 1H), 5.13 (s, 1H), 4.45 – 4.29 (m, 2H), 2.88 – 2.75 (m, 1H), 2.74 – 2.61 (m, 2H), 2.21 – 2.11 (m, 1H), 1.37 (t, *J* = 7.2 Hz, 3H), 0.89 (s, 9H), 0.21 – 0.17 (m, 6H) ppm.

^{13}C NMR (101 MHz, $CDCl_3$, 25 °C): δ 164.7, 159.4, 155.1, 146.4, 122.0, 110.2, 82.4, 62.6, 27.4, 26.9, 25.8, 18.2, 14.3, –4.5, –4.9 ppm.

IR (ATR, neat): $\tilde{\nu}$ = 2956 (*w*), 2930 (*w*), 2858 (*w*), 1720 (*s*), 1465 (*w*), 1401 (*w*), 1379 (*w*), 1345 (*w*), 1285 (*m*), 1244 (*m*), 1176 (*m*), 1150 (*m*), 1087 (*m*), 1044 (*m*), 911 (*m*), 887 (*m*), 841 (*s*), 784 (*m*) cm^{-1}.

HRMS (ESI): calcd. for $C_{17}H_{28}NO_6Si$ $[M+H]^+$: 370.1680; found: 370.1675.

Converting lactone 27 into lactone 15

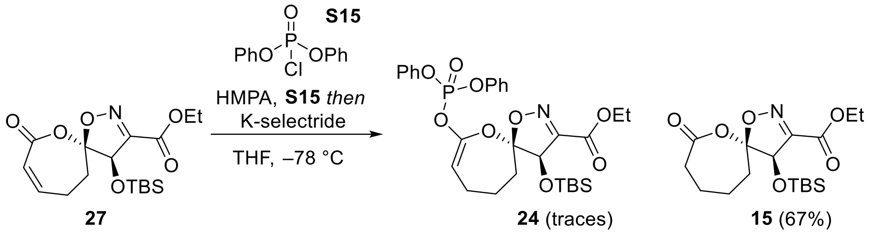

To a solution of lactone **27** (10 mg, 27 µmol, 1 equiv) in tetrahydrofuran (0.2 mL) cooled to –78 °C were added hexamethylphosphoramide (19 µL, 108 µmol, 4.0 equiv) and diphenyl chlorophosphate (**S15**) (23 µL, 108 µmol, 4.0 equiv) which solidified upon addition. The mixture was allowed to warm up until a homogeneous solution was obtained which was then cooled back to –78 °C. A solution of potassium tri-*sec*-butylborohydride (1.0 M in tetrahydrofuran, 30 µL, 30 µmol, 1.1 equiv) was added

dropwise and the resulting solution was stirred at –78 °C for 1 h. A saturated aqueous sodium bicarbonate solution (1 mL) was added and the resulting mixture was allowed to warm up to 23 °C. The mixture was extracted with ethyl acetate (2 × 1 mL). The organic solution was dried over sodium sulfate and filtered. The dried filtrate was concentrated in vacuo and the residue was purified by flash chromatography on silica gel (10% ethyl acetate in cyclohexane grading to 20% ethyl acetate in cyclohexane) which afforded the lactone **15** as a colorless oil (6.7 mg, 18 μmol, 67%).

Screening of conditions for the allylic oxidation of enol ether 25

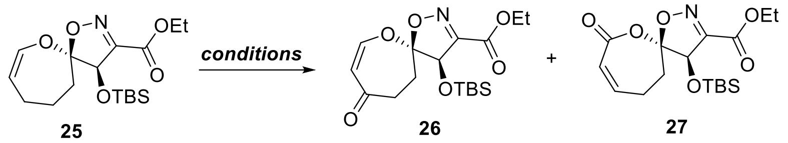

entry	*conditions*	*conversion (%)*[a]	*ratio (26 : 27)*[a]
1	CrO_3, 3,5-dimethylpyrazol, CH_2Cl_2, 0 °C	0	ND
2	SeO_2, TBHP, CH_2Cl_2, 40 °C	0	ND
3	PIDA, TBHP, K_2CO_3, EtOAc, 23 °C	50	1 : 1.3
4	$RuCl_3$, TBHP, CH_2Cl_2, 23 °C	65	1 : 6.0
5	$Pd(OH)_2$/C, TBHP, K_2CO_3, EtOAc, 23 °C	70	1 : 0.8
6	$Pd(OH)_2$/C, TBHP, butyl butyrate, 23 °C	50	1 : 0.4
7[b]	$Pd(OH)_2$/C, TBHP, butyl butyrate, 23 °C	100	1 : 0.7
8	CuBr, TBHP, CH_2Cl_2, 40 °C	100	1 : 2.0
9	CuBr, TBHP, EtOAc, 40 °C	65	1 : 2.2
10	CuBr, TBHP, MeCN, 40 °C	70	1 : 0.8
11	CuBr, TBHP, PhH, 50 °C	100	1 : 1.3
12	CuI, TBHP, PhH, 50 °C	80	1 : 1.6
13	CuI, TBHP, EtOAc, 50 °C	80	1 : 1.4
14	$Mn(OAc)_3$, TBHP, EtOAc, 23 °C	65	1 : 1.4
15[b]	$Mn(dpm)_3$, TBHP, CH_2Cl_2, 23 °C	100	1 : 0.7
16[b]	$Mn(dpm)_3$, TBHP, butyl butyrate, 23 °C	100	1 : 0.7

[a] The conversions and ratios were calculated from ^1H NMR spectra; [b] TBHP solution was added via syringe pump over 24 h

Synthesis of vinylogous lactone S16

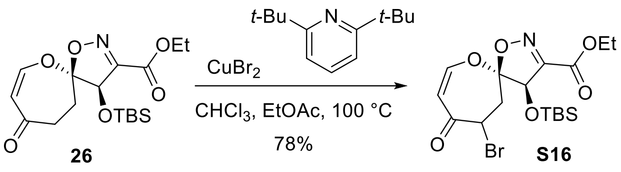

To a solution of vinylogous lactone **26** (84.0 mg, 0.228 mmol, 1 equiv) in chloroform (0.5 mL) and ethyl acetate (1.0 mL) were added 2,6-di-*tert*-butyl pyridine (0.123 mL, 0.569 mmol, 2.50 equiv) and copper(II) bromide (112 mg, 0.501 mmol, 2.20 equiv) and the resulting mixture was stirred and heated to 100 °C in a pressure tube for 5 h. The mixture was allowed to cool to 23 °C and water (10 mL) was added. The mixture was extracted with ethyl acetate (2 × 10 mL). The combined organic solutions were washed with a saturated aqueous sodium chloride solution (10 mL). The washed solution was dried over sodium sulfate and filtered. The dried filtrate was concentrated under reduced pressure and the residue was purified by flash chromatography on silica gel (10% diethyl ether in pentane grading to 20% diethyl ether in pentane) which afforded the vinylogous lactone **S16** as a colorless oil (79.2 mg, 0.177 mmol, 78%).

Note: The vinylogous lactone **S16** was obtained as a single diastereomer, however, it was not possible to determine the relative configuration of the α-bromoketone stereocenter introduced in this step.

TLC (20% ethyl acetate in cyclohexane): R_f = 0.43 (UV, $KMnO_4$).

^{1}H NMR (400 MHz, $CDCl_3$, 25 °C): δ 6.46 (d, J = 8.7 Hz, 1H), 5.45 (d, J = 8.6 Hz, 1H), 5.08 (s, 1H), 4.88 (dd, J = 12.5, 4.8 Hz, 1H), 4.48 – 4.29 (m, 2H), 3.20 (dd, J = 15.1, 4.8 Hz, 1H), 2.63 (dd, J = 15.1, 12.5 Hz, 1H), 1.38 (t, J = 7.2 Hz, 3H), 0.91 (s, 9H), 0.21 (s, 3H), 0.19 (s 3H) ppm.

^{13}C NMR (101 MHz, $CDCl_3$, 25 °C): δ 192.3, 159.3, 155.2, 148.5, 111.5, 107.3, 82.1, 62.9, 46.9, 34.0, 25.7, 18.2, 14.2, –4.6, –4.8 ppm.

IR (ATR, neat): $\tilde{\nu}$ = 2955 (*w*), 2932 (*w*), 2859 (*w*), 1727 (*m*), 1669 (*m*), 1616 (*m*), 1471 (*w*), 1466 (*w*), 1405 (*w*), 1379 (*w*), 1329 (*m*), 1253 (*s*), 1151 (*m*), 1102 (*s*), 1040 (*m*), 1008 (*w*), 989 (*w*), 900 (*m*), 835 (*s*), 805 (*m*), 783 (*s*), 746 (*w*) cm^{-1}.

HRMS (ESI): calcd. for $C_{17}H_{27}BrNO_6Si$ $[M+H]^+$: 448.0786; found: 448.0782.

Synthesis of vinylogous lactone 28

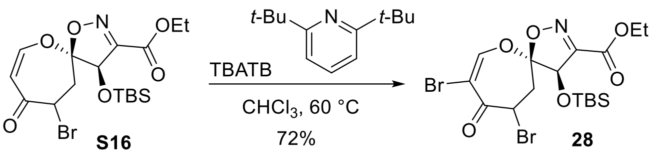

To a solution of vinylogous lactone **S16** (76 mg, 0.17 mmol, 1 equiv) in chloroform (0.5 mL) were added 2,6-di-*tert*-butylpyridine (55 μL, 0.25 mmol, 1.5 equiv) and tetra-*n*-butylammonium tribromide (96 mg, 0.20 mmol, 1.2 equiv) and the resulting solution was stirred at 60 °C for 8 h. The solution was allowed to cool to 23 °C and ethyl acetate (10 mL) was added. The solution was washed with a saturated aqueous sodium thiosulfate solution (10 mL) and a saturated aqueous sodium chloride solution (10 mL). The washed solution was dried over sodium sulfate and filtered. The dried filtrate was concentrated under reduced pressure and the residue was purified by flash chromatography on silica gel (10% diethyl ether in pentane grading to 15% diethyl ether in pentane) which afforded the vinylogous lactone **28** as a colorless oil (64 mg, 0.12 mmol, 72%).

Note: The vinylogous lactone **28** was obtained as a single diastereomer, however, it was not possible to determine the relative configuration of the α-bromoketone stereocenter.

TLC (20% ethyl acetate in cyclohexane): R_f = 0.55 (UV, $KMnO_4$).

^{1}H NMR (400 MHz, $CDCl_3$, 25 °C): δ 7.24 (s, 1H), 5.11 (s, 1H), 4.89 (dd, J = 10.9, 5.0 Hz, 1H), 4.48 – 4.30 (m, 2H), 3.12 (dd, J = 15.5, 5.0 Hz, 1H), 2.74 (dd, J = 15.5, 10.8 Hz, 1H), 1.39 (t, J = 7.2 Hz, 3H), 0.91 (s, 9H), 0.23 (s, 3H), 0.19 (s, 3H) ppm.

^{13}C NMR (101 MHz, $CDCl_3$, 25 °C): δ 186.5, 159.2, 155.5, 149.2, 112.1, 106.7, 81.8, 63.0, 44.4, 33.2, 25.8, 18.3, 14.2, –4.5, –4.8 ppm.

IR (ATR, neat): $\tilde{\nu}$ = 2956 (*w*), 2932 (*w*), 2859 (*w*), 1728 (*m*), 1686 (*w*), 1587 (*m*), 1471 (*w*), 1379 (*w*), 1345 (*w*), 1325 (*w*), 1256 (*m*), 1122 (*m*), 1064 (*w*), 1007 (*w*), 897 (*m*), 839 (*s*), 814 (*m*), 784 (*m*) cm^{-1}.

HRMS (ESI): calcd. for $C_{17}H_{25}Br_2NNaO_6Si$ $[M+Na]^+$: 547.9710; found: 547.9706.

Telescoped synthesis of vinylogous lactone 28 from enol ether 25

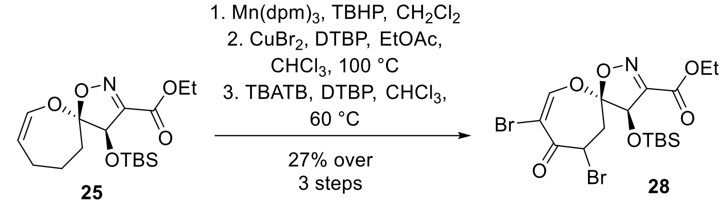

The reaction flask was charged with enol ether **25** (280 mg, 0.788 mmol), tris(dipivaloylmethanato)manganese(III) (95.3 mg, 0.158 mmol, 20 mol%), molecular sieves (4Å, 500 mg), and dry dichloromethane (2.5 mL). To the stirred mixture was added a solution of *tert*-butyl hydroperoxide (5.50 M in decane, 1.43 mL, 7.88 mmol, 10.0 equiv) via syringe pump over 20 h at 23 °C. Ethyl acetate (10 mL) was added and the mixture was filtered. The filtrate was washed with a saturated aqueous sodium thiosulfate solution (10 mL) and a saturated aqueous sodium chloride solution (10 mL). The washed solution was dried over sodium sulfate and filtered. The dried filtrate was concentrated under reduced pressure and the residue was dissolved in a mixture of chloroform (2 mL) and ethyl acetate (4 mL). To that solution were added 2,6-di-*tert*-butylpyridine (0.426 mL, 1.97 mmol, 2.50 equiv) and copper(II) bromide (387 mg, 1.73 mmol, 2.20 equiv) and the resulting suspension was stirred and heated to 100 °C for 4 h. The suspension was allowed to cool down to 23 °C and then filtered through a short pad of celite which was then washed with ethyl acetate (2 × 20 mL). The resulting solution was concentrated under reduced pressure and the residue was dissolved in chloroform (5 mL). To the solution was added tetra-*n*-butylammonium tribromide (380 mg, 0.788 mmol, 1.00 equiv) and the resulting mixture was stirred and heated to 60 °C for 8 h. The mixture was allowed to cool to 23 °C and ethyl acetate (20 mL) was added. The mixture was washed with a saturated aqueous sodium thiosulfate solution (20 mL) and a saturated aqueous sodium chloride solution (20 mL). The washed solution was dried over sodium sulfate and filtered. The dried filtrate was concentrated under reduced pressure and the residue was purified by flash chromatography on silica gel (10% diethyl ether in pentane grading to 15% diethyl ether in pentane) which afforded the vinylogous lactone **28** as a colorless oil (109 mg, 0.207 mmol, 27% over 3 steps).

Synthesis of ester 14

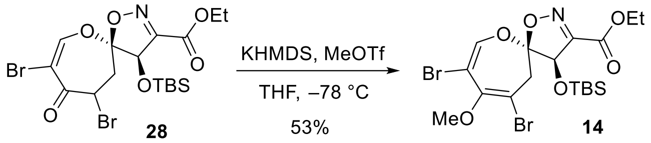

To a vigorously stirred colorless solution of vinylogous lactone **28** (109 mg, 207 μmol, 1 equiv) in tetrahydrofuran (1 mL) cooled to –78 °C was added dropwise a solution of potassium bis(trimethylsilyl)amide (1.00 M in tetrahydrofuran, 230 μL, 230 μmol, 1.11 equiv) immediately followed by addition of methyl trifluoromethanesulfonate (50.0 μL, 445 μmol, 2.15 equiv). The resulting dark red solution was allowed to warm up to 0 °C during which the color dissipated to faintly yellow. After stirring for 15 min at 0 °C, a saturated aqueous sodium bicarbonate solution (10 mL) was added. The mixture was extracted with ethyl acetate (2 × 10 mL). The organic solution was washed with a saturated aqueous sodium chloride solution (20 mL). The washed solution was dried over sodium sulfate and filtered. The dried filtrate was concentrated under reduced pressure and the residue was purified by flash chromatography on silica gel (0% diethyl ether in pentane grading to 5% diethyl ether in pentane) which afforded the ester **14** as a pale-yellow oil (59.1 mg, 109 μmol, 53%).

TLC (20% ethyl acetate in cyclohexane): R_f = 0.84 (UV, $KMnO_4$).

^{1}H NMR (400 MHz, $CDCl_3$, 25 °C): δ 6.98 (s, 1H), 4.96 (s, 1H), 4.45 – 4.28 (m, 2H), 3.69 (s, 3H), 3.55 (d, J = 16.1 Hz, 1H), 2.80 (d, J = 16.1 Hz, 1H), 1.38 (t, J = 7.1 Hz, 3H), 0.91 (s, 9H), 0.20 – 0.16 (m, 6H) ppm.

^{13}C NMR (101 MHz, $CDCl_3$, 25 °C): δ 159.6, 155.1, 148.5, 144.0, 115.6, 102.6, 101.2, 80.8, 62.6, 59.2, 38.2, 25.8, 18.3, 14.2, –4.6, –4.7 ppm.

IR (ATR, neat): $\tilde{\nu}$ = 2956 (*w*), 2933 (*w*), 2859 (*w*), 1727 (*m*), 1626 (*w*), 1594 (*w*), 1471 (w), 1380 (*w*), 1328 (*w*), 1264 (*m*), 1217 (*w*), 1192 (*w*), 1142 (*s*), 1122 (*s*), 1068 (*w*), 1031 (*w*), 992 (*w*), 918 (*m*), 840 (*w*), 814 (*w*), 783 (*m*) cm^{-1}.

HRMS (ESI): calcd. for $C_{18}H_{27}Br_2NNaO_6Si$ [M+Na]$^+$: 561.9867; found: 561.9855.

Synthesis of alcohol S17

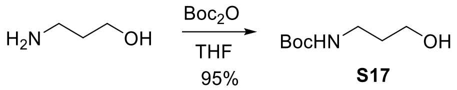

To a vigorously stirred solution of 3-aminopropanol (2.07 g, 27.6 mmol, 1 equiv) in tetrahydrofuran (24 mL) was added di-*tert*-butyl dicarbonate (7.35 g, 33.7 mmol, 1.22 equiv) at 23 °C and the resulting solution was stirred for 20 h. The solution was concentrated under reduced pressure and the residue was purified by flash chromatography on silica gel (50% diethyl ether in pentane) which afforded the alcohol **S17** as a colorless viscous oil (4.60 g, 26.3 mmol, 95%).

^{1}H NMR (400 MHz, $CDCl_3$, 25 °C): δ 4.74 (br s, 1H), 3.76 – 3.57 (m, 2H), 3.37 – 3.18 (m, 2H), 2.90 (br s, 1H), 1.72 – 1.59 (m, 2H), 1.45 (s, 9H) ppm. The analytical data matched those published before.[4]

Synthesis of bromide S18

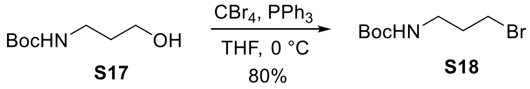

To a stirred solution of carbon tetrabromide (2.84 g, 8.56 mmol, 1.50 equiv) in tetrahydrofuran (10 mL) was added triphenylphosphine (2.25 g, 8.56 mmol, 1.50 equiv) in one portion at 0 °C and the resulting suspension was stirred at that temperature for 15 min. Solution of alcohol **S17** (1.00 g, 5.71 mmol, 1 equiv) in tetrahydrofuran (5 mL) was added dropwise over 1 min at 0 °C and the resulting suspension was stirred at that temperature for 2 h. The mixture was concentrated under reduced pressure and the residue was purified by flash chromatography on silica gel (20% pentane in dichloromethane grading to 100% dichloromethane) which afforded the bromide **S18** as a colorless oil (1.09 g, 4.56 mmol, 80%).

^{1}H NMR (400 MHz, $CDCl_3$, 25 °C): δ 4.65 (br s, 1H), 3.44 (t, J = 6.6 Hz, 2H), 3.33 – 3.21 (m, 2H), 2.12 – 1.97 (m, 2H), 1.44 (s, 9H) ppm. The analytical data matched those published before.[5]

Synthesis of phthalimide S19

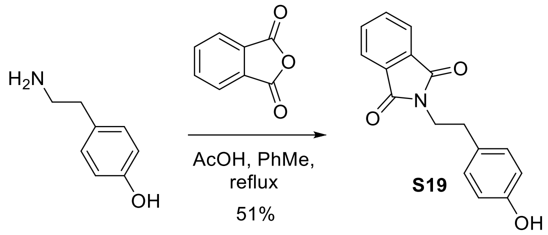

A stirred mixture of tyramine (2.00 g, 14.6 mmol, 1 equiv), phthalic anhydride (2.16 g, 14.6 mmol, 1.00 equiv), toluene (30 mL) and acetic acid (15 mL) was heated to reflux in a Dean–Stark apparatus for 10 h. The mixture was allowed to cool to 23 °C and was filtered. The solid was washed with ice-cold isopropanol (50 mL) and ice-cold cyclohexane (50 mL). The washed solid was dried under reduced pressure which afforded the phthalimide **S19** as pale brown powder (1.98 g, 7.42 mmol, 51%) which did not require further purification.

^{1}H NMR (400 MHz, DMSO-*d*$_6$, 25 °C): δ 9.18 (s, 1H), 7.87 – 7.79 (m, 4H), 6.99 – 6.93 (m, 2H), 6.65 – 6.60 (m, 2H), 3.74 (t, *J* = 7.5 Hz, 2H), 2.79 (t, *J* = 7.3 Hz, 2H) ppm. The analytical data matched those published before.[6]

Synthesis of phthalimide S20

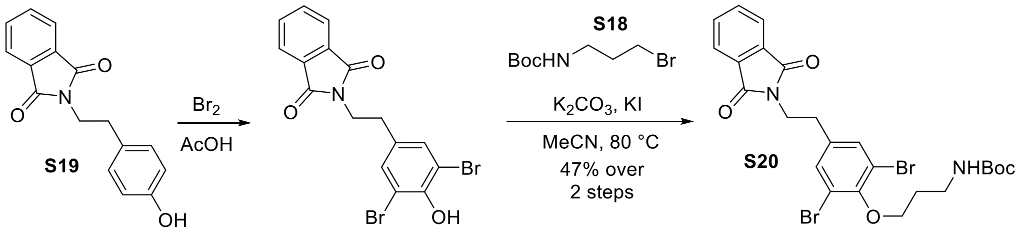

To a stirred suspension of phthalimide **S19** (1.98 g, 7.42 mmol, 1 equiv) in acetic acid (10 mL) was added dropwise bromine (0.841 mL, 16.3 mmol, 2.20) at 23 °C and the resulting mixture was stirred at that temperature for 20 h. The mixture was filtered and the solid was washed with diethyl ether (20 mL) and cyclohexane (20 mL). The washed solid was dried under reduced pressure which afforded a pale brown powder (2.53 g). To a suspension of the brown powder (1.30 g) in acetonitrile (5 mL) were added potassium carbonate (634 mg, 4.59 mmol, 1.20 equiv), potassium iodide (76.2 mg, 0.459 mmol, 0.120 equiv) and bromide **S18** (1.09 g, 4.59 mmol, 1.20 equiv) and the resulting mixture was vigorously stirred and heated to 80 °C for 4 h. The mixture was allowed to cool to

23 °C and acetonitrile was removed under reduced pressure. To the residue was added water (10 mL) and the mixture was extracted with ethyl acetate (20 mL). The organic solution was washed with a saturated aqueous sodium chloride solution (10 mL). The washed solution was dried over sodium sulfate and filtered. The dried filtrate was concentrated under reduced pressure and flash chromatography on silica gel (20% ethyl acetate in pentane) afforded the phthalimide **S20** as a white solid (1.04 g, 1.79 mmol, 47% over 2 steps).

Note: The equivalents in the second step and the overall yield were calculated with respect to the fact that only 1.30 g out of the total 2.53 g of the product of the first step were used.

TLC (20% ethyl acetate in cyclohexane): R_f = 0.33 (UV, $KMnO_4$).

Melting point: 129 – 130 °C.

^{1}H NMR (400 MHz, $CDCl_3$, 25 °C): δ 7.88 – 7.82 (m, 2H), 7.76 – 7.69 (m, 2H), 7.41 (s, 2H), 4.94 (br s, 1H), 4.03 (t, J = 5.8 Hz, 2H), 3.90 – 3.84 (m, 2H), 3.50 – 3.35 (m, 2H), 2.94 – 2.86 (m, 2H), 2.07 – 1.97 (m, 2H), 1.45 (s, 9H) ppm.

^{13}C NMR (101 MHz, $CDCl_3$, 25 °C): δ 168.2, 156.2, 151.9, 136.9, 134.2, 133.2, 132.1, 123.5, 118.4, 79.3, 71.4, 38.8, 38.2, 33.5, 30.2, 28.6 ppm.

IR (ATR, neat): $\tilde{\nu}$ = 3360 (*w*), 1702 (*s*), 1683 (*s*), 1522 (*m*), 1451 (*m*), 1400 (*m*), 1362 (*m*), 1325 (*m*), 1271 (*m*), 1241 (*m*), 1171 (*m*), 1104 (*m*), 1053 (*m*), 997 (*m*), 931 (*w*), 907 (*w*), 871 (*m*), 737 (*m*), 721 (*m*), 712 (*m*) cm^{-1}.

HRMS (ESI): calcd. for $C_{24}H_{26}Br_2N_2NaO_5$ [M+Na]$^+$: 603.0101; found: 603.0087.

Synthesis of ammonium salt 29

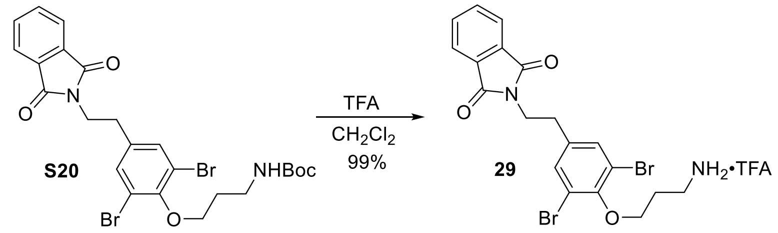

To a solution of phthalimide **S20** (817 mg, 1.40 mmol, 1 equiv) in dichloromethane (5 mL) was added trifluoroacetic acid (0.540 mL, 7.02 mmol, 5.01 equiv) and the resulting solution was stirred at 23 °C for 20 h. The solution was concentrated under reduced

pressure and the residue was purified by flash chromatography on silica gel (10% methanol in dichloromethane) which afforded the ammonium salt **29** as a white solid (830 mg, 1.39 mmol, 99%).

TLC (10% methanol in dichloromethane): R_f = 0.23 (UV, $KMnO_4$).

Melting point: Decomposition at 170 °C.

^{1}H NMR (400 MHz, CD_3OD, 25 °C): δ 7.86 – 7.77 (m, 4H), 7.46 (s, 2H), 4.09 (t, J = 5.7 Hz, 2H), 3.89 (t, J = 7.3 Hz, 2H), 3.30 – 3.25 (m, 2H), 2.94 (t, J = 7.1 Hz, 2H), 2.23 – 2.14 (m, 2H) ppm.

^{13}C NMR (101 MHz, CD_3OD, 25 °C): δ 169.5, 152.5, 139.5, 135.5, 134.5, 133.2, 124.2, 118.8, 71.6, 39.8, 38.9, 34.0, 29.0 ppm.

^{19}F NMR (376 MHz, CD_3OD, 25 °C): δ –77.0 ppm.

IR (ATR, neat): $\tilde{\nu}$ = 3453 (*w*, *br*), 1770 (*w*), 1706 (*s*), 1664 (*s*), 1542 (*w*), 1459 (*m*), 1431 (*m*), 1389 (*m*), 1362 (*m*), 1255 (*w*), 1182 (*s*), 1134 (*s*), 1096 (*m*), 1067 (*m*), 999 (*m*), 881 (*w*), 848 (*m*), 799 (*m*), 716 (*s*) cm^{-1}.

HRMS (ESI): calcd. for $C_{19}H_{19}Br_2N_2O_3$ [M–CF_3CO_2H+H]$^+$: 480.9757; found: 480.9750.

Synthesis of amide 30

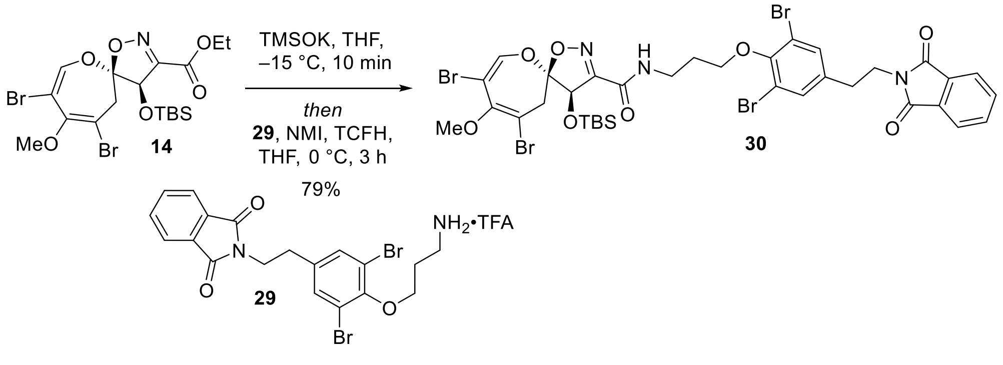

To a vigorously stirred suspension of potassium trimethylsilanolate (31.0 mg, 242 μmol, 3.04 equiv) in tetrahydrofuran (0.3 mL) cooled to –15 °C was added a solution of ester **14** (43.0 mg, 79.4 μmol, 1 equiv) in tetrahydrofuran (0.5 mL). After stirring at –15 °C for 10 min, ammonium salt **29** (102 mg, 171 μmol, 2.15 equiv), *N*-methylimidazole (50.0 μL, 630 μmol, 7.93 equiv) and chloro-*N*,*N*,*N´*,*N´*-tetramethylformamidinium hexafluorophosphate (110 mg, 397 μmol, 5.00 equiv) were added at –15 °C. The resulting

mixture was allowed to warm up to 0 °C and was stirred at that temperature for 3 h. A saturated aqueous sodium bicarbonate solution (10 mL) was added and the mixture was allowed to warm up to 23 °C. The mixture was extracted with ethyl acetate (2 × 10 mL). The organic solution was washed with a saturated aqueous sodium chloride solution (10 mL). The washed solution was dried over sodium sulfate and filtered. The dried filtrate was concentrated under reduced pressure and the residue was purified by flash chromatography on silica gel (20% diethyl ether in pentane grading to 40% diethyl ether in pentane) which afforded the amide **30** as a colorless oil (61.0 mg, 62.4 μmol, 79%).

TLC (20% ethyl acetate in cyclohexane): R_f = 0.47 (UV, $KMnO_4$).

^{1}H NMR (400 MHz, $CDCl_3$, 25 °C): δ 7.88 – 7.82 (m, 2H), 7.76 – 7.69 (m, 2H), 7.41 (s, 2H), 7.06 (t, J = 6.1 Hz, 1H), 6.98 (s, 1H), 5.02 (s, 1H), 4.05 (t, J = 5.6 Hz, 2H), 3.90 – 3.83 (m, 2H), 3.76 – 3.63 (m, 5H), 3.57 (d, J = 16.0 Hz, 1H), 2.94 – 2.86 (m, 2H), 2.82 (d, J = 16.1 Hz, 1H), 2.14 – 2.03 (m, 2H), 0.91 (s, 9H), 0.22 (s, 3H), 0.19 (s, 3H) ppm.

^{13}C NMR (101 MHz, $CDCl_3$, 25 °C): δ 168.2, 158.5, 157.1, 151.7, 148.5, 144.3, 137.1, 134.3, 133.2, 132.1, 123.5, 118.3, 115.8, 102.5, 100.9, 80.7, 70.9, 59.3, 38.8, 38.1, 37.2, 33.5, 29.5, 25.9, 18.4, –4.6, –4.9 ppm.

IR (ATR, neat): $\tilde{\nu}$ = 2932 (*w*), 2857 (*w*), 1773 (*w*), 1713 (*s*), 1679 (*m*), 1626 (*w*), 1596 (*w*), 1539 (*w*), 1457 (*w*), 1395 (*m*), 1362 (*w*), 1257 (*m*), 1191 (*w*), 1141 (*m*), 1068 (*w*), 995 (*w*), 956 (*w*), 908 (*w*), 840 (*m*), 784 (*w*), 736 (*m*), 719 (*m*) cm^{-1}.

HRMS (ESI): calcd. for $C_{35}H_{39}Br_4N_3NaO_8Si$ [M+Na]$^+$: 995.9132; found: 995.9105.

Synthesis of psammaplysin A (1)

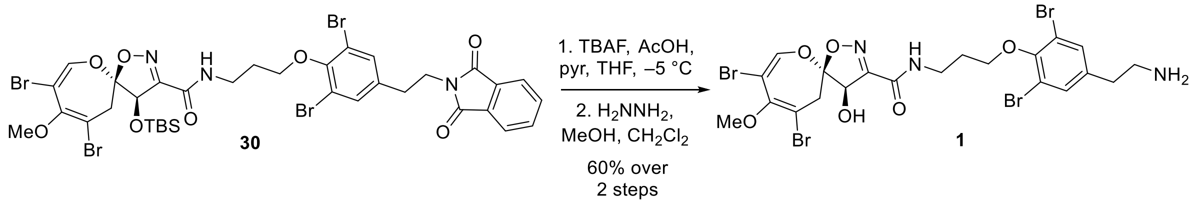

To a solution of amide **30** (60.8 mg, 62.2 μmol) in tetrahydrofuran (0.6 mL) were added pyridine (20.1 μl, 249 μmol, 4.00 equiv) and acetic acid (10.7 μl, 187 μmol, 3.01 equiv) at –5 °C followed by dropwise addition of a solution of tetra-*n*-butylammonium fluoride (1.00 M in tetrahydrofuran, 93.3 μl, 93.3 μmol, 1.50 equiv). The resulting solution was stirred at –5 °C for 30 min before it was allowed to warm up to 23 °C. After 10 min, a

saturated aqueous sodium bicarbonate solution (10 mL) was added. The mixture was extracted with ethyl acetate (2 × 10 mL). The organic solution was washed with a saturated aqueous sodium chloride solution (10 mL). The washed solution was dried over sodium sulfate and filtered. The dried filtrate was concentrated under reduced pressure which afforded a beige solid (45.3 mg). The solid was suspended in a mixture of methanol (0.5 mL) and dichloromethane (0.5 mL) and to the stirred suspension was added hydrazine hydrate (25.7 μl, 529 μmol, 8.50 equiv). After stirring at 23 °C for 10 h, the mixture was concentrated under reduced pressure and the residue was purified by flash chromatography on silica gel (5% methanol in dichloromethane grading to 10% methanol in dichloromethane) which afforded psammaplysin A (**1**) as a colorless foam (27.5 mg, 37.5 μmol, 60% over 2 steps).

TLC (10% methanol in dichloromethane): R_f = 0.36 (UV, $KMnO_4$).

^{1}H NMR (400 MHz, CD_2Cl_2, 25 °C): δ 7.38 (s, 2H), 7.11 (t, J = 5.9 Hz, 1H), 7.04 (s, 1H), 5.08 (s, 1H), 4.10 (t, J = 5.8 Hz, 2H), 3.72 – 3.60 (m, 5H), 3.37 (d, J = 16.0 Hz, 1H), 3.09 (d, J = 16.0 Hz, 1H), 2.90 (t, J = 6.8 Hz, 2H), 2.65 (t, J = 6.7 Hz, 2H), 2.14 – 2.05 (m, 2H) ppm.

^{1}H NMR (400 MHz, CD_3OD, 25 °C): δ 7.47 (s, 2H), 7.14 (s, 1H), 4.98 (s, 1H), 4.07 (t, J = 6.0 Hz, 2H), 3.67 – 3.59 (m, 5H), 3.39 (d, J = 16.1 Hz, 1H), 3.07 (d, J = 16.1 Hz, 1H), 2.91 (t, J = 7.2 Hz, 2H), 2.73 (t, J = 7.2 Hz, 2H), 2.18 – 2.08 (m, 2H) ppm.

^{13}C NMR (101 MHz, CD_3OD, 25 °C): δ 160.6, 158.8, 152.7, 149.8, 146.8, 140.3, 134.2, 120.8, 119.1, 104.5, 104.3, 80.4, 72.1, 59.3, 43.7, 38.4, 38.2, 38.0, 30.6 ppm.

IR (ATR, neat): $\tilde{\nu}$ = 3303 (*w*, *br*), 2935 (*w*), 2878 (*w*), 1666 (*s*), 1625 (*m*), 1594 (*m*), 1541 (*m*), 1457 (*m*), 1383 (*w*), 1329 (*w*), 1257 (*m*), 1196 (*w*), 1147 (*m*), 1118 (*m*), 1044 (*w*), 991 (*w*), 955 (*w*), 898 (*w*), 774 (*w*), 738 (*w*) cm^{-1}.

HRMS (ESI): calcd. for $C_{21}H_{24}Br_4N_3O_6$ $[M+H]^+$: 729.8393; found: 729.8374.

Synthesis of acetamide S21

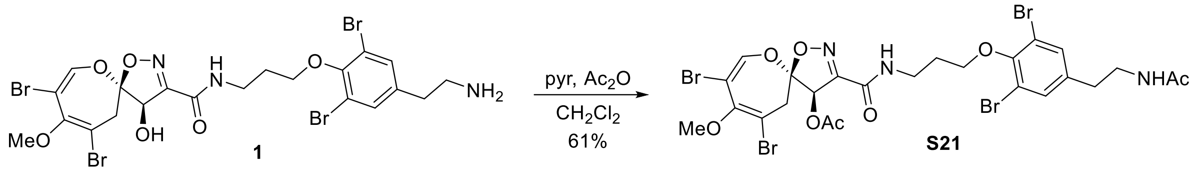

To a solution of psammaplysin A (**1**) (11.8 mg, 16.1 μmol, 1 equiv) in dichloromethane (0.5 mL) were added pyridine (130 μl, 1.61 mmol, 100 equiv) and acetic anhydride (76.0 μl, 0.805 mmol, 50.0 equiv) at 23 °C and the resulting solution was stirred for 5 h. The solution was poured into a saturated aqueous sodium bicarbonate solution (10 mL) and the mixture was extracted with ethyl acetate (2 × 10 mL). The combined organic solutions were washed with a saturated aqueous sodium chloride solution (10 mL). The washed solution was dried over sodium sulfate and filtered. The dried filtrate was concentrated under reduced pressure and the residue was purified by flash chromatography on silica gel (50% ethyl acetate in dichloromethane) which afforded the acetamide **S21** as a colorless oil (8.0 mg, 9.8 μmol, 61%).

TLC (50% ethyl acetate in dichloromethane): R_f = 0.30 (UV, $KMnO_4$).

^{1}H NMR (400 MHz, CD_2Cl_2, 25 °C): δ 7.38 (s, 2H), 7.11 (br t, J = 5.5 Hz, 1H), 7.07 (s, 1H), 6.35 (s, 1H), 5.54 (br t, J = 4.9 Hz, 1H), 4.09 (t, J = 5.4 Hz, 2H), 3.70 – 3.63 (m, 5H), 3.45 – 3.38 (m, 2H), 3.21 (d, J = 16.1 Hz, 1H), 3.05 (d, J = 16.1 Hz, 1H), 2.73 (t, J = 7.0 Hz, 2H), 2.19 (s, 3H), 2.14 – 2.06 (m, 2H), 1.90 (s, 3H) ppm.

^{13}C NMR (101 MHz, CD_2Cl_2, 25 °C): δ 170.2, 168.5, 157.6, 154.5, 151.7, 149.6, 146.0, 138.8, 133.4, 122.0, 118.4, 106.0, 102.7, 77.4, 71.8, 59.5, 40.7, 38.0, 37.7, 34.9, 29.7, 23.4, 21.2 ppm.

IR (ATR, neat): $\tilde{\nu}$ = 3296 (*w*, *br*), 2935 (*w*), 1763 (*m*), 1657 (*s*), 1597 (*m*), 1541 (*s*), 1457 (*m*), 1372 (*m*), 1257 (*m*), 1205 (*s*), 1148 (*m*), 1119 (*m*), 1040 (*m*), 992 (*w*), 956 (*w*), 922 (*m*), 738 (*m*), 710 (*w*) cm^{-1}.

HRMS (ESI): calcd. for $C_{25}H_{28}Br_4N_3O_8$ $[M+H]^+$: 813.8604; found: 813.8599.

Comparison of Natural and Synthetic Psammaplysin A

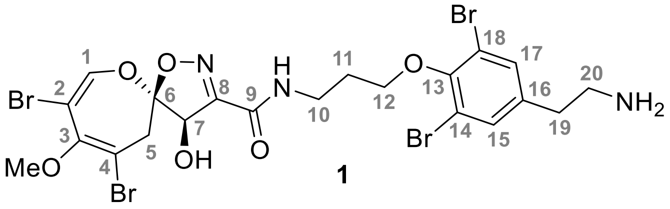

1

^{1}H NMR (CD_2Cl_2):

Position	Natural **1**[7]	Synthetic **1**	Δ*δ* (ppm)
1	7.04 (s, 1H)	7.04 (s, 1H)	0.00
5	3.37, 3.08 (AB q, *J* = 16 Hz, 2H)	3.37 (d, *J* = 16.0 Hz, 1H), 3.09 (d, *J* = 16.0 Hz, 1H)	0.00, 0.01
7	5.05 (s, 1H)	5.08 (s, 1H)	0.03
10	3.68 (t, *J* = 6 Hz, 2H)	3.67 (m, 2H)	0.01
11	2.1 (2H, obscured by H_2O)	2.09 (m, 2H)	0.01
12	4.08 (t, *J* = 6 Hz, 2H)	4.10 (t, *J* = 5.8 Hz, 2H)	0.02
15, 17	7.38 (s, 2H)	7.38 (s, 2H)	0.00
19	2.66 (t, *J* = 7 Hz, 2H)	2.65 (t, *J* = 6.7 Hz, 2H)	0.01
20	2.90 (t, *J* = 7 Hz, 2H)	2.90 (t, *J* = 6.8 Hz, 2H)	0.00
-OMe	3.66 (s, 3H)	3.66 (s, 3H)	0.00
-CONH-	7.15 (br t, *J* = 6 Hz, 1H)	7.11 (br t, *J* = 5.9 Hz, 1H)	0.04
-NH_2	5.4 (br s, 1H)	Not observed	-

^{1}H NMR (CD_3OD):

Position	Natural **1**[7]	Synthetic **1**	$\Delta\delta$ (ppm)
1	7.13 (s, 1H)	7.14 (s, 1H)	0.01
5	3.38, 3.05<br>(AB q, *J* = 16.1 Hz, 2H)	3.39 (d, *J* = 16.1 Hz, 1H), 3.07<br>(d, *J* = 16.1 Hz, 1H)	0.01,<br>0.02
7	4.97 (s, 1H)	4.98 (s, 1H)	0.01
10	3.61 (t, *J* = 7.0 Hz, 2H)	3.62 (m, 2H)	0.01
11	2.13 (dt, *J* = 7.0, 6.0, 2H)	2.13 (m, 2H)	0.00
12	4.06 (t, *J* = 6.0 Hz, 2H)	4.07 (t, *J* = 6.0 Hz, 2H)	0.01
15, 17	7.48 (s, 2H)	7.47 (s, 2H)	0.01
19	2.76 (dd, *J* = 7.5, 7.0 Hz, 2H)	2.73 (t, *J* = 7.2 Hz, 2H)	0.03
20	2.96 (dd, *J* = 7.5, 7.0 Hz, 2H)	2.91 (t, *J* = 7.2 Hz, 2H)	0.05
-OMe	3.64 (s, 3H)	3.65 (s, 3H)	0.01
$-NH_2$	5.48 (s, 1H)	Not observed	-

^{13}C NMR (CD_3OD):

Position	Natural **1**[7]	Synthetic **1**	$\Delta\delta$ (ppm)
1	146.3	146.8	0.5
2	104.3	104.5	0.2
3	149.4	149.9	0.5
4	103.9	104.3	0.4
5	38.2	38.5	0.3
6	120.4	120.8	0.4
7	80.2	80.4	0.2
8	158.2	158.8	0.6
9	160.2	160.6	0.4
10	37.9	38.1	0.2
11	29.9	30.6	0.7
12	71.9	72.1	0.2
13	153.0	152.8	0.2
14, 18	119.1	119.1	0.0
15, 17	134.1	134.2	0.1
16	136.9	140.1	3.2
19	32.9	38.0	5.1
20	41.5	43.6	2.1
-OMe	59.3	59.3	0.0

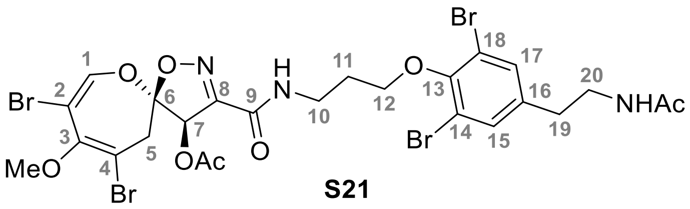

S21

^{1}H NMR (CD_2Cl_2):

Position	**S21** from natural **1**[7]	**S21** from synthetic **1**	Δ*δ* (ppm)
1	7.07 (s, 1H)	7.07 (s, 1H)	0.00
5	3.20, 3.04 (AB q, *J* = 16.0 Hz, 2H)	3.21 (d, *J* = 16.1 Hz, 1H), 3.05 (d, *J* = 16.1 Hz, 1H)	0.01, 0.01
7	6.34 (s, 1H)	6.35 (s, 1H)	0.01
10	3.66 (t, *J* = 6.3 Hz, 2H)	3.67 (m, 2H)	0.01
11	2.10 (dt, *J* = 6.3, 5.7 Hz, 2H)	2.10 (m, 2H)	0.00
12	4.08 (t, *J* = 5.7 Hz, 2H)	4.09 (t, *J* = 5.4 Hz, 2H)	0.01
15, 17	7.38 (s, 2H)	7.38 (s, 2H)	0.00
19	2.72 (t, *J* = 7.0 Hz, 2H)	2.73 (t, *J* = 7.0 Hz, 2H)	0.01
20	3.41 (m, 2H)	3.41 (m, 2H)	0.00
-OMe	3.65 (s, 3H)	3.65 (s, 3H)	0.00
-CON*H*-	7.10 (br t, *J* = 6 Hz, 1H)	7.10 (br t, *J* = 5.5 Hz, 1H)	0.00
-N*H*COCH$_3$	5.53 (br t, *J* = 7 Hz, 1H)	5.54 (br t, *J* = 4.9 Hz, 1H)	0.01
-NHCO*CH$_3$*	1.90 (s, 3H)[a]	1.90 (s, 3H)	0.00
-OCO*CH$_3$*	2.18 (s, 3H)[a]	2.19 (s, 3H)	0.01

[a] Our 2D NMR data suggests that these signals were previously incorrectly assigned[7]

^{13}C NMR (CD_2Cl_2):

Position	**S21** from natural **1**[7]	**S21** from synthetic **1**	$\Delta\delta$ (ppm)
1	145.9	146.0	0.1
2	105.9	106.0	0.1
3	149.4	149.5	0.1
4	102.6	102.7	0.1
5	37.9	38.0	0.1
6	121.8	122.0	0.2
7	77.3	77.4	0.1
8	154.4	154.5	0.1
9	157.5	157.6	0.1
10	37.6	37.7	0.1
11	29.6	29.7	0.1
12	71.1	71.8	0.7
13	151.5	151.7	0.2
14, 18	118.3	118.4	0.1
15, 17	133.3	133.4	0.1
16	138.7	138.8	0.1
19	34.8	34.9	0.1
20	40.7	40.7	0.0
-OMe	59.4	59.5	0.1
-NH*CO*CH_3	170.3	170.2	0.1
-NHCO$\mathit{CH_3}$	23.3	23.4	0.1
-O*CO*CH_3	168.4	168.5	0.1
-OCO$\mathit{CH_3}$	21.2	21.2	0.0

NMR Spectra

^{1}H NMR (400 MHz, $CDCl_3$, 25 °C) of ester **S1a**:

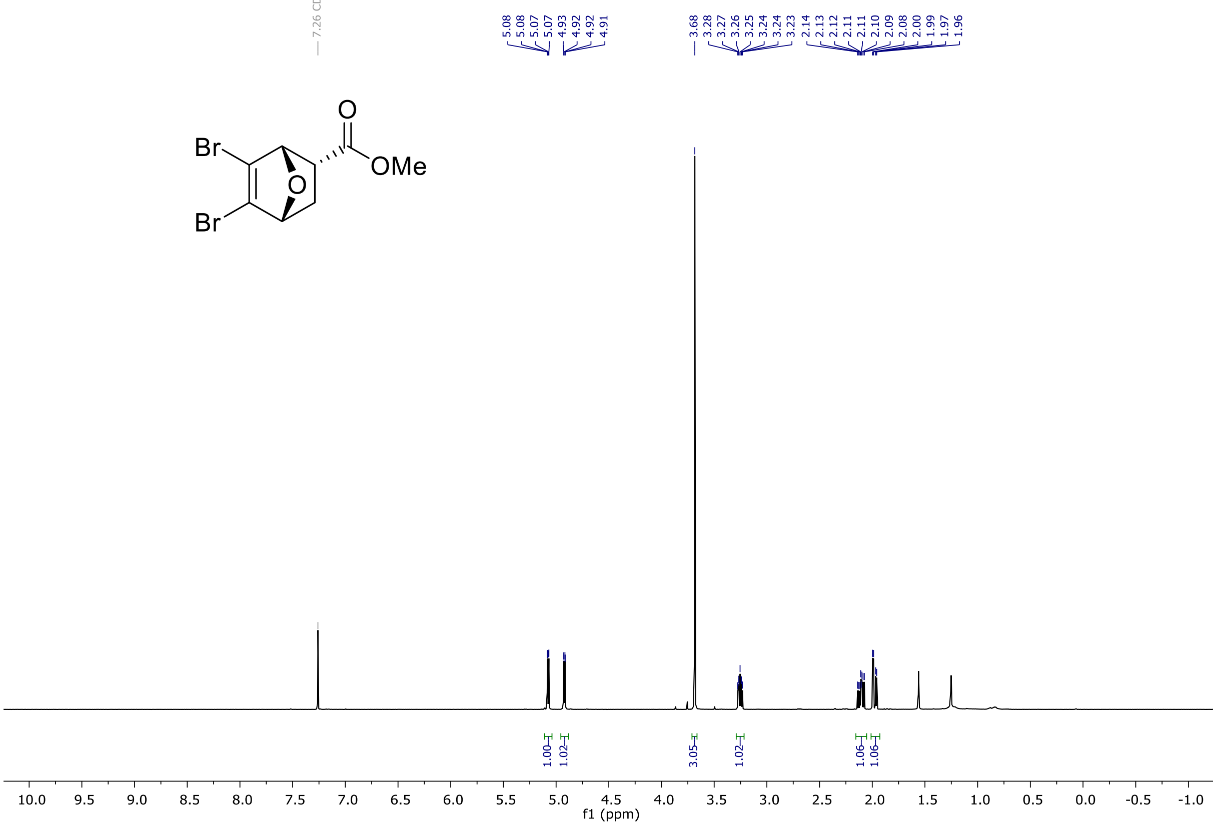

^{13}C NMR (101 MHz, $CDCl_3$, 25 °C) of ester **S1a**:

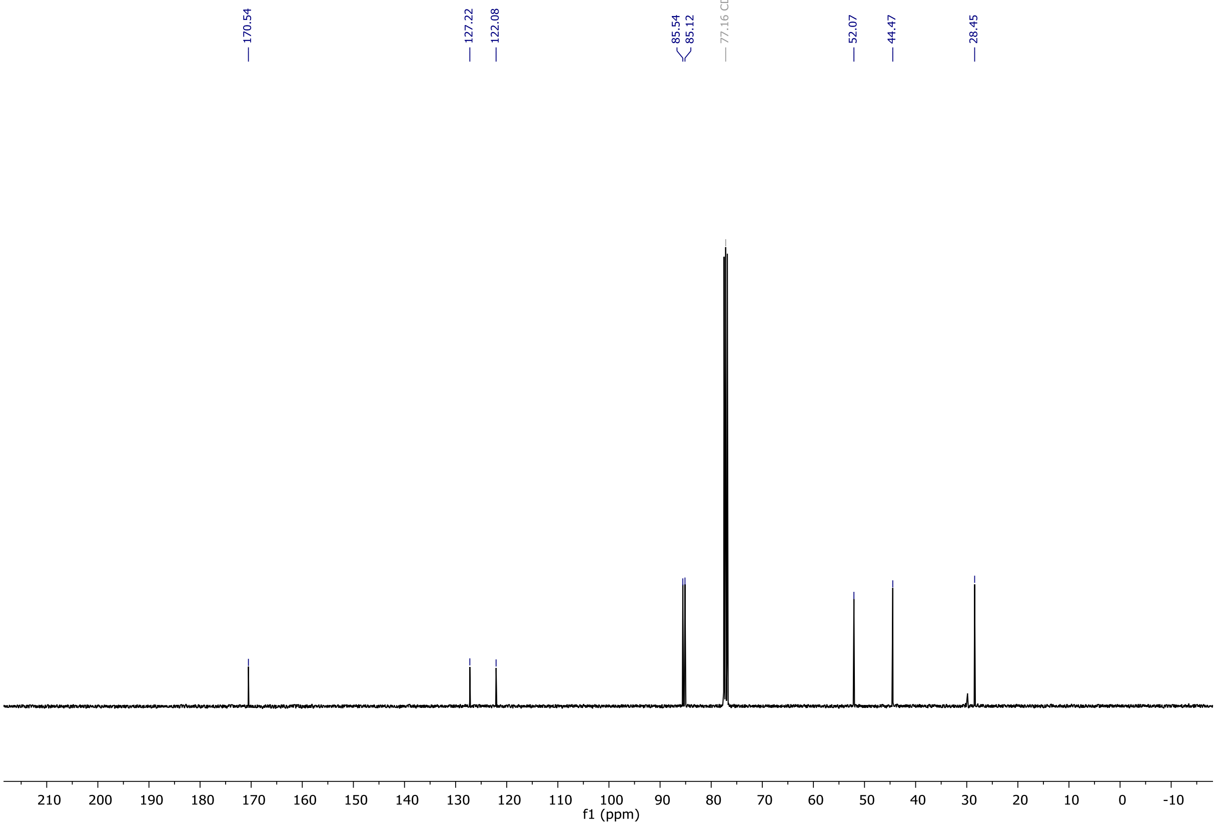

^{1}H NMR (400 MHz, $CDCl_3$, 25 °C) of ester **S1b**:

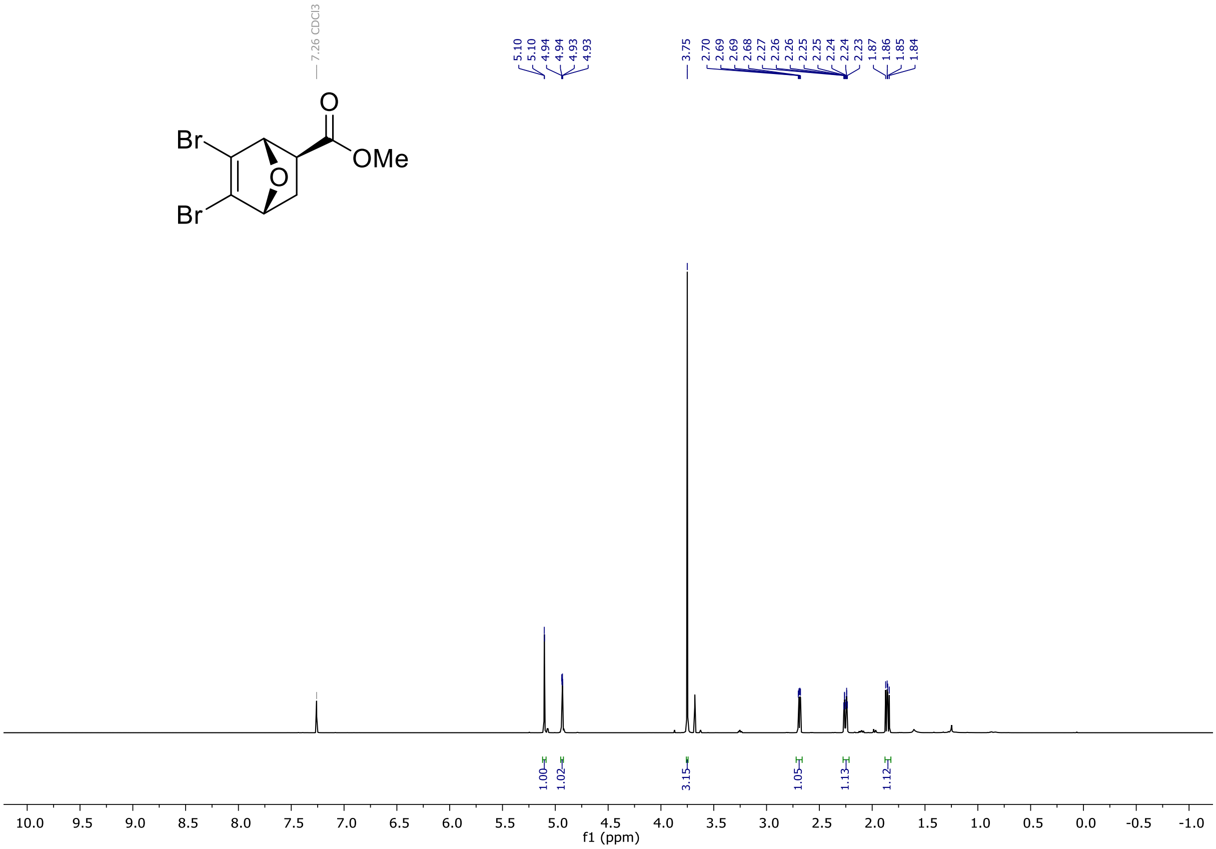

^{13}C NMR (101 MHz, $CDCl_3$, 25 °C) of ester **S1b**:

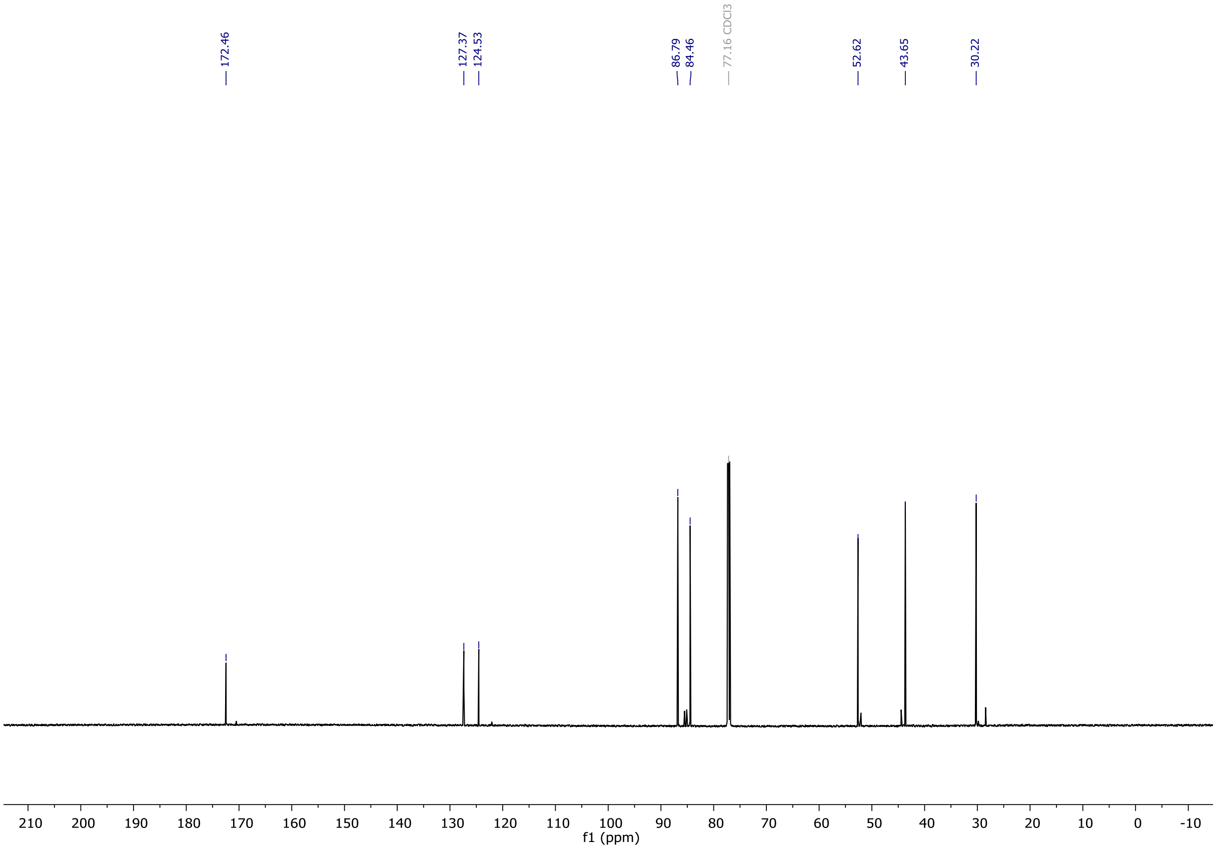

^{1}H NMR (400 MHz, $CDCl_3$, 25 °C) of ester **S2**:

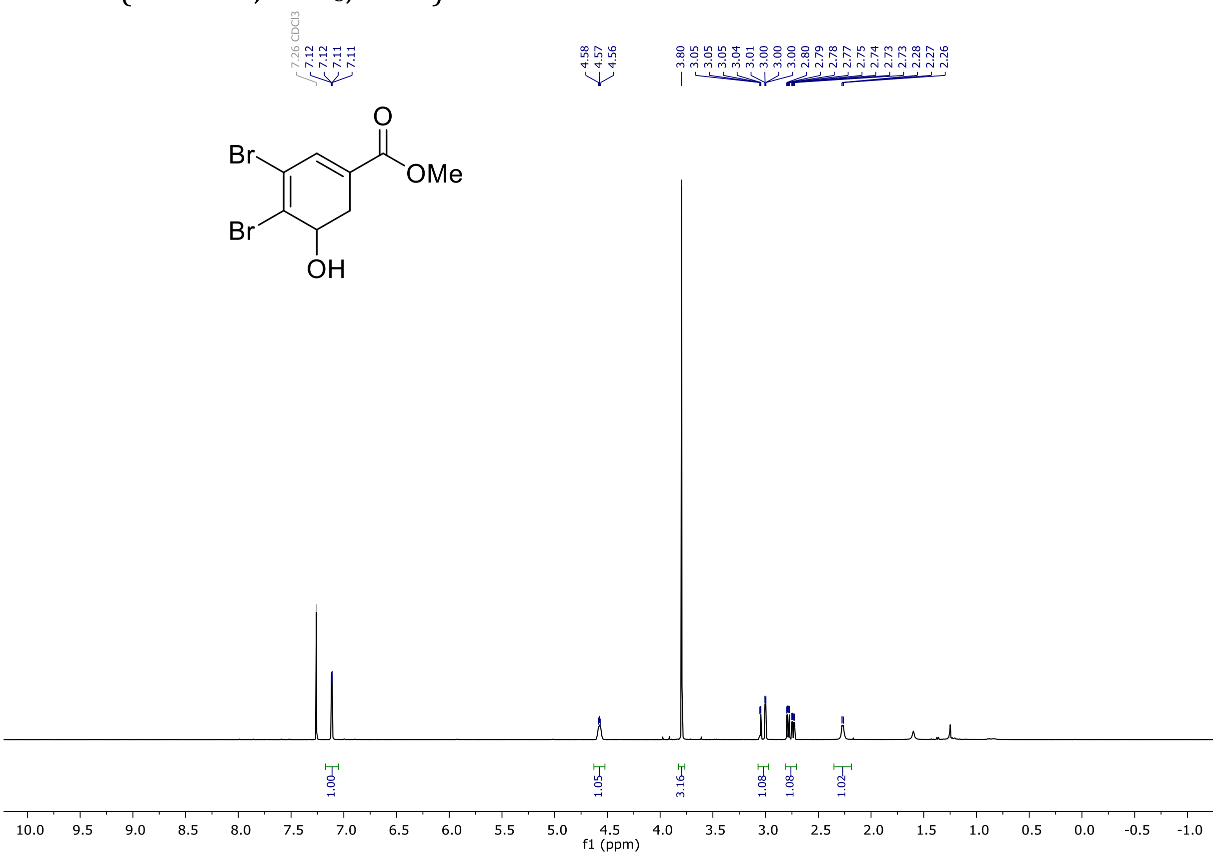

^{13}C NMR (101 MHz, $CDCl_3$, 25 °C) of ester **S2**:

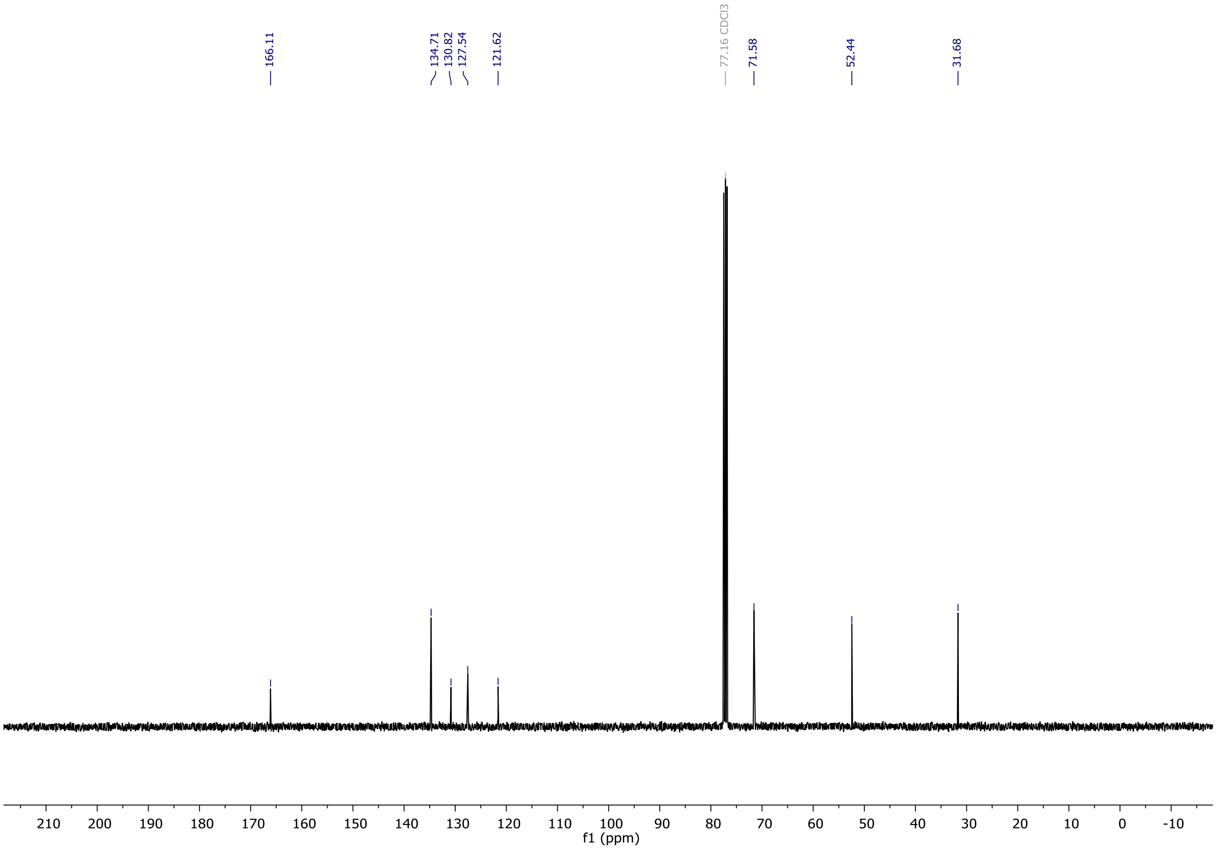

^{1}H NMR (400 MHz, $CDCl_3$, 25 °C) of ester **S3**:

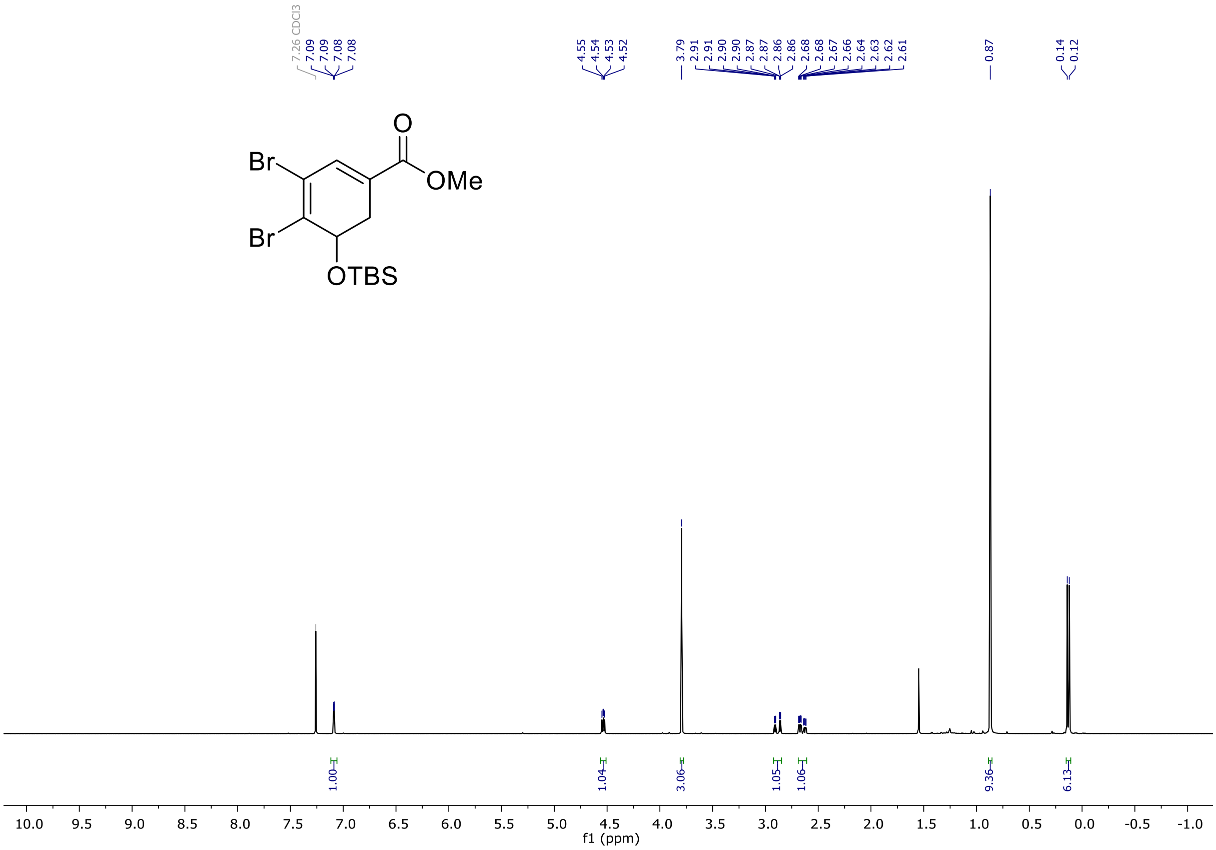

^{13}C NMR (151 MHz, $CDCl_3$, 30 °C) of ester **S3**:

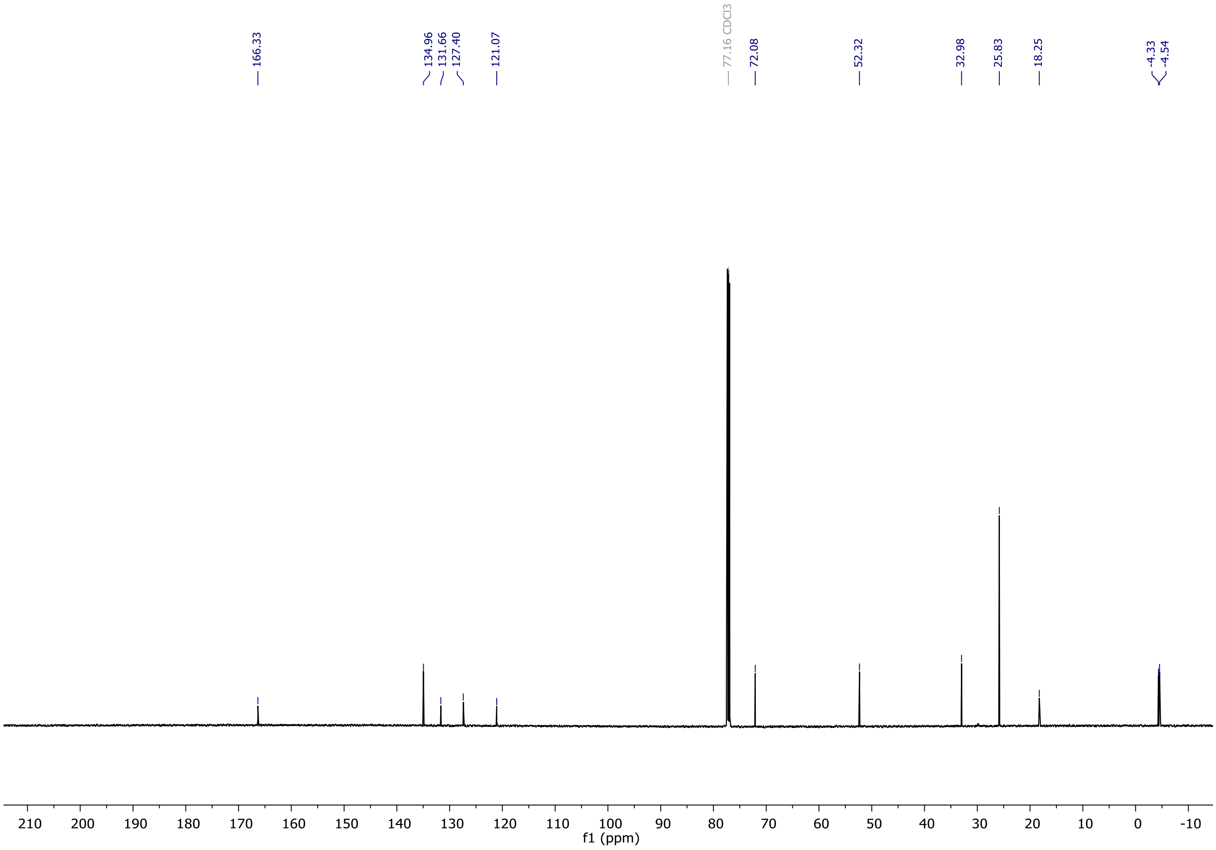

^{1}H NMR (400 MHz, $CDCl_3$, 25 °C) of alcohol **S5**:

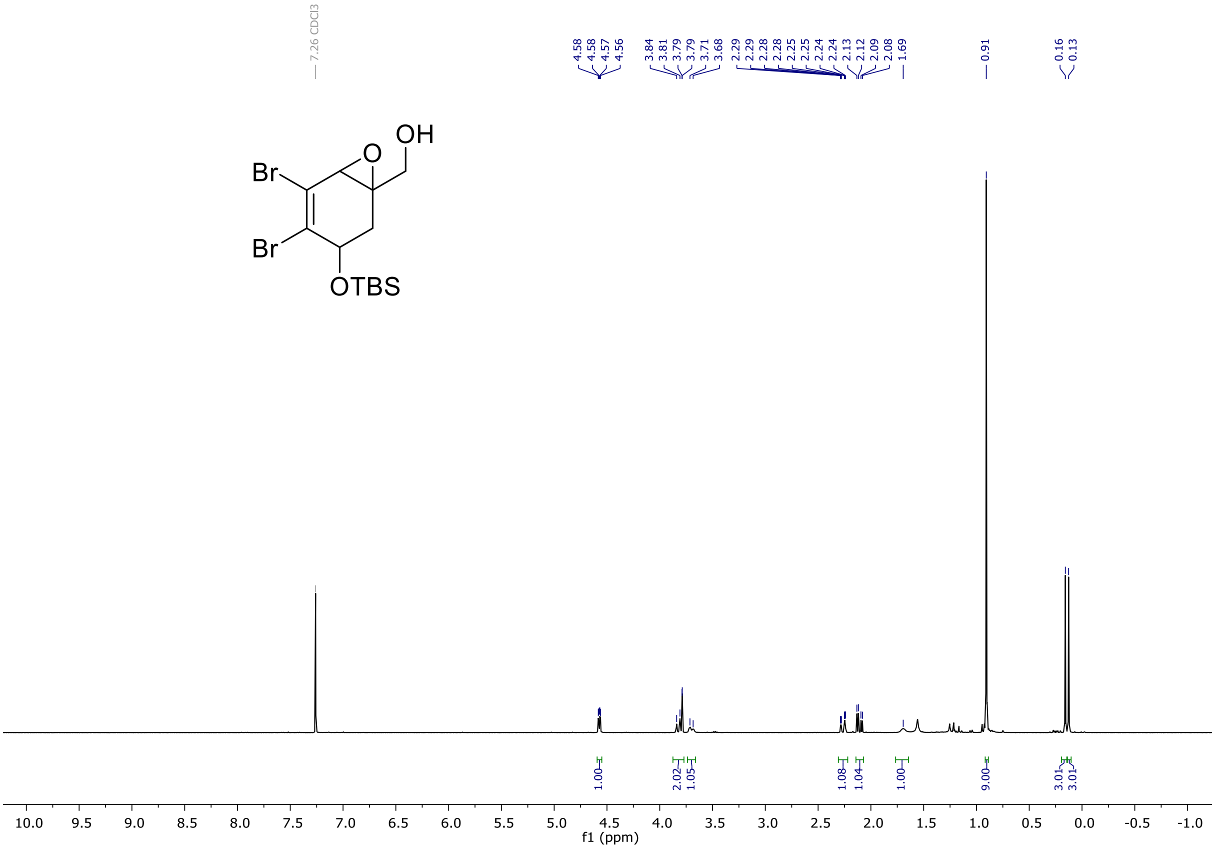

^{13}C NMR (101 MHz, $CDCl_3$, 25 °C) of alcohol **S5**:

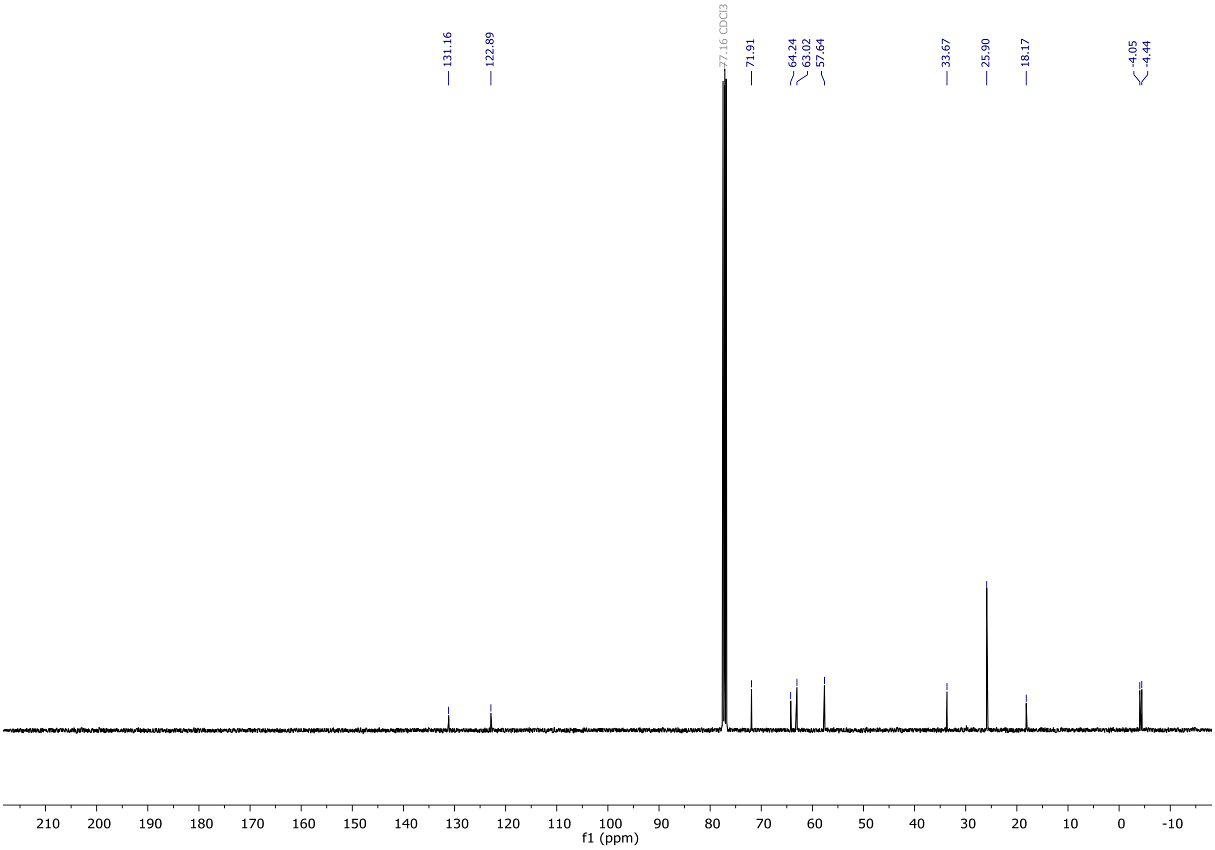

^{1}H NMR (400 MHz, C_6D_6, 25 °C) of diol **5**:

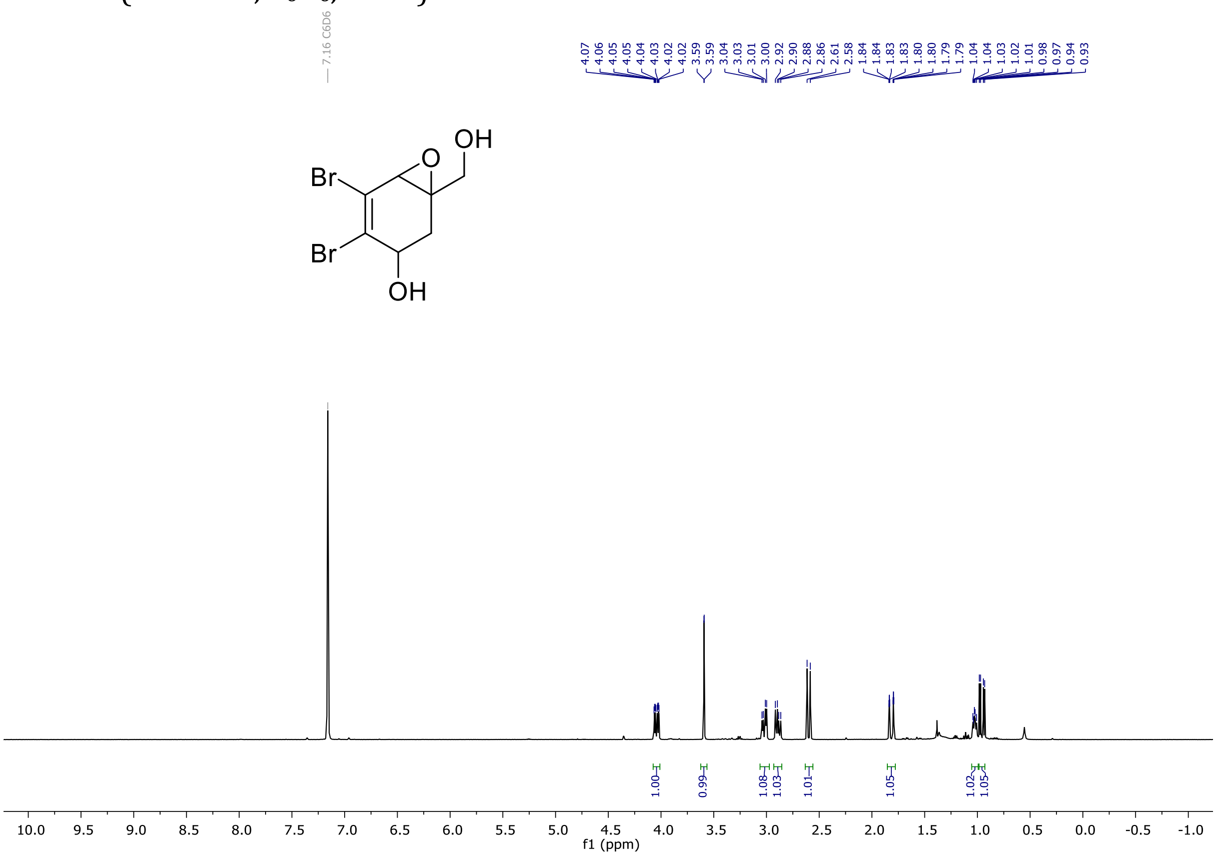

^{13}C NMR (101 MHz, C_6D_6, 25 °C) of diol **5**:

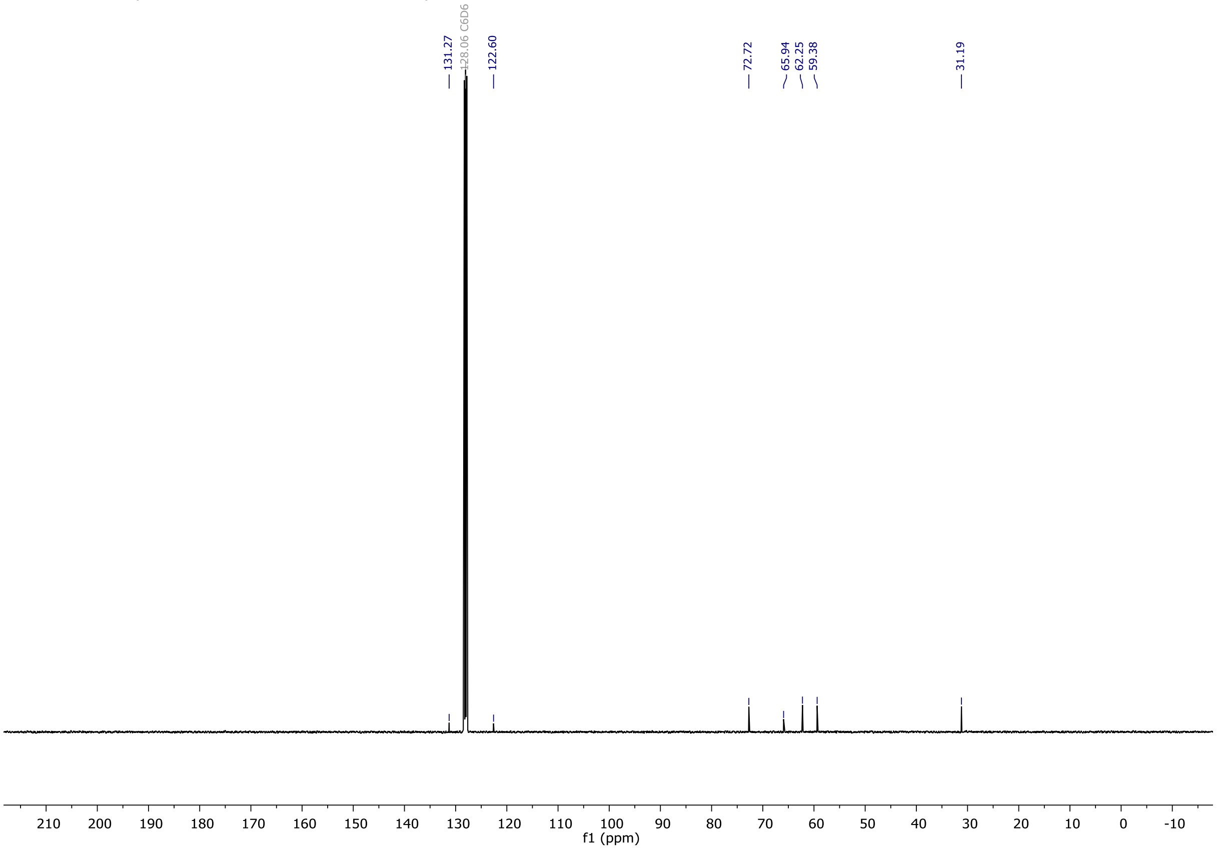

^{1}H NMR (400 MHz, CD_2Cl_2, 25 °C) of aldehyde **7**:

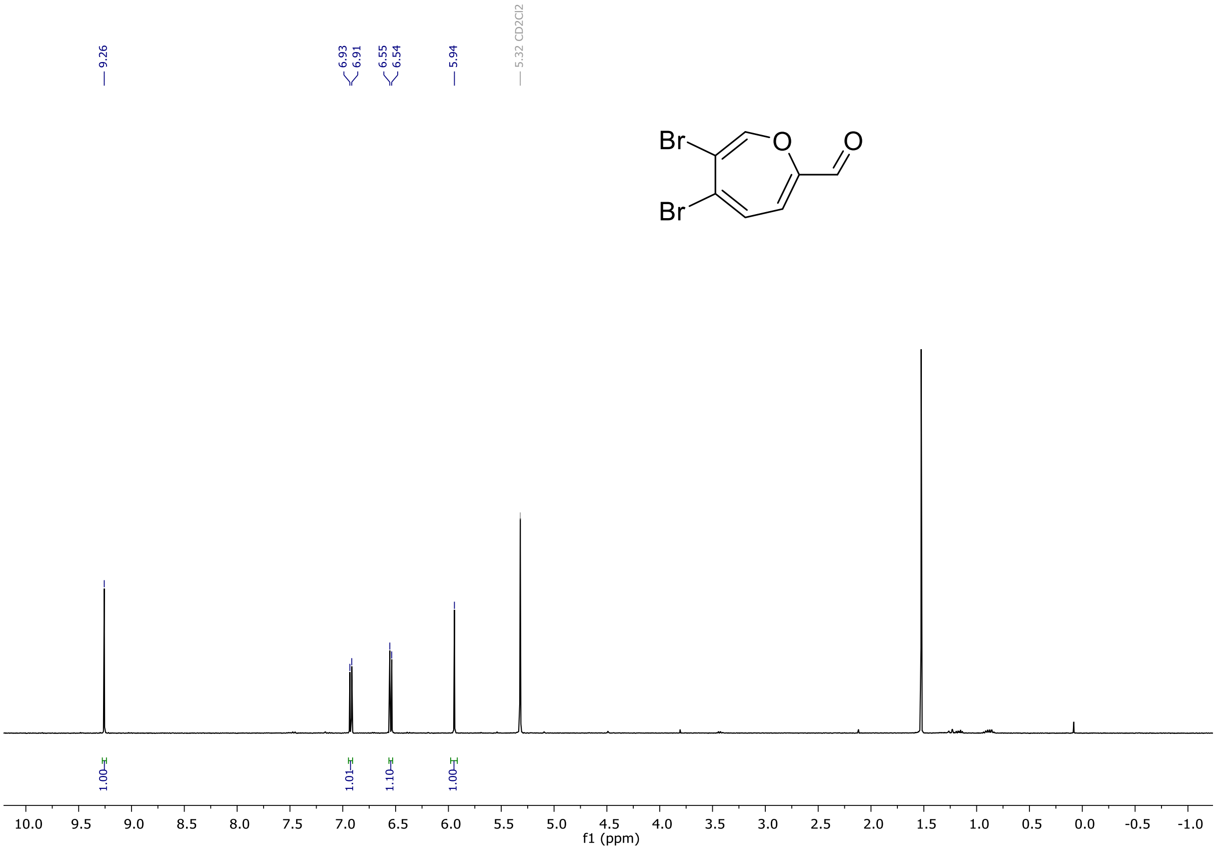

^{13}C NMR (101 MHz, CD_2Cl_2, 25 °C) of aldehyde **7**:

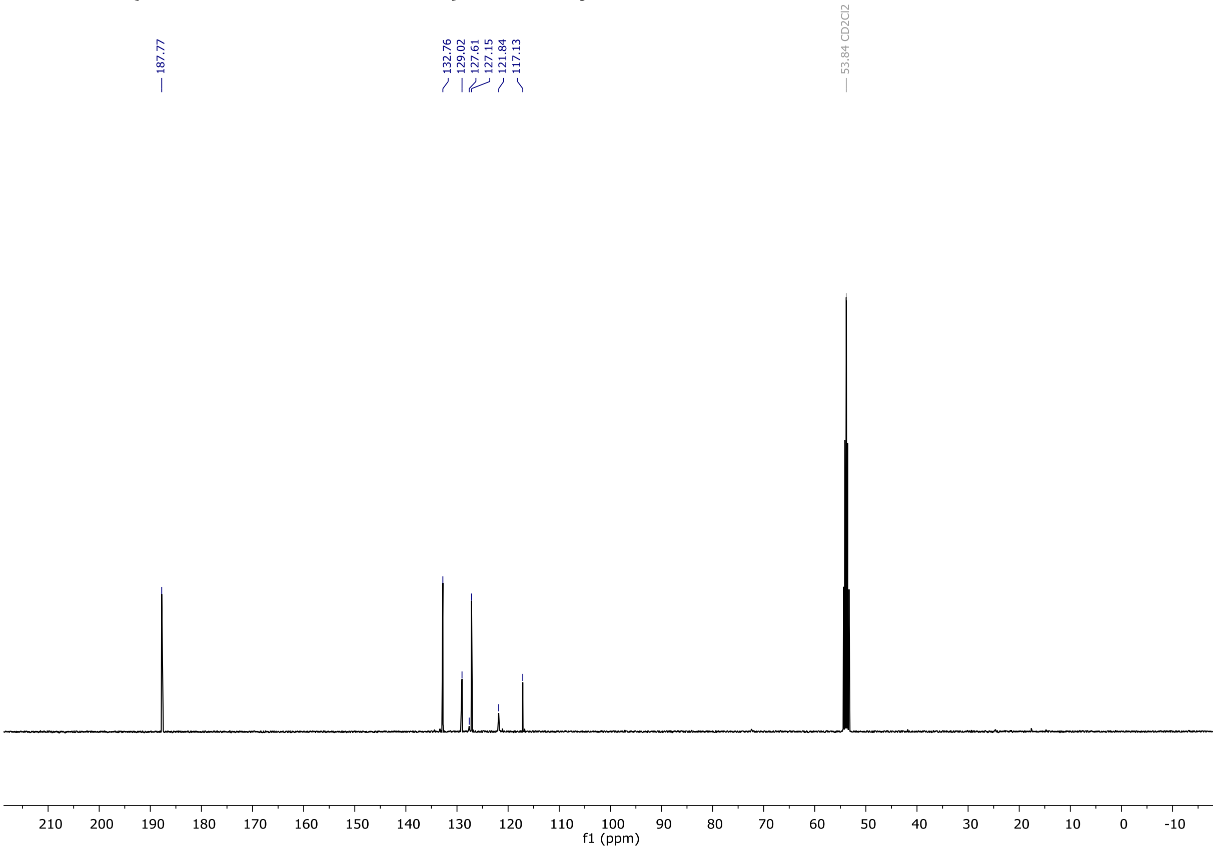

^{1}H NMR (300 MHz, $CDCl_3$, 25 °C) of ester **9**:

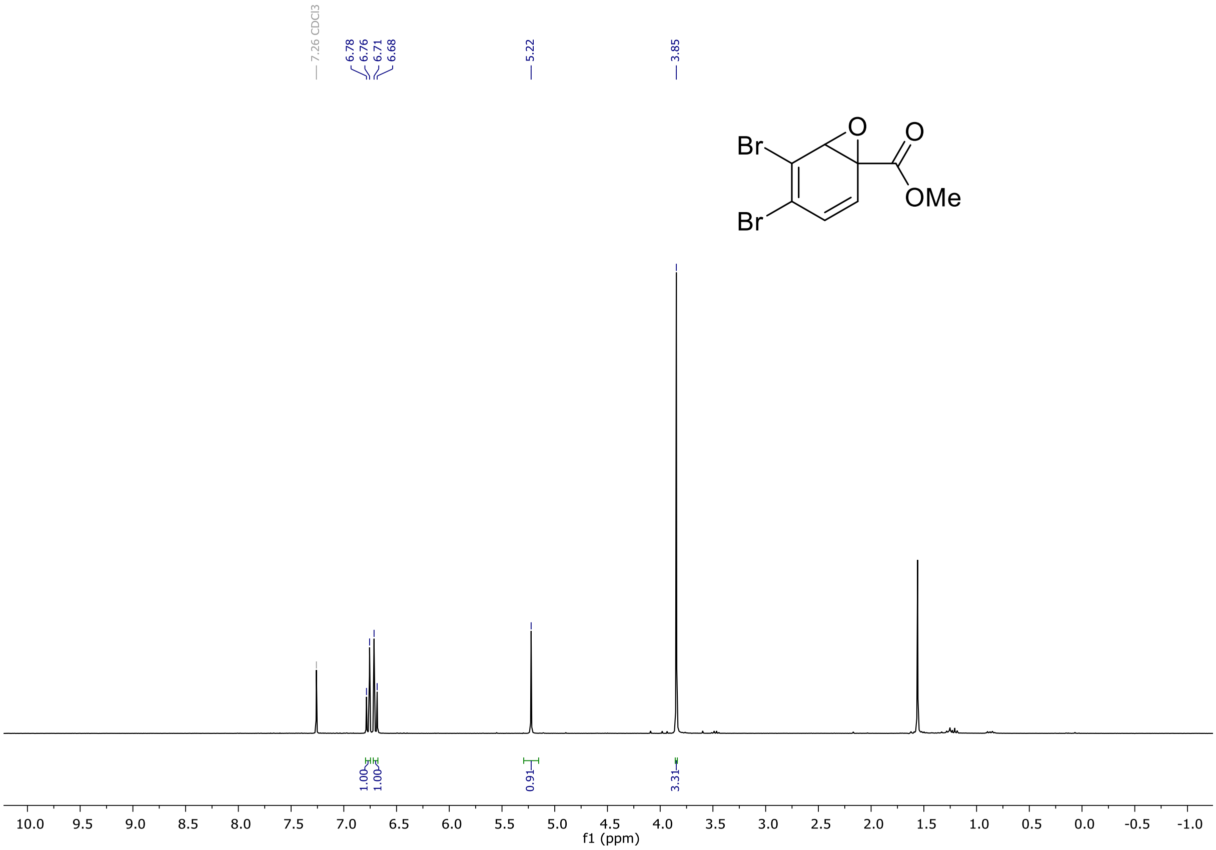

^{13}C NMR (75 MHz, $CDCl_3$, 25 °C) of ester **9**:

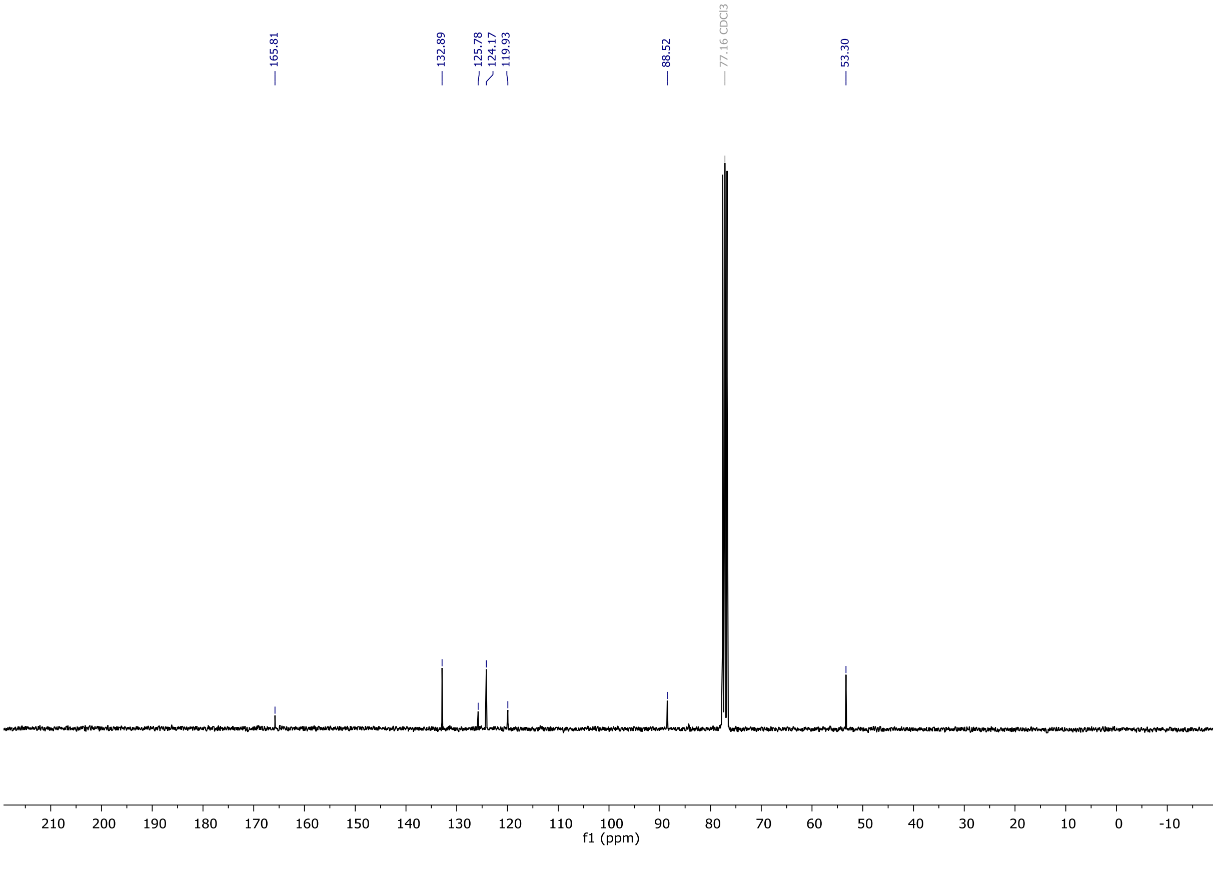

^{1}H NMR (400 MHz, $CDCl_3$, 25 °C) of dienone **S7**:

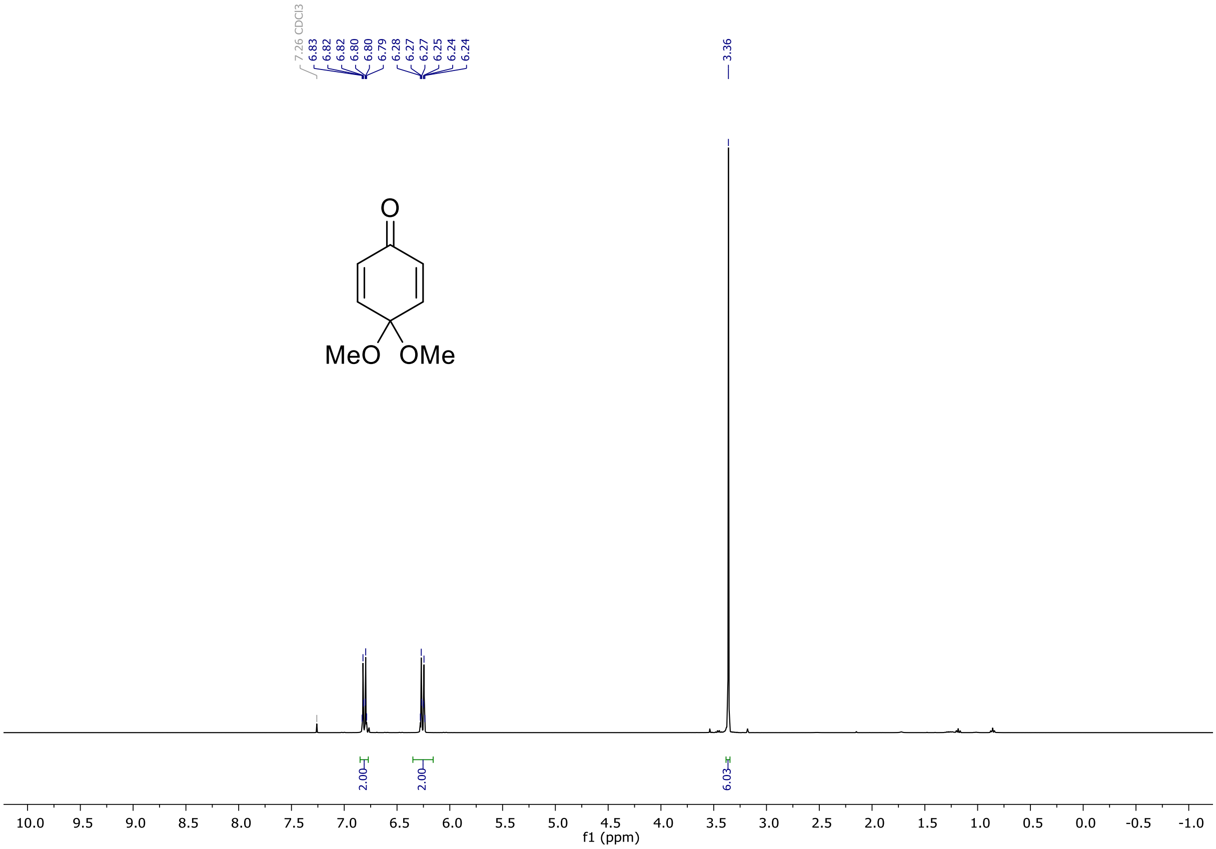

^{1}H NMR (400 MHz, $CDCl_3$, 25 °C) of ketone **S8**:

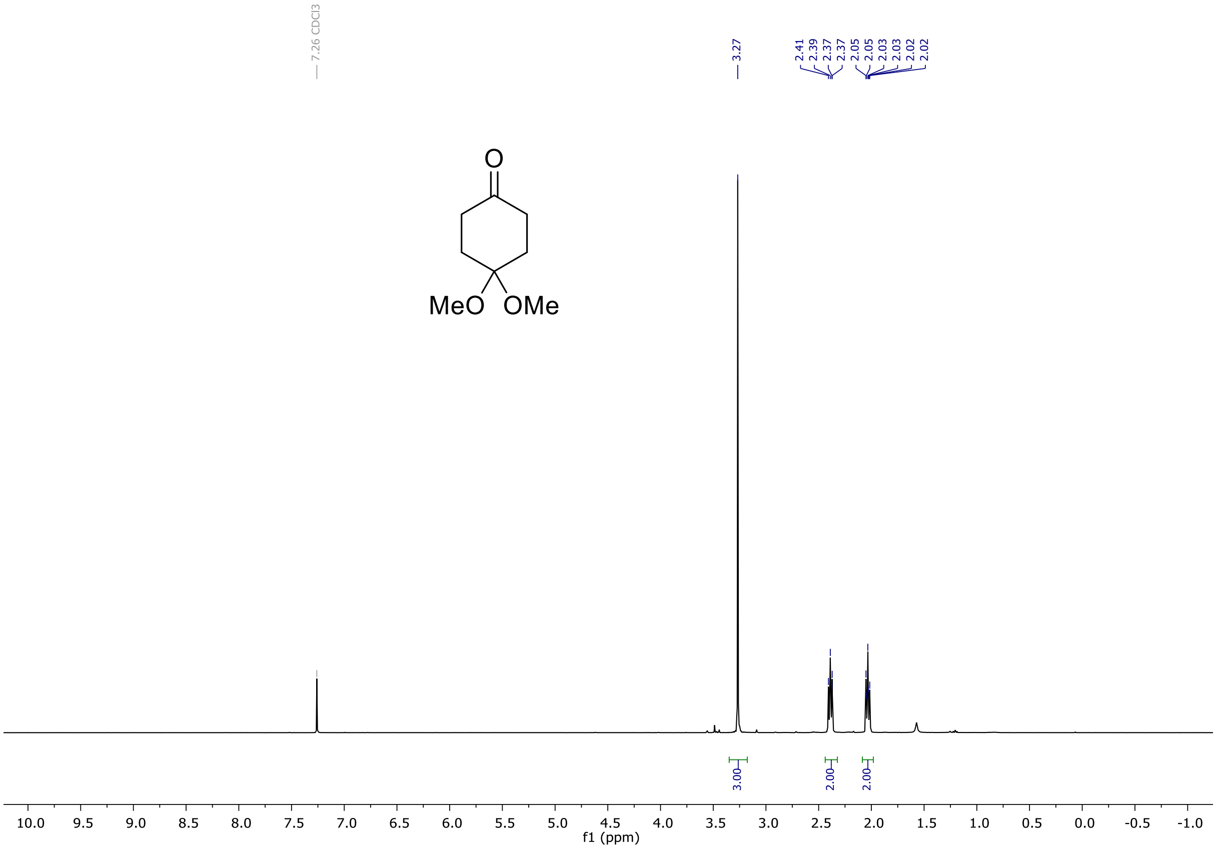

^{1}H NMR (400 MHz, $CDCl_3$, 25 °C) of lactone **S9**:

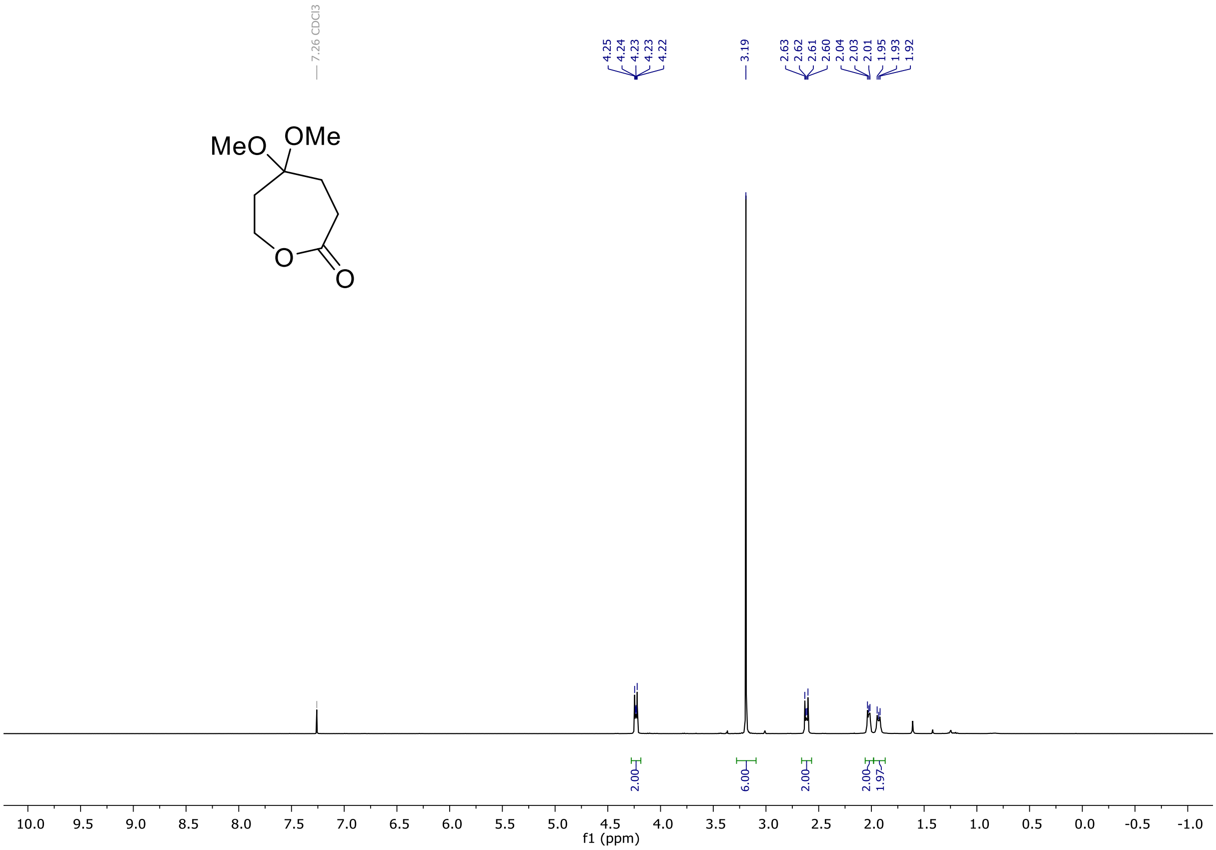

^{13}C NMR (101 MHz, $CDCl_3$, 25 °C) of lactone **S9**:

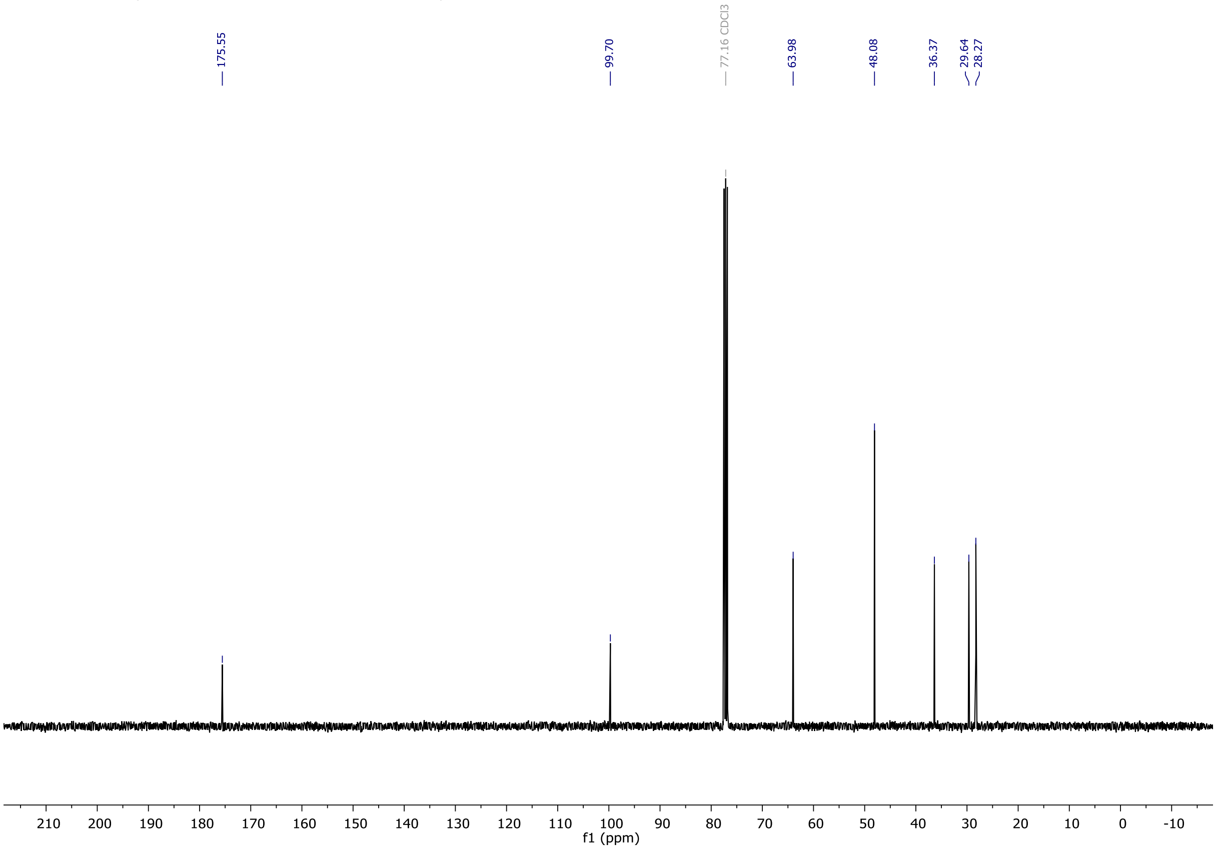

^{1}H NMR (400 MHz, $CDCl_3$, 25 °C) of enol ether **S10**:

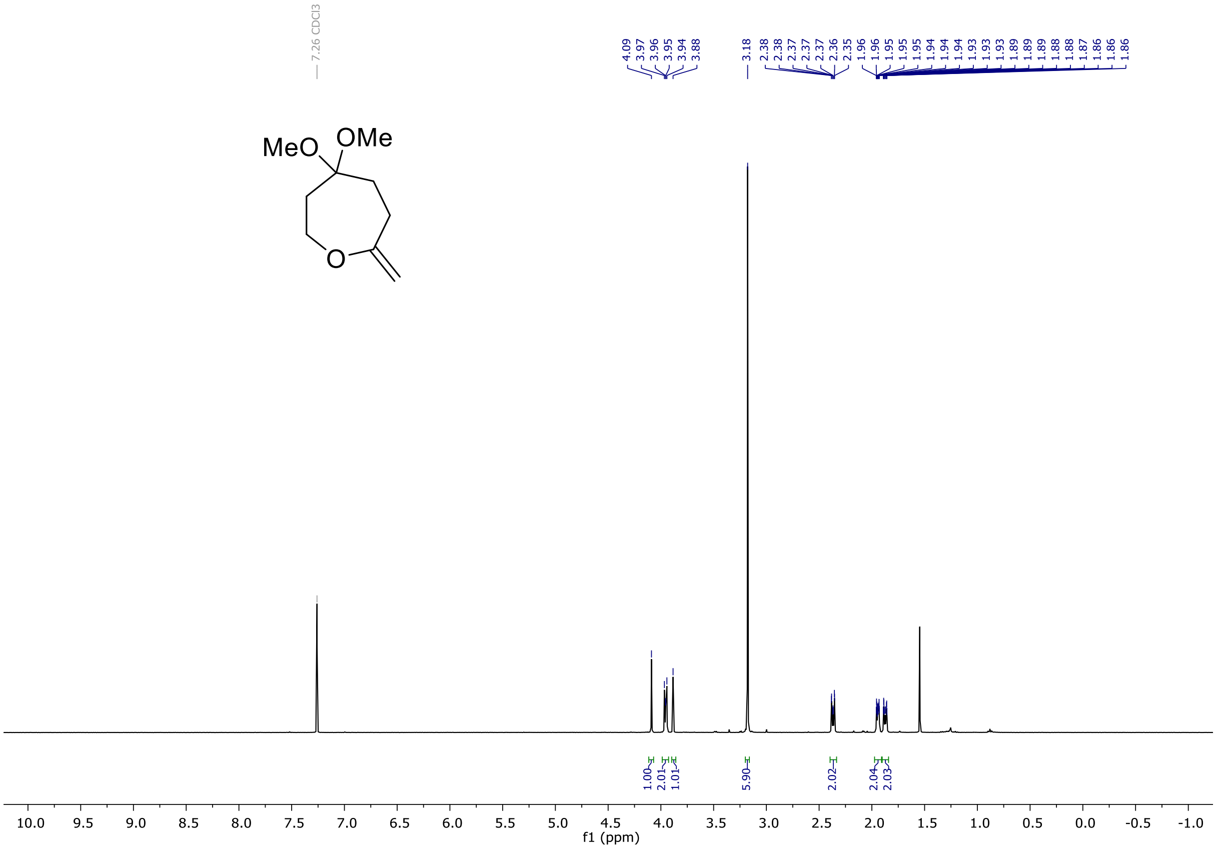

^{13}C NMR (101 MHz, $CDCl_3$, 25 °C) of enol ether **S10**:

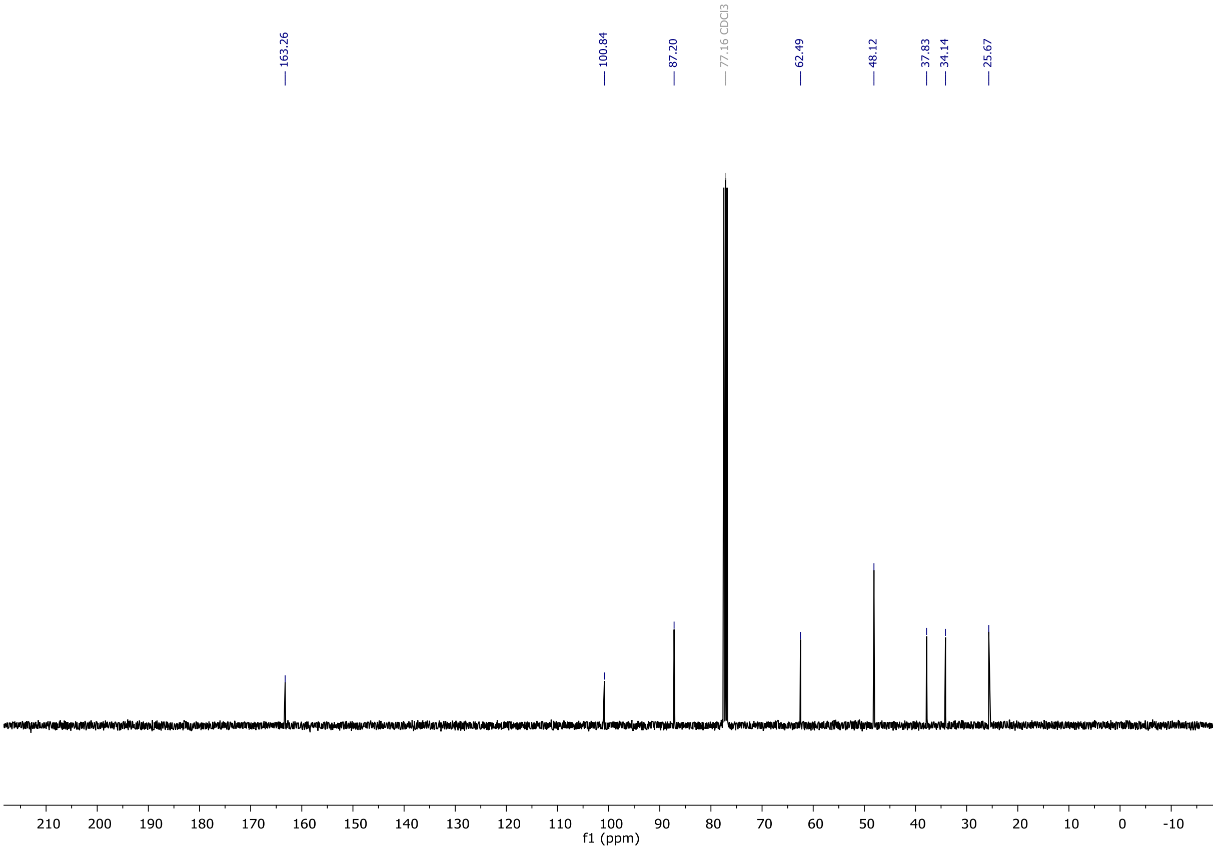

^{1}H NMR (400 MHz, $CDCl_3$, 25 °C) of ester **10**:

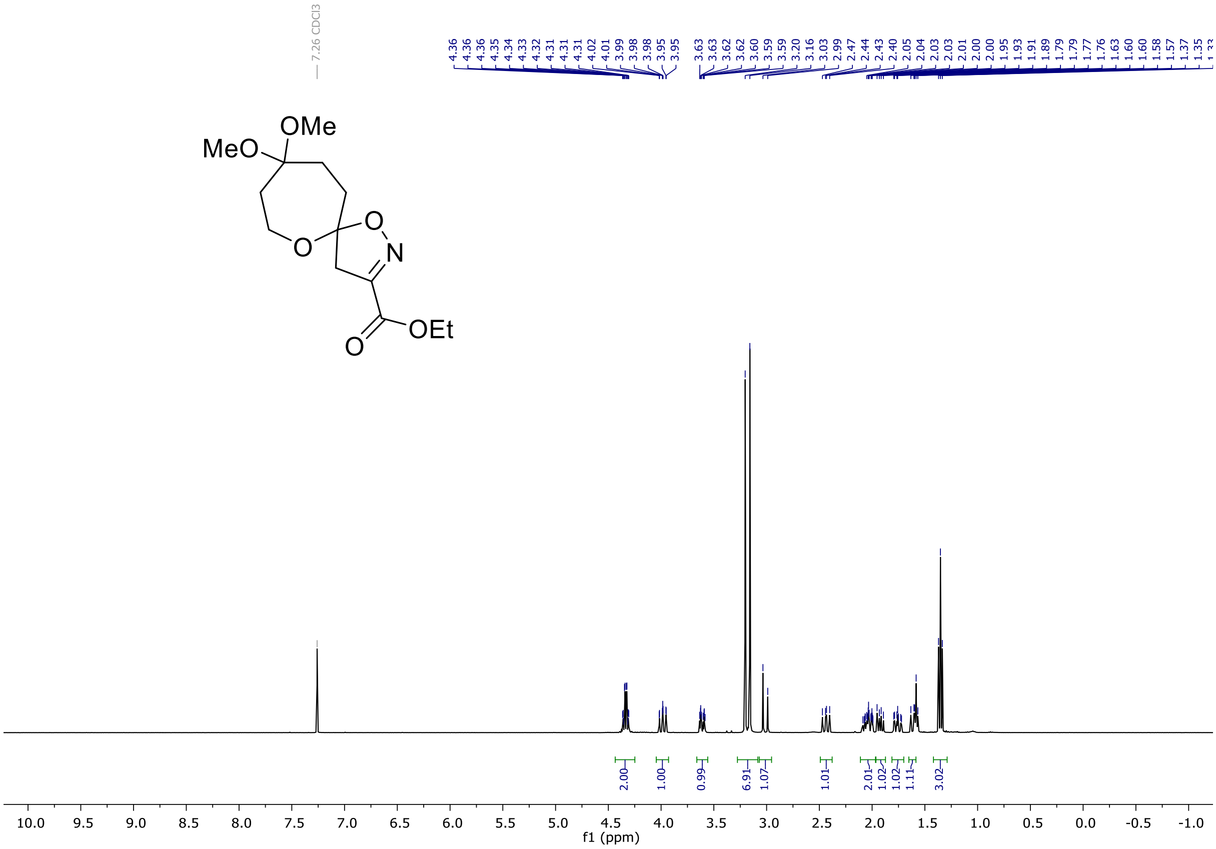

^{13}C NMR (101 MHz, $CDCl_3$, 25 °C) of ester **10**:

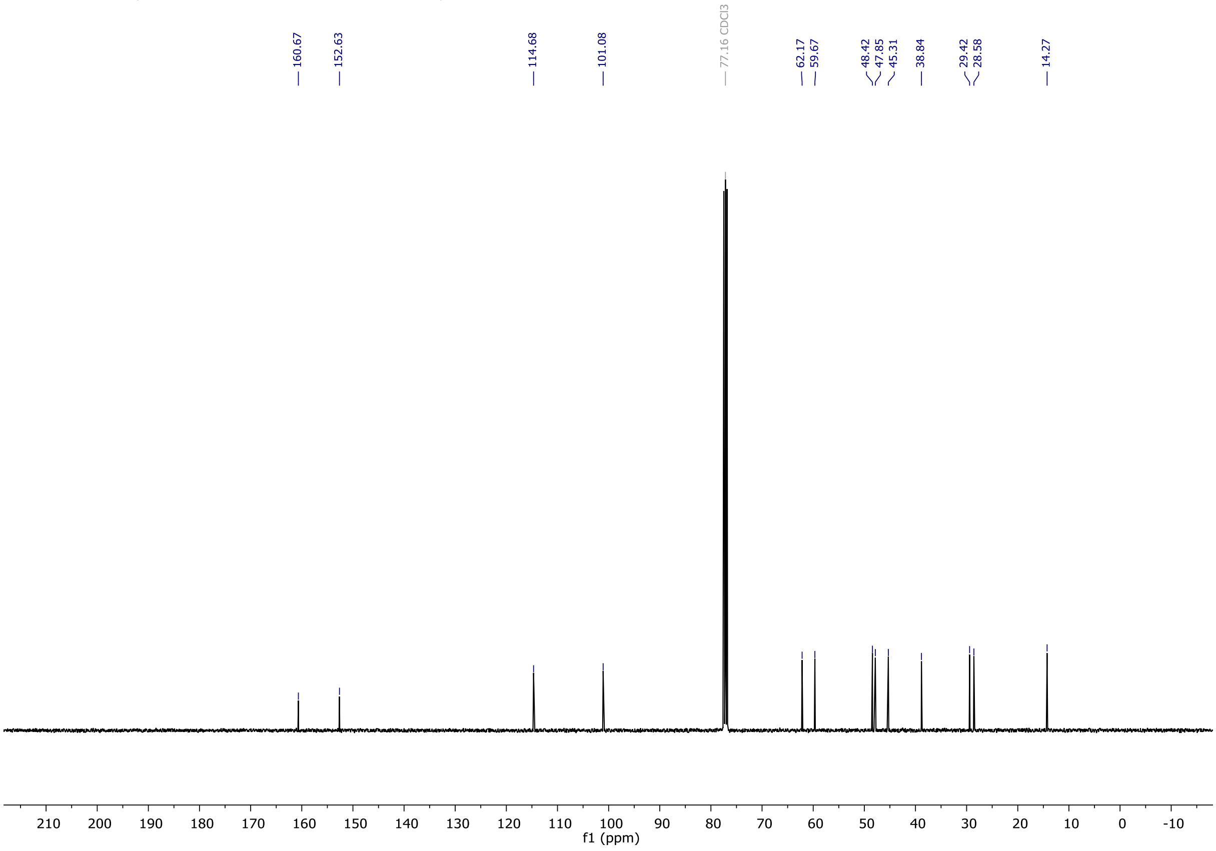

^{1}H NMR (400 MHz, $CDCl_3$, 25 °C) of ester **12**:

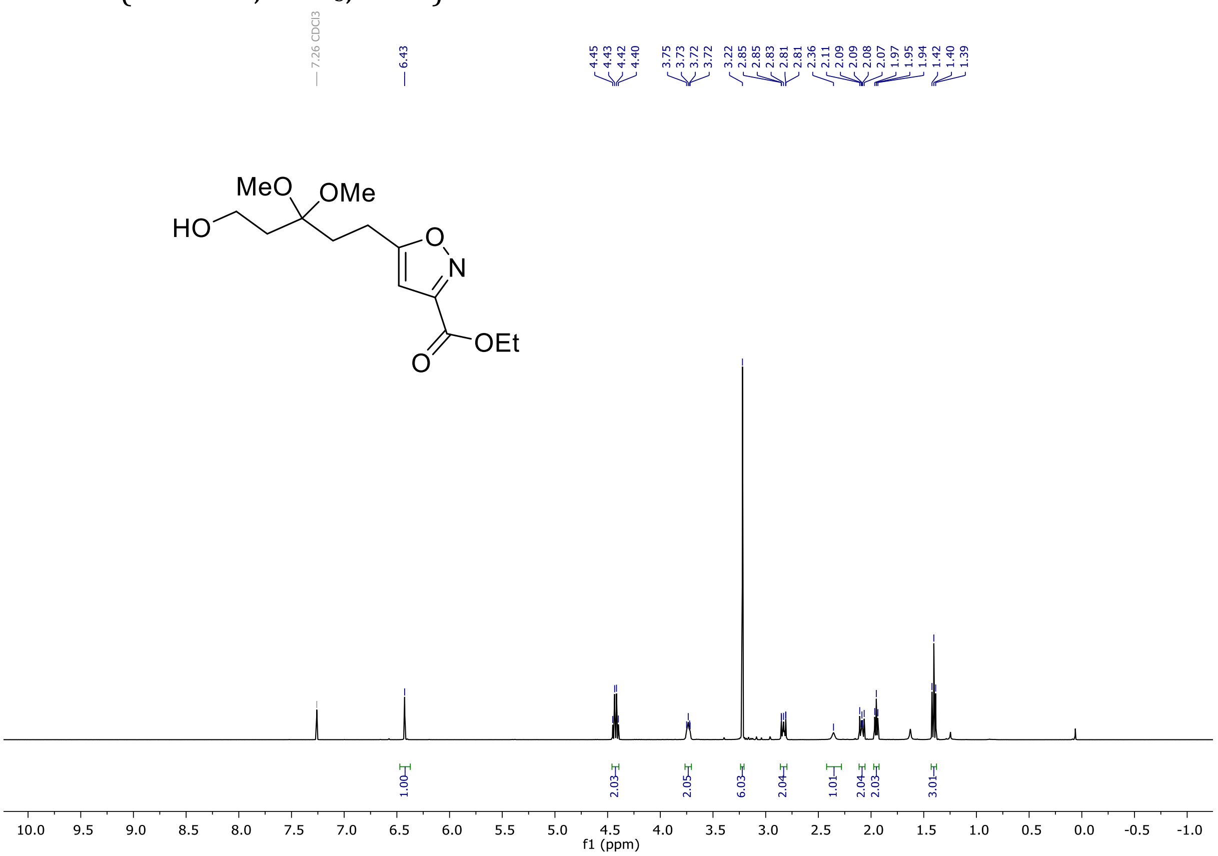

^{13}C NMR (101 MHz, $CDCl_3$, 25 °C) of ester **12**:

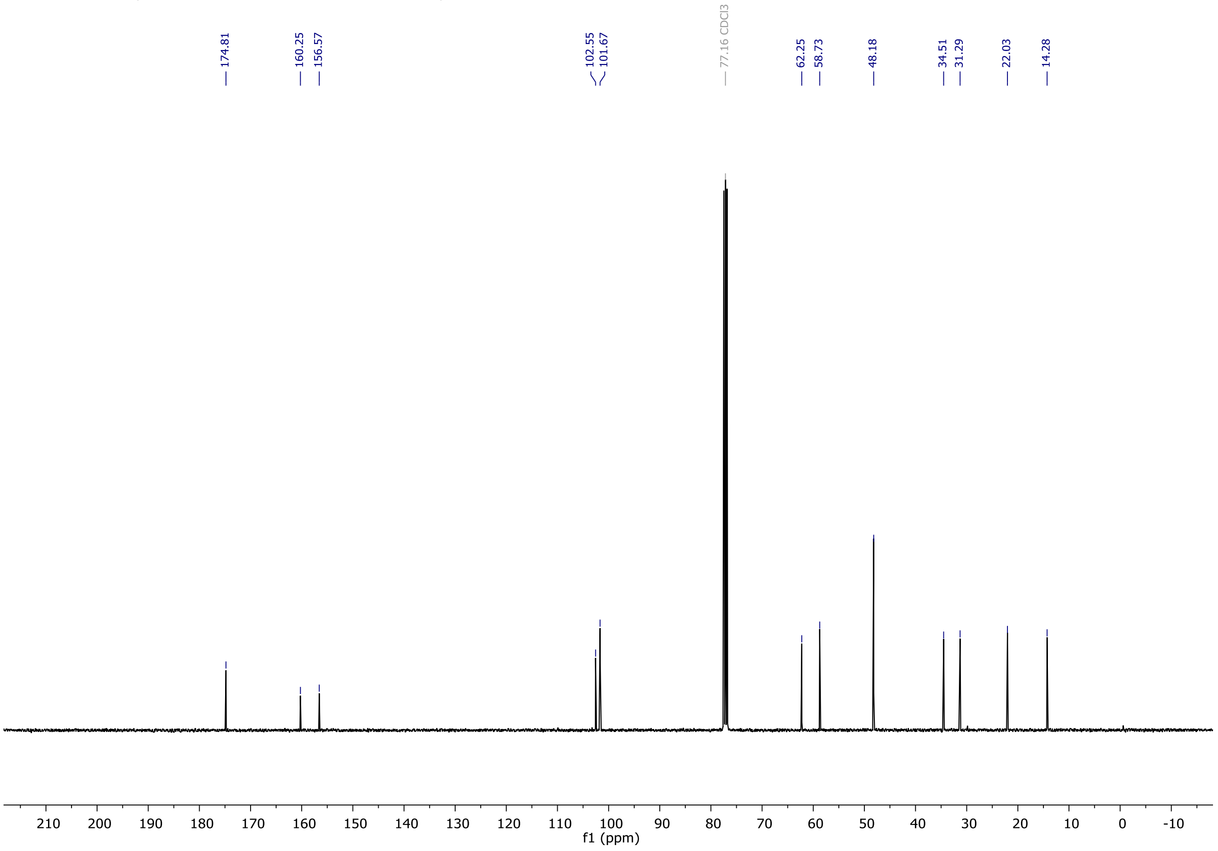

^{1}H NMR (400 MHz, $CDCl_3$, 25 °C) of aldehyde **S13**:

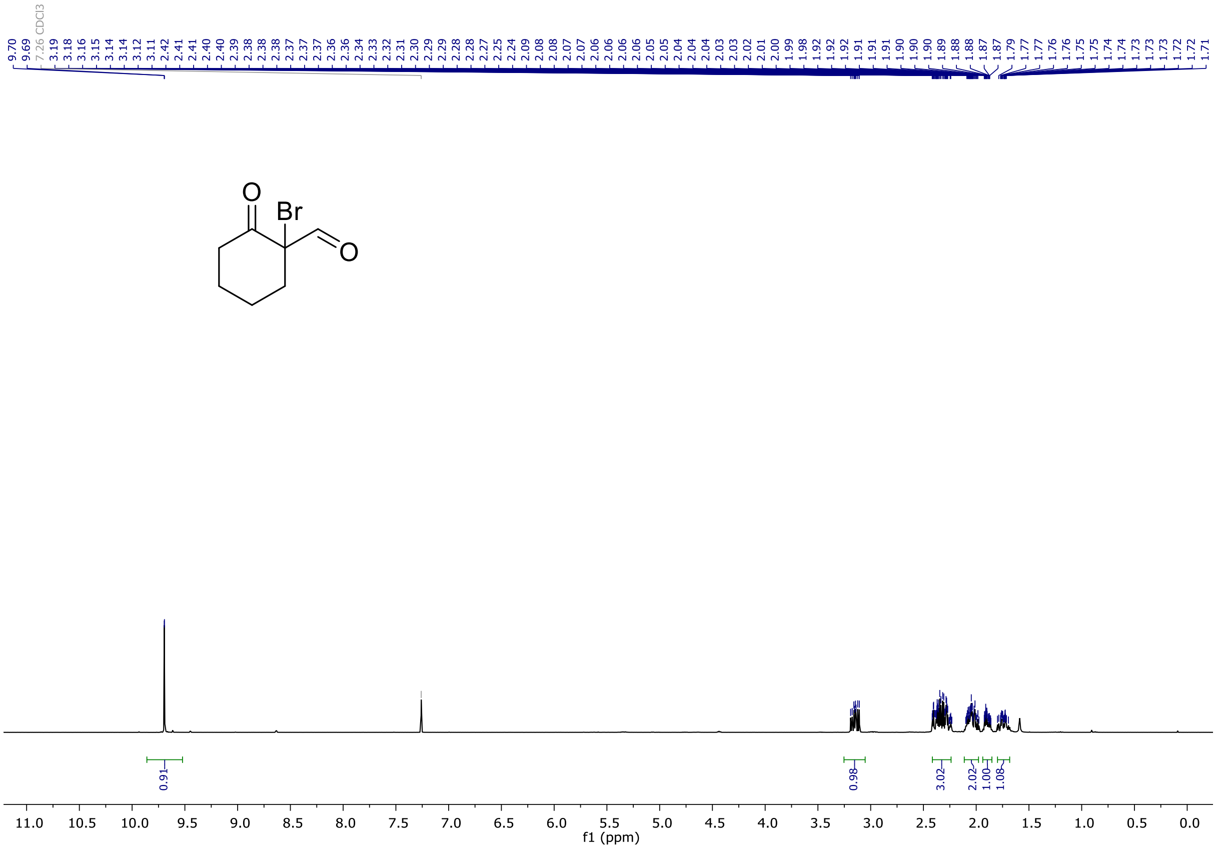

^{13}C NMR (101 MHz, $CDCl_3$, 25 °C) of aldehyde **S13**:

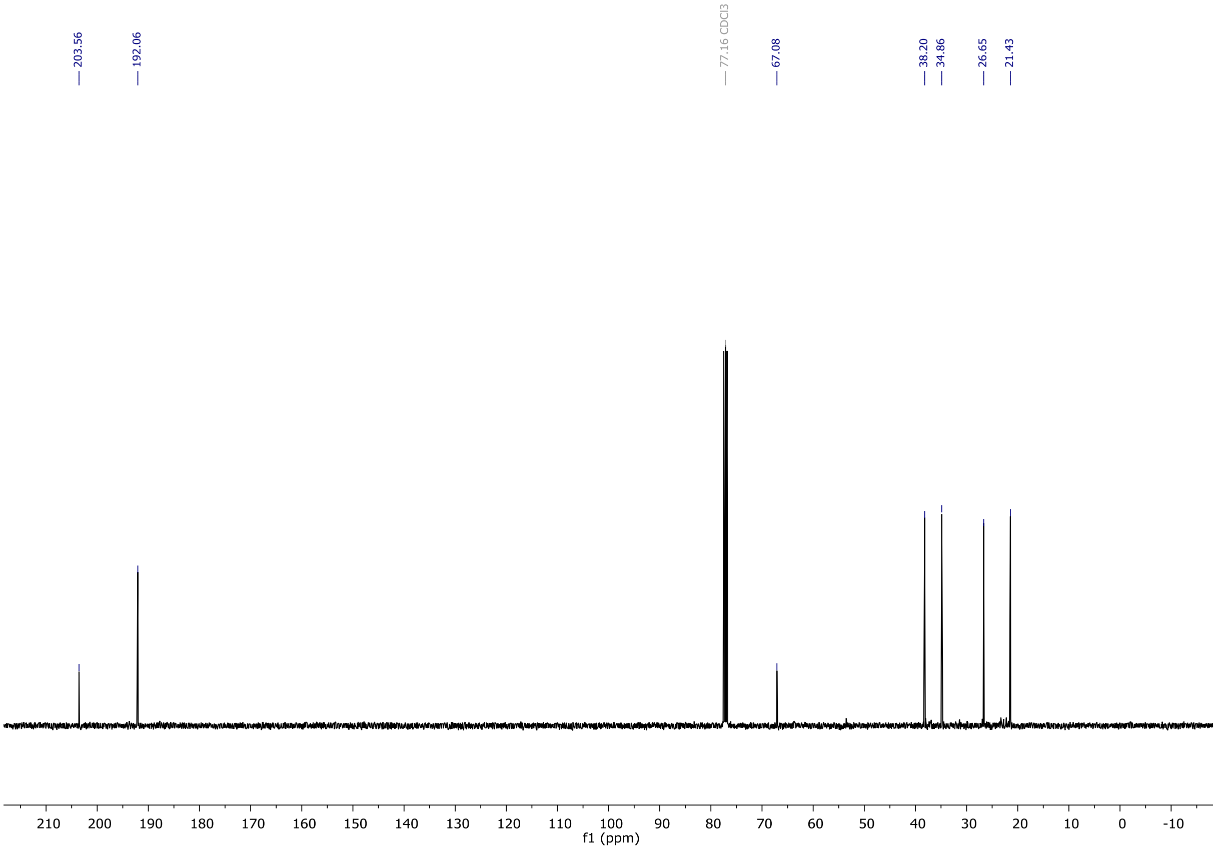

^{1}H NMR (400 MHz, $CDCl_3$, 25 °C) of nitronate **16a**:

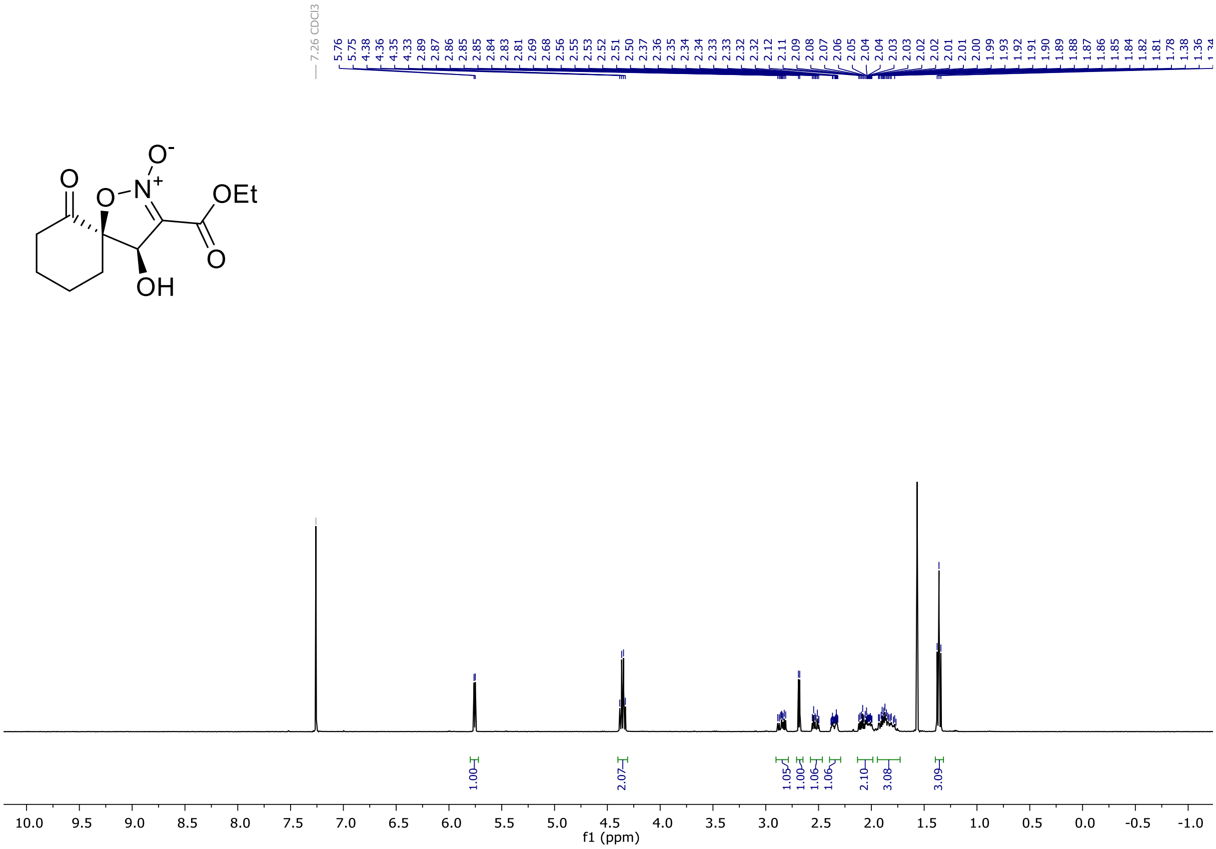

^{13}C NMR (101 MHz, $CDCl_3$, 25 °C) of nitronate **16a**:

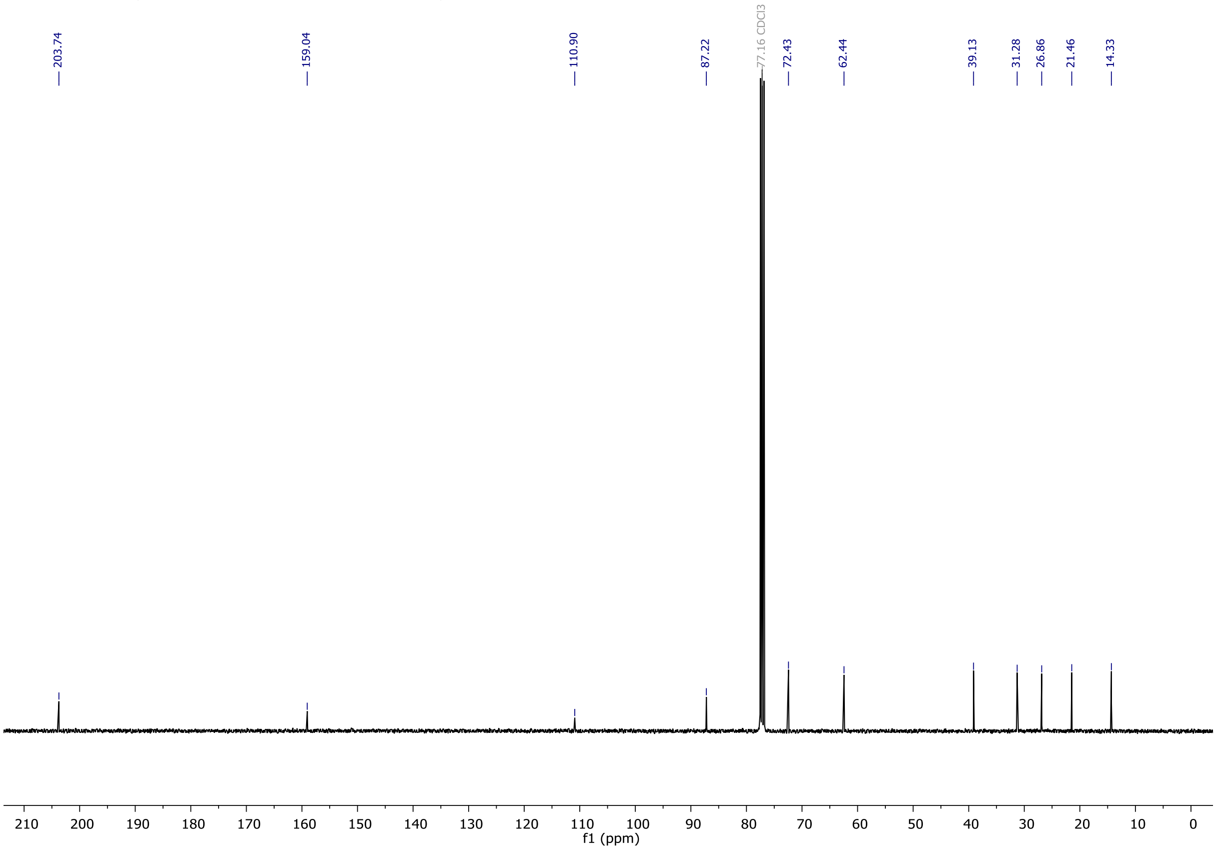

^{1}H NMR (400 MHz, DMSO-*d*$_6$, 25 °C) of nitronate **16b**:

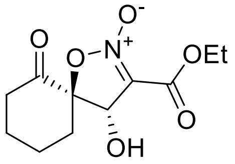

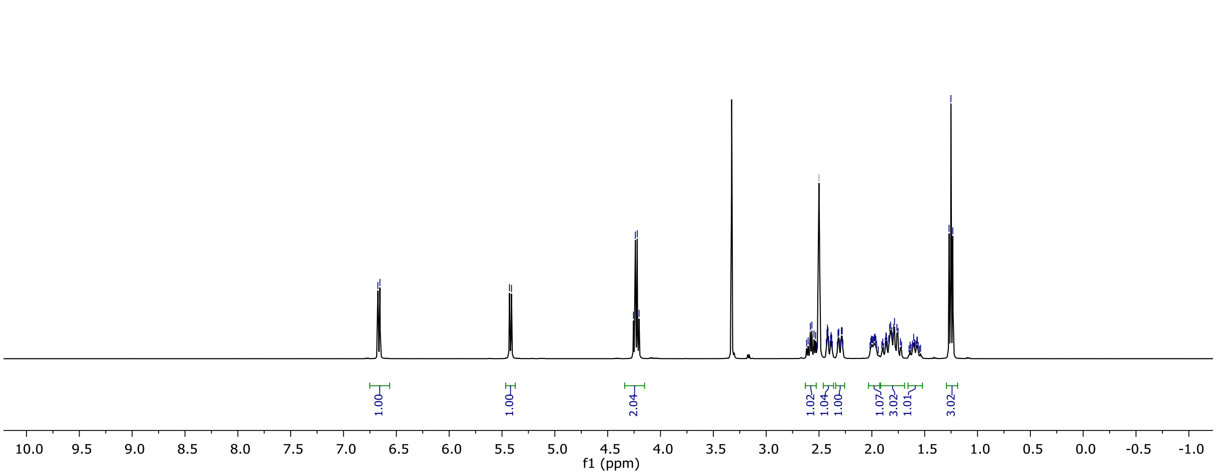

^{13}C NMR (101 MHz, DMSO-*d*$_6$, 25 °C) of nitronate **16b**:

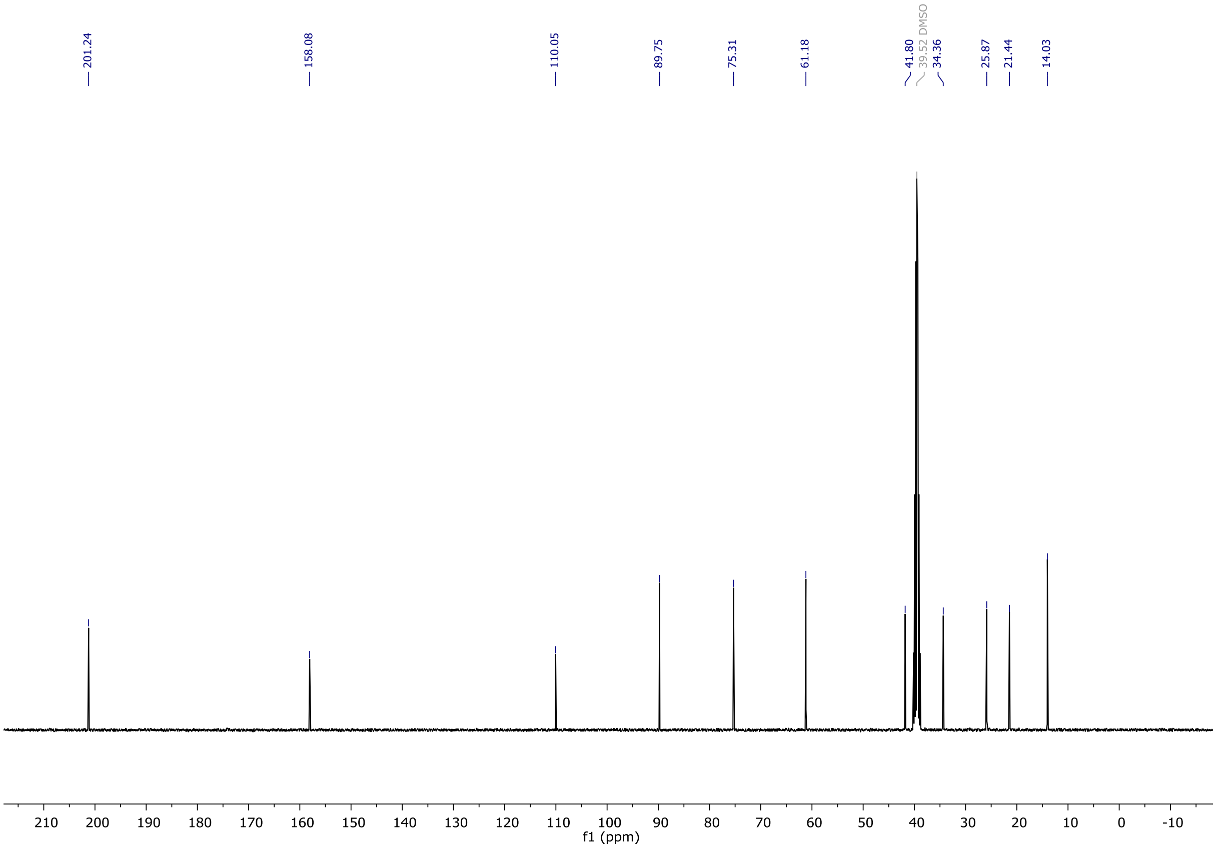

^{1}H NMR (400 MHz, $CDCl_3$, 25 °C) of ester **18**:

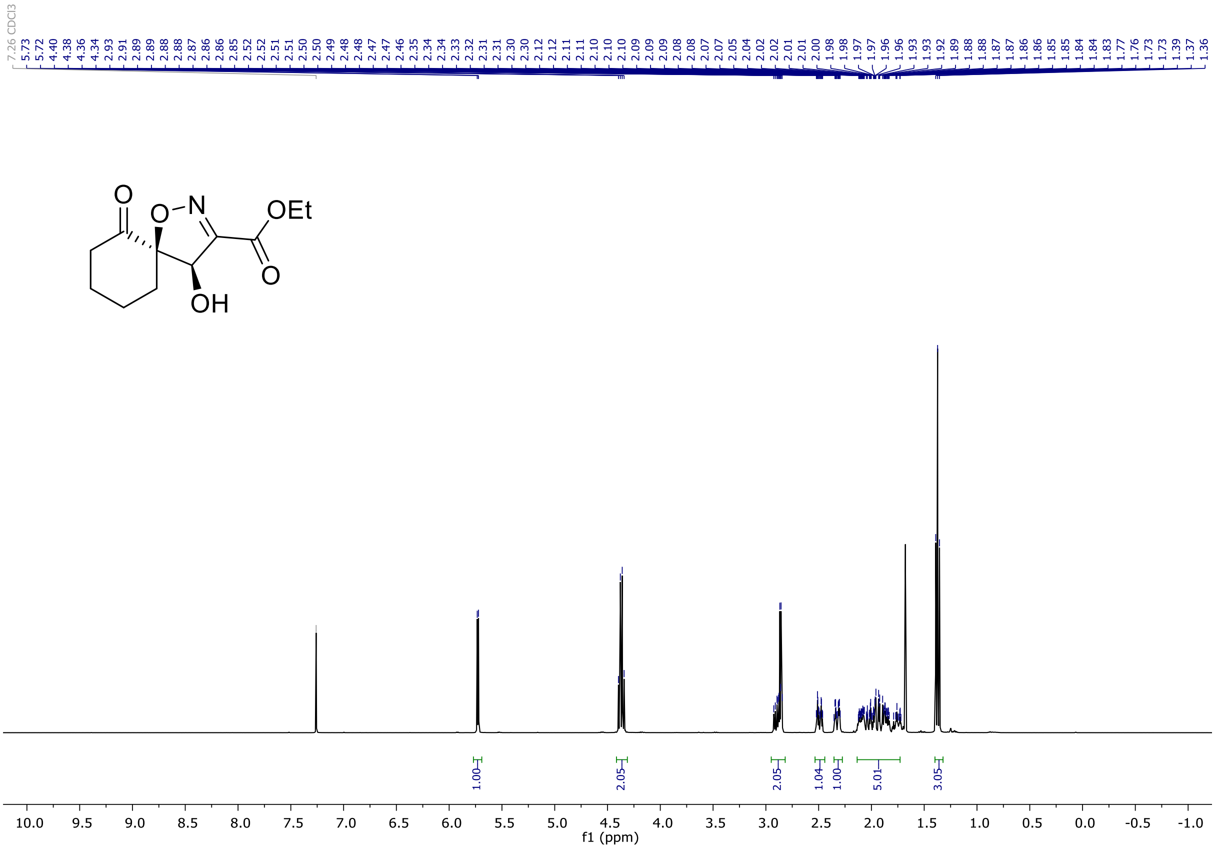

^{13}C NMR (101 MHz, $CDCl_3$, 25 °C) of ester **18**:

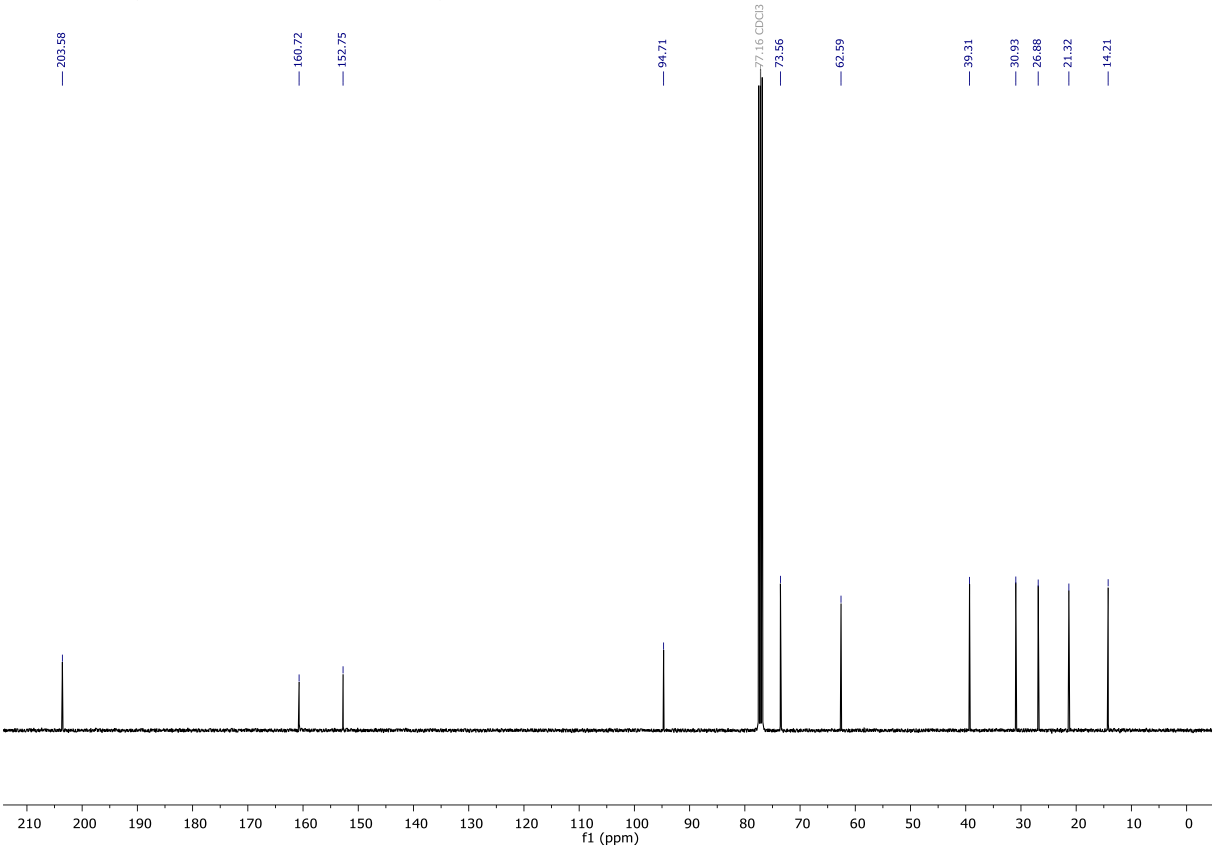

^{1}H NMR (400 MHz, $CDCl_3$, 25 °C) of lactone **19**:

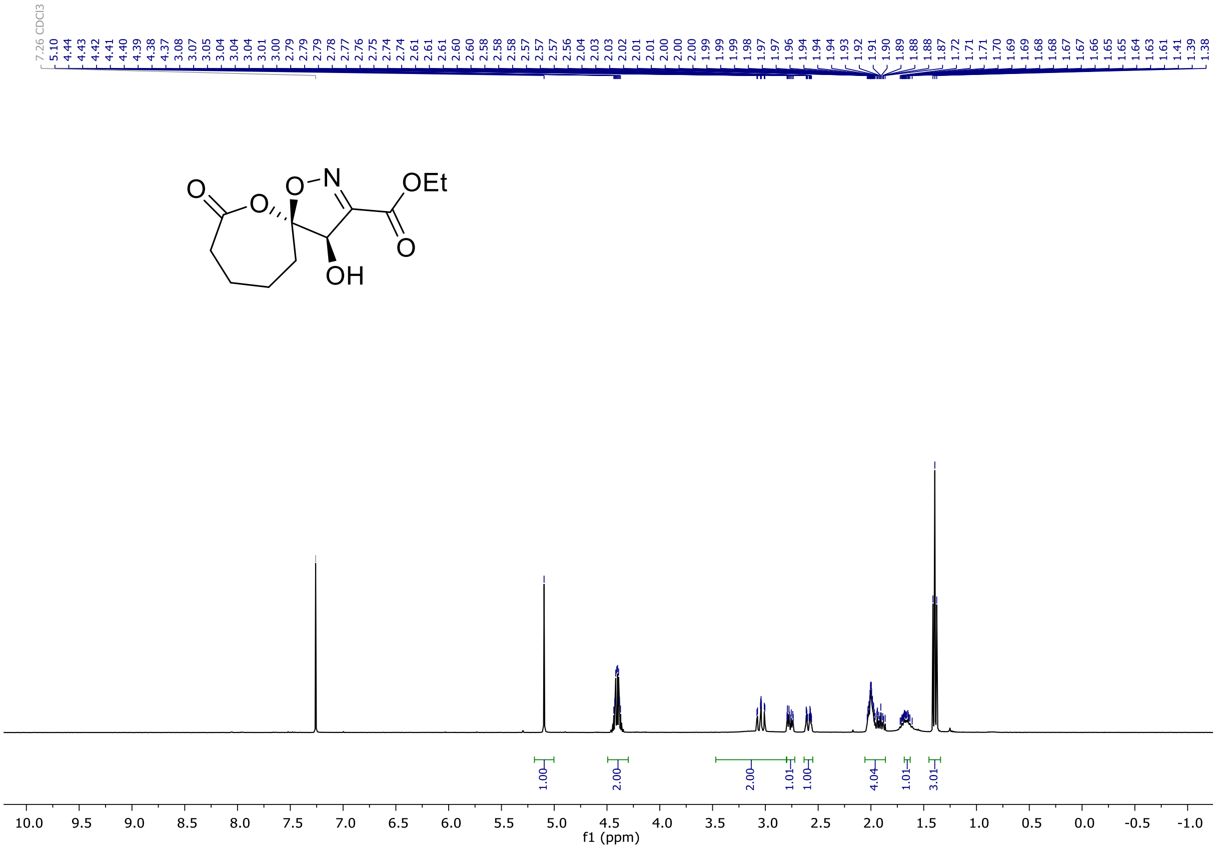

^{13}C NMR (101 MHz, $CDCl_3$, 25 °C) of lactone **19**:

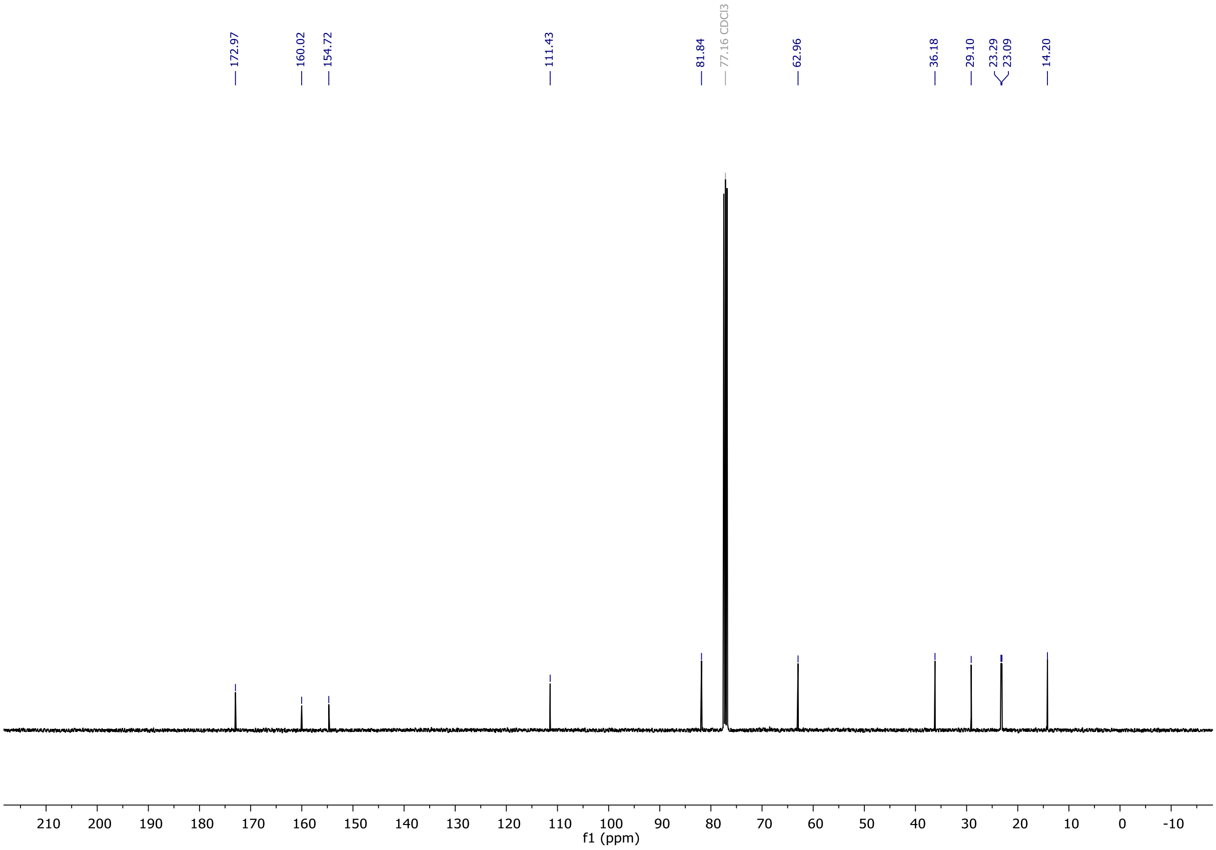

^{1}H NMR (400 MHz, $CDCl_3$, 25 °C) of carbonate **20**:

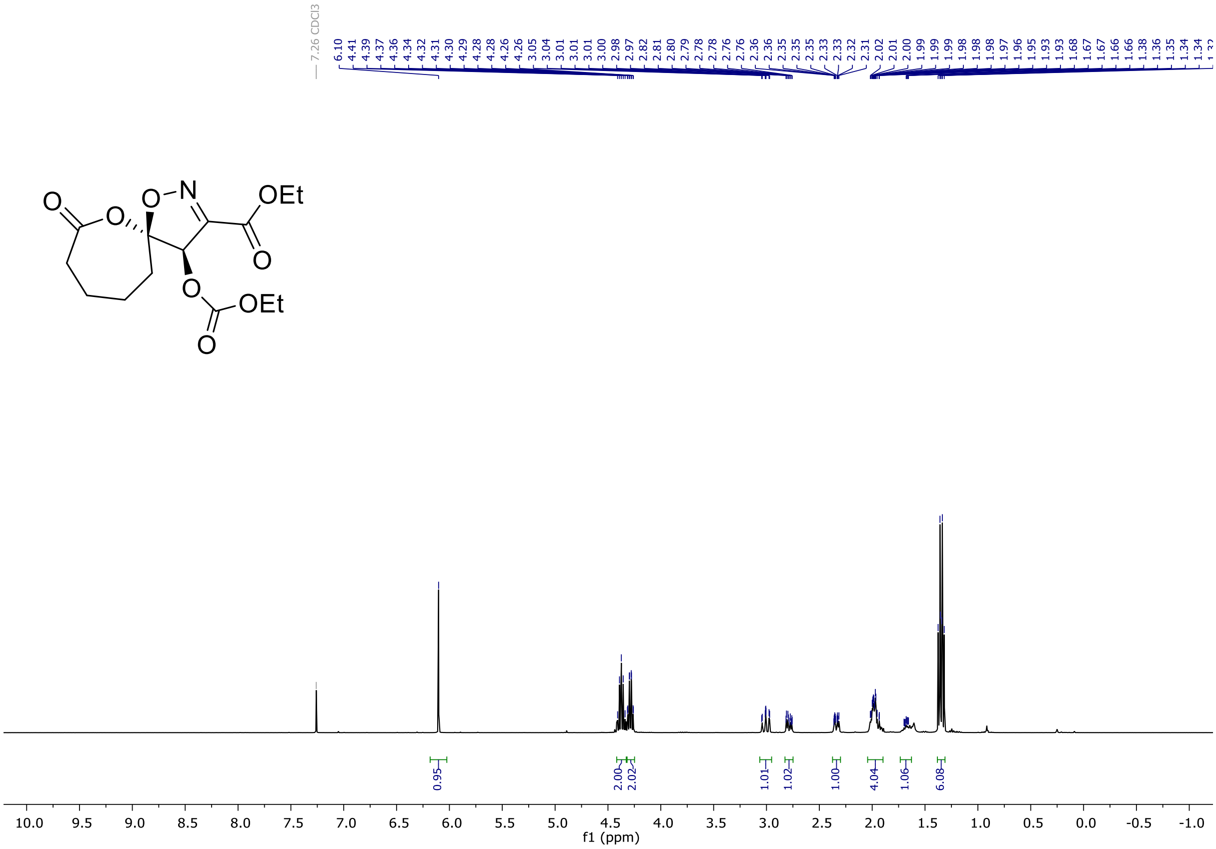

^{13}C NMR (101 MHz, $CDCl_3$, 25 °C) of carbonate **20**:

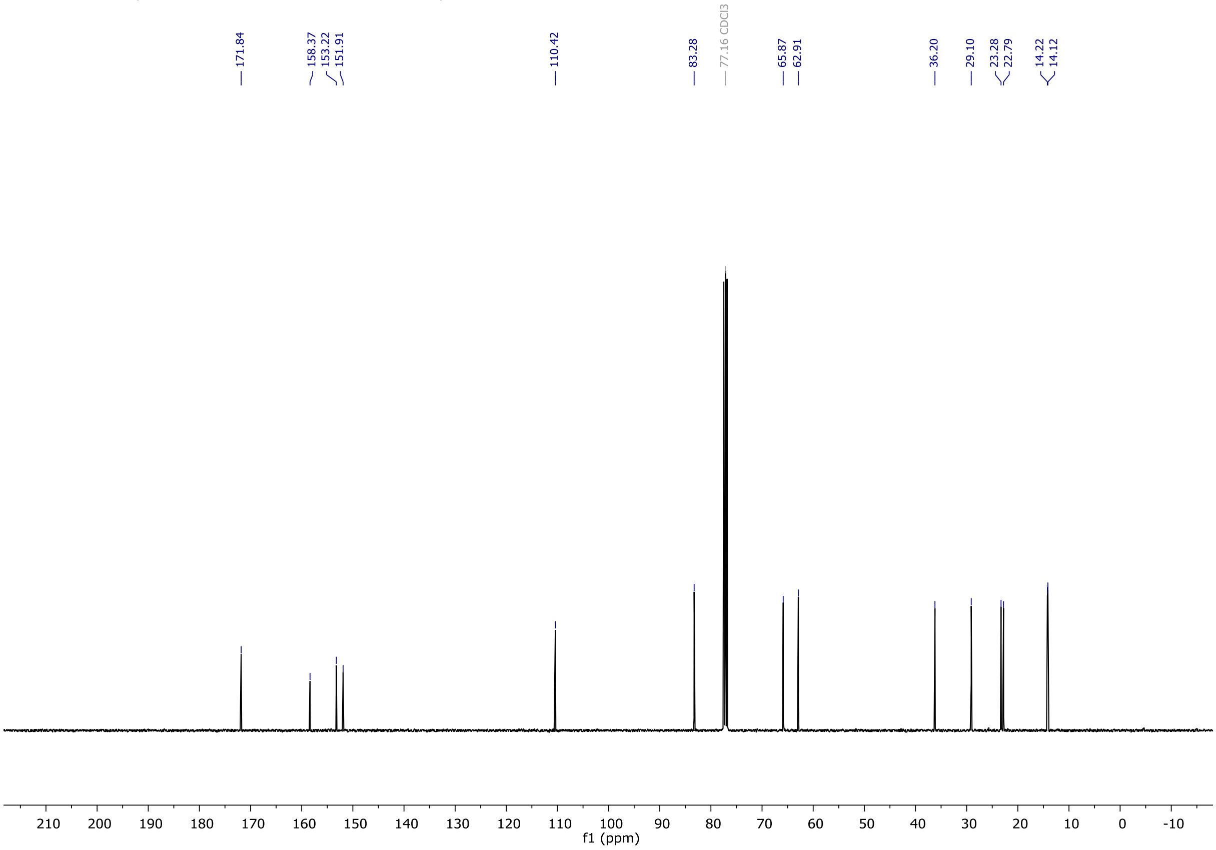

^{1}H NMR (400 MHz, $CDCl_3$, 25 °C) of ester **21**:

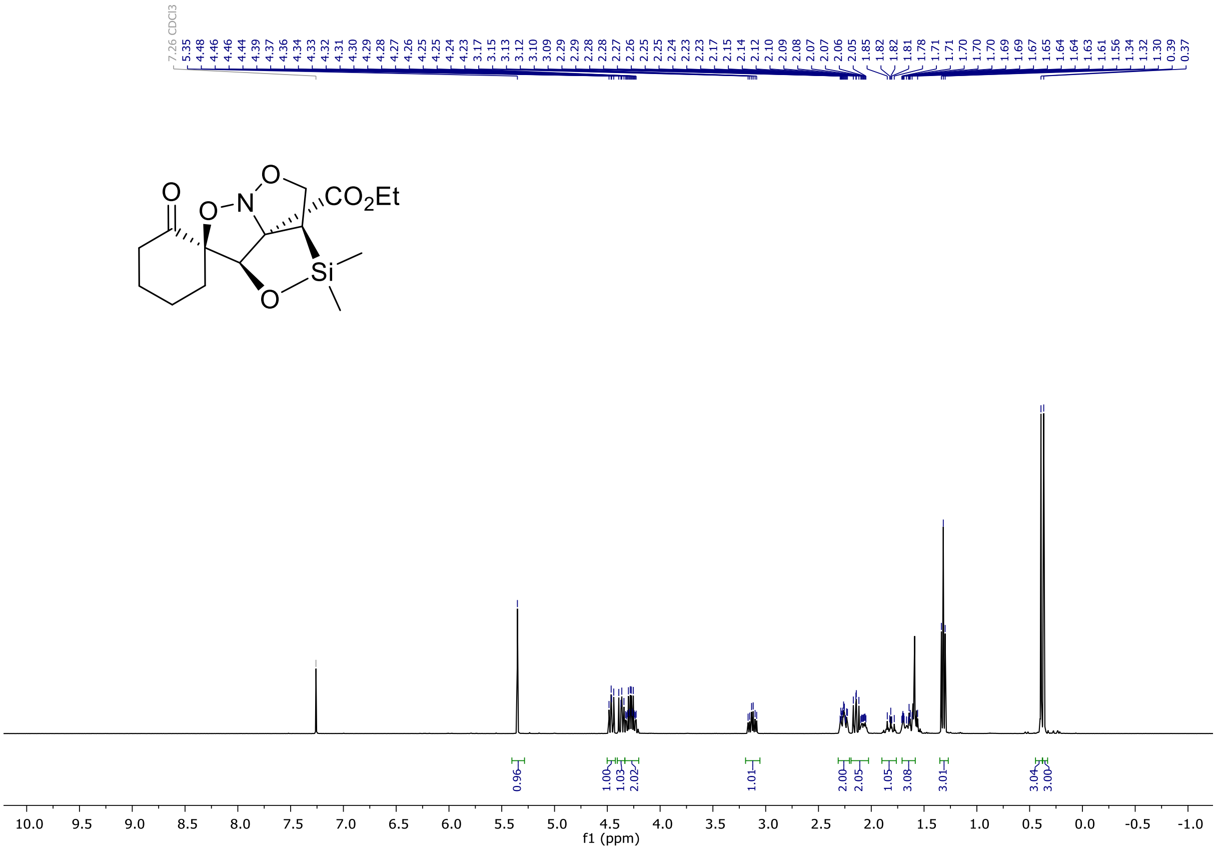

^{13}C NMR (101 MHz, $CDCl_3$, 25 °C) of ester **21**:

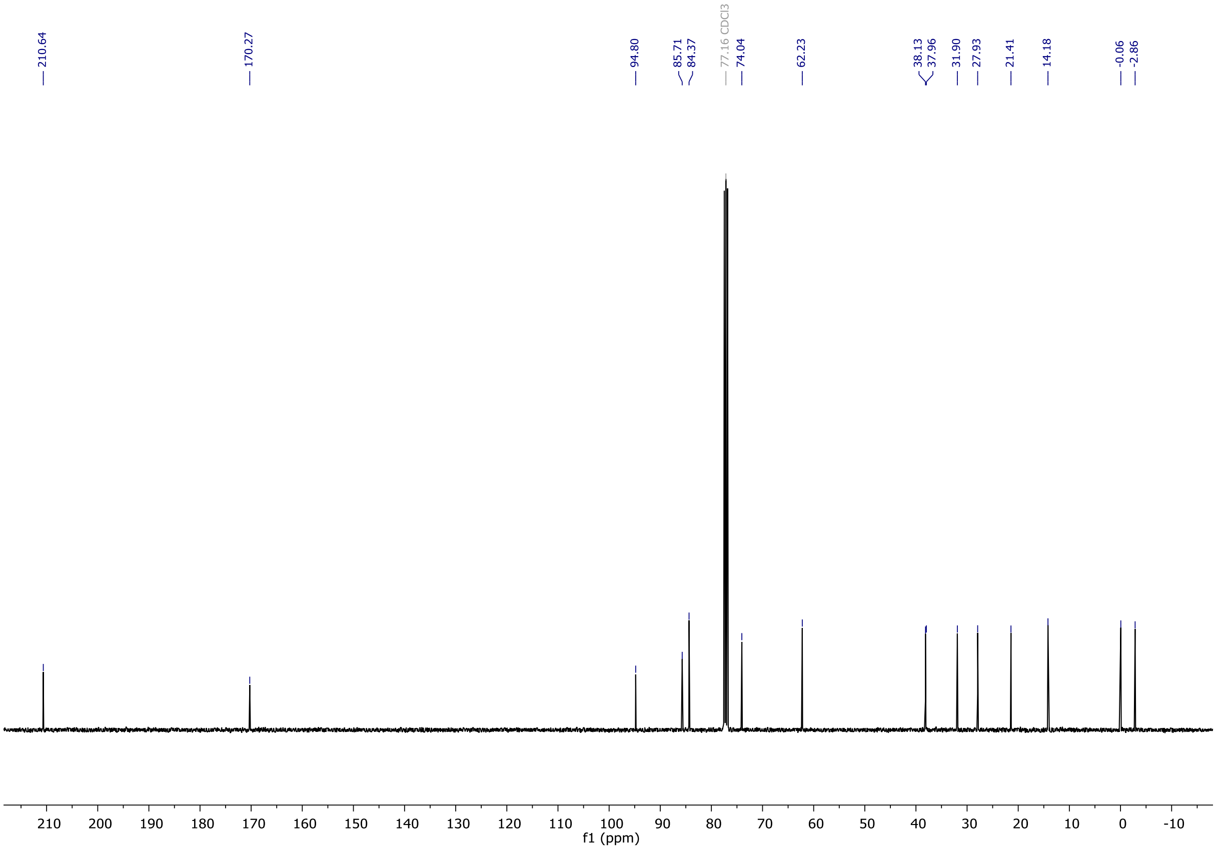

^{1}H NMR (400 MHz, CDCl$_3$, 25 °C) of lactone **22**:

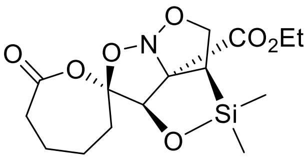

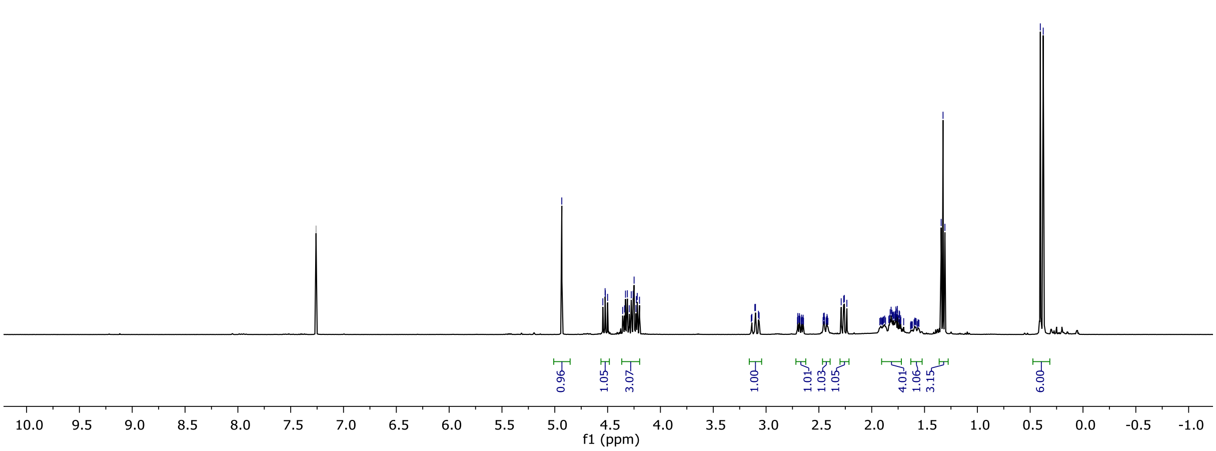

^{13}C NMR (101 MHz, CDCl$_3$, 25 °C) of lactone **22**:

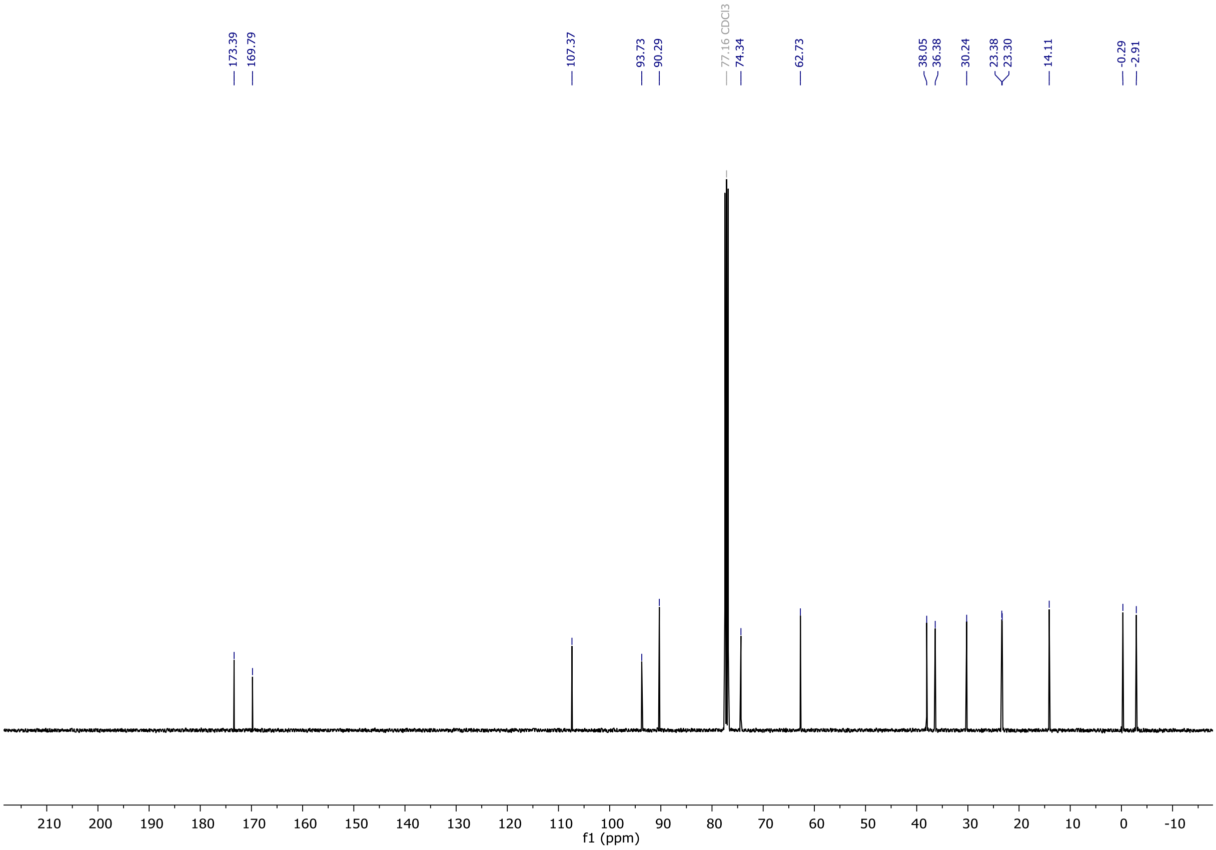

^{1}H NMR (400 MHz, $CDCl_3$, 25 °C) of ester **S14**:

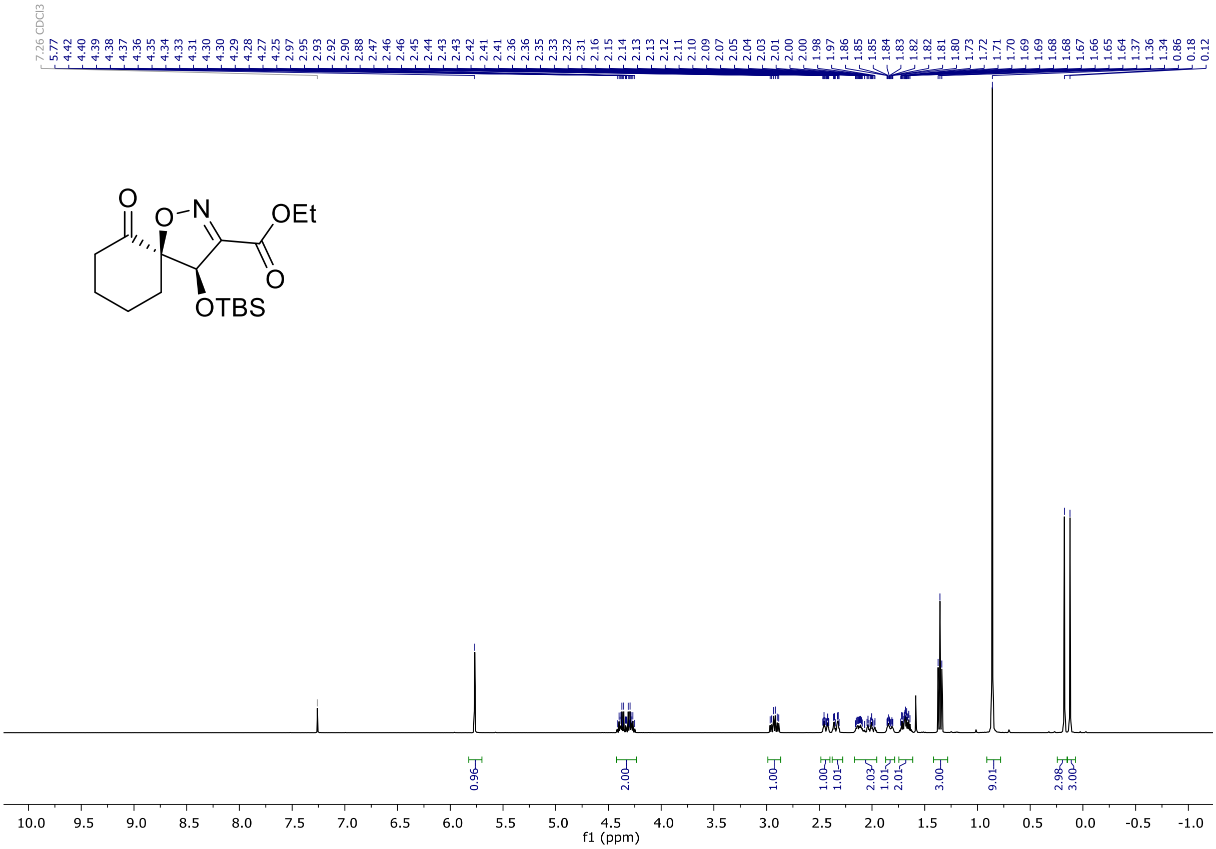

^{13}C NMR (101 MHz, $CDCl_3$, 25 °C) of ester **S14**:

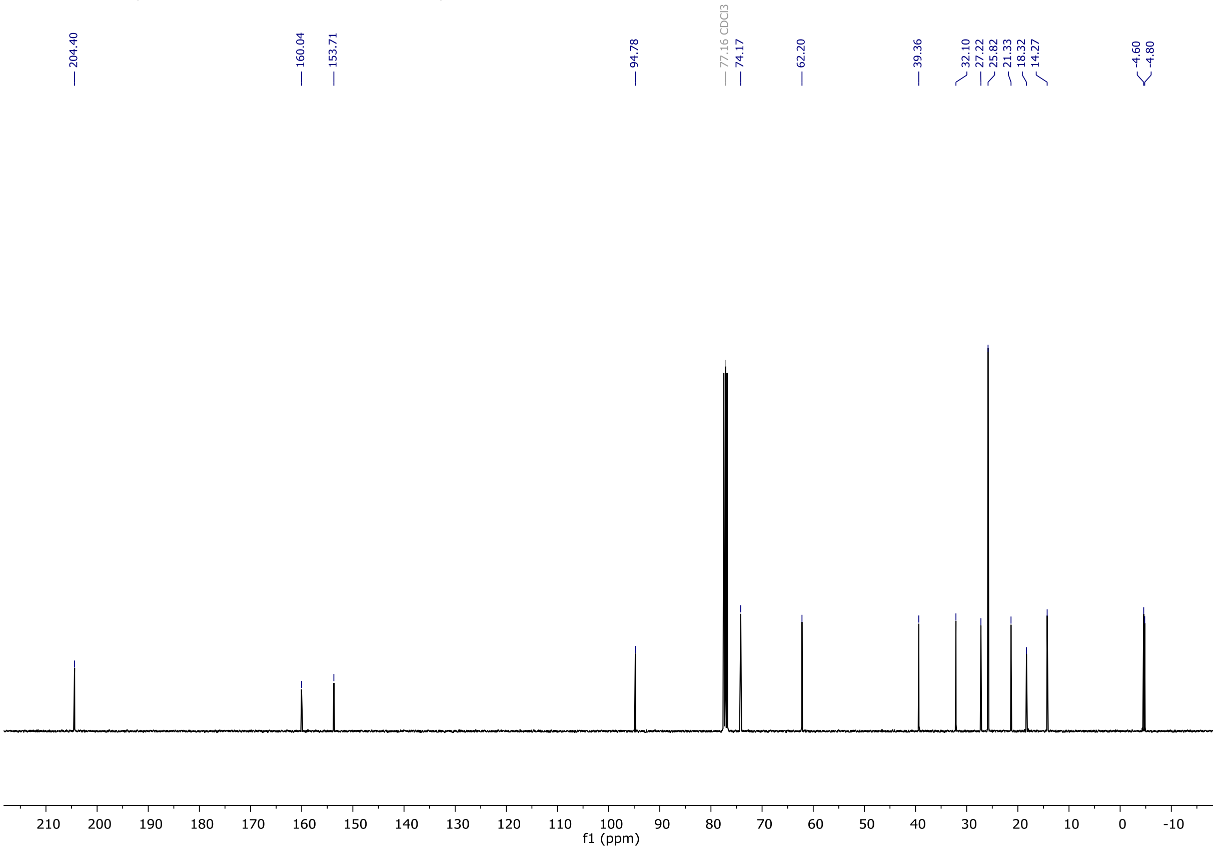

^{1}H NMR (400 MHz, $CDCl_3$, 25 °C) of lactone **15**:

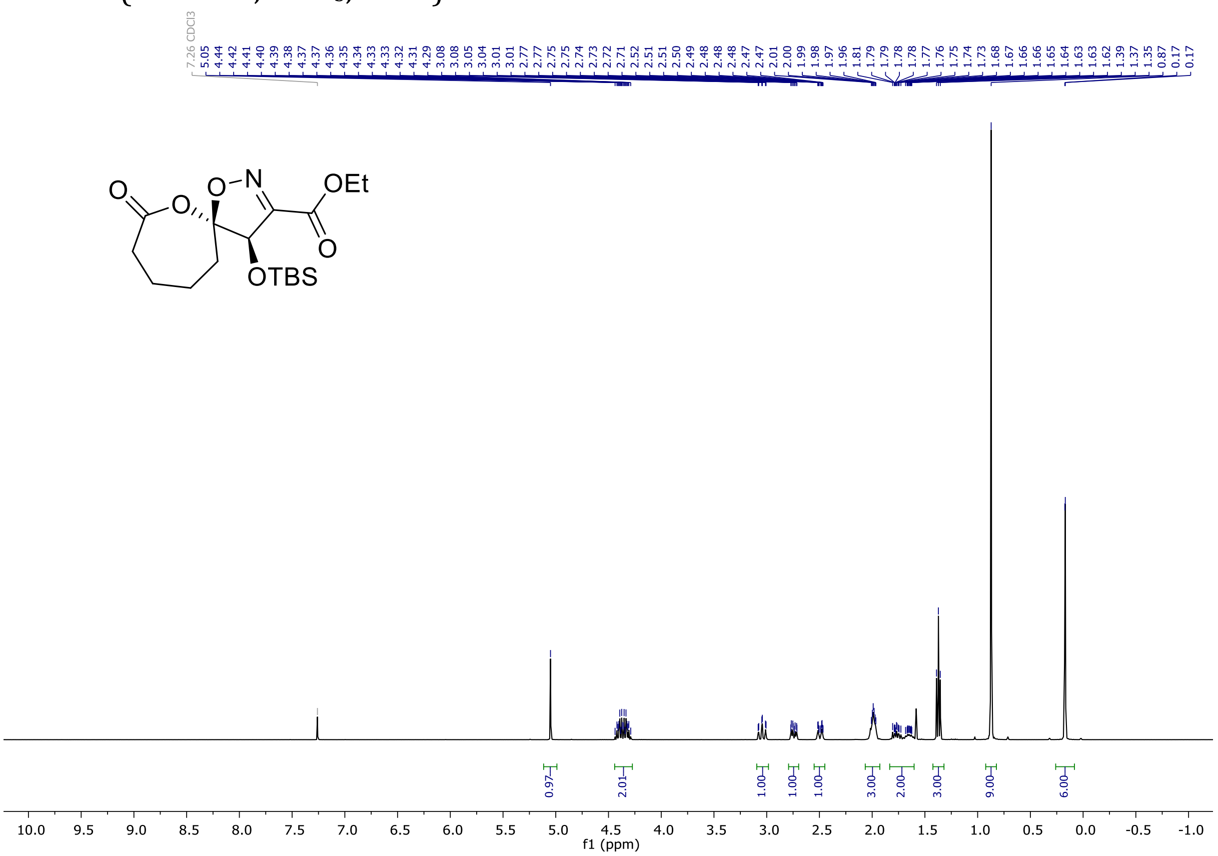

^{13}C NMR (101 MHz, $CDCl_3$, 25 °C) of lactone **15**:

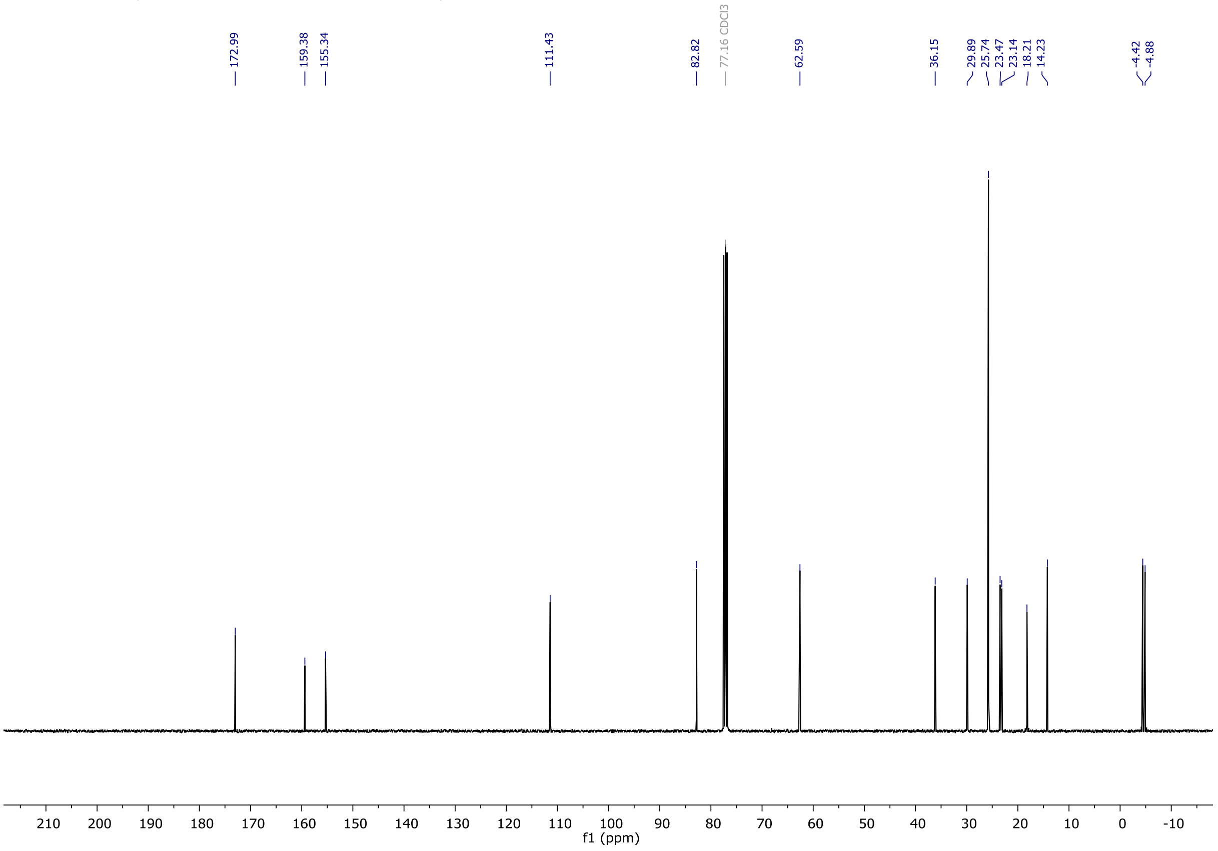

^{1}H NMR (400 MHz, $CDCl_3$, 25 °C) of ketene acetal phosphate **24**:

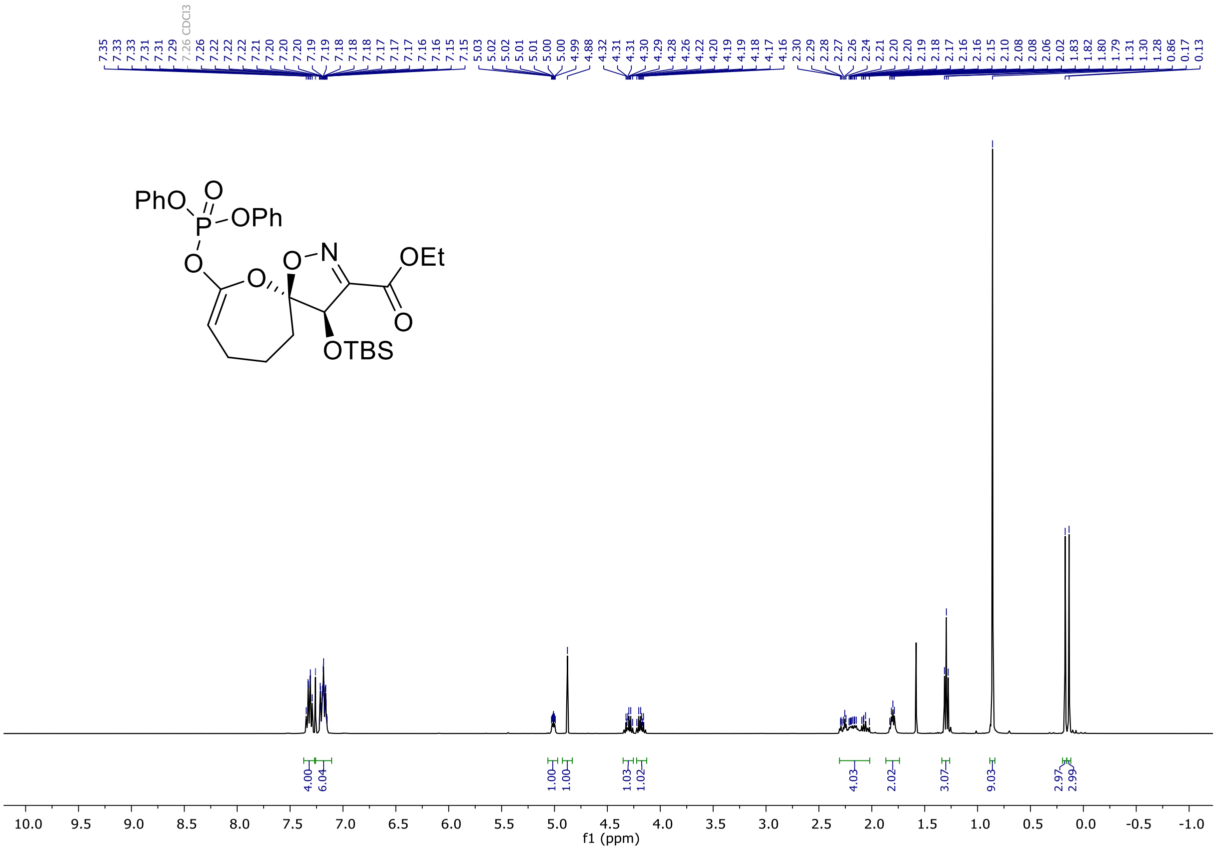

^{13}C NMR (101 MHz, $CDCl_3$, 25 °C) of ketene acetal phosphate **24**:

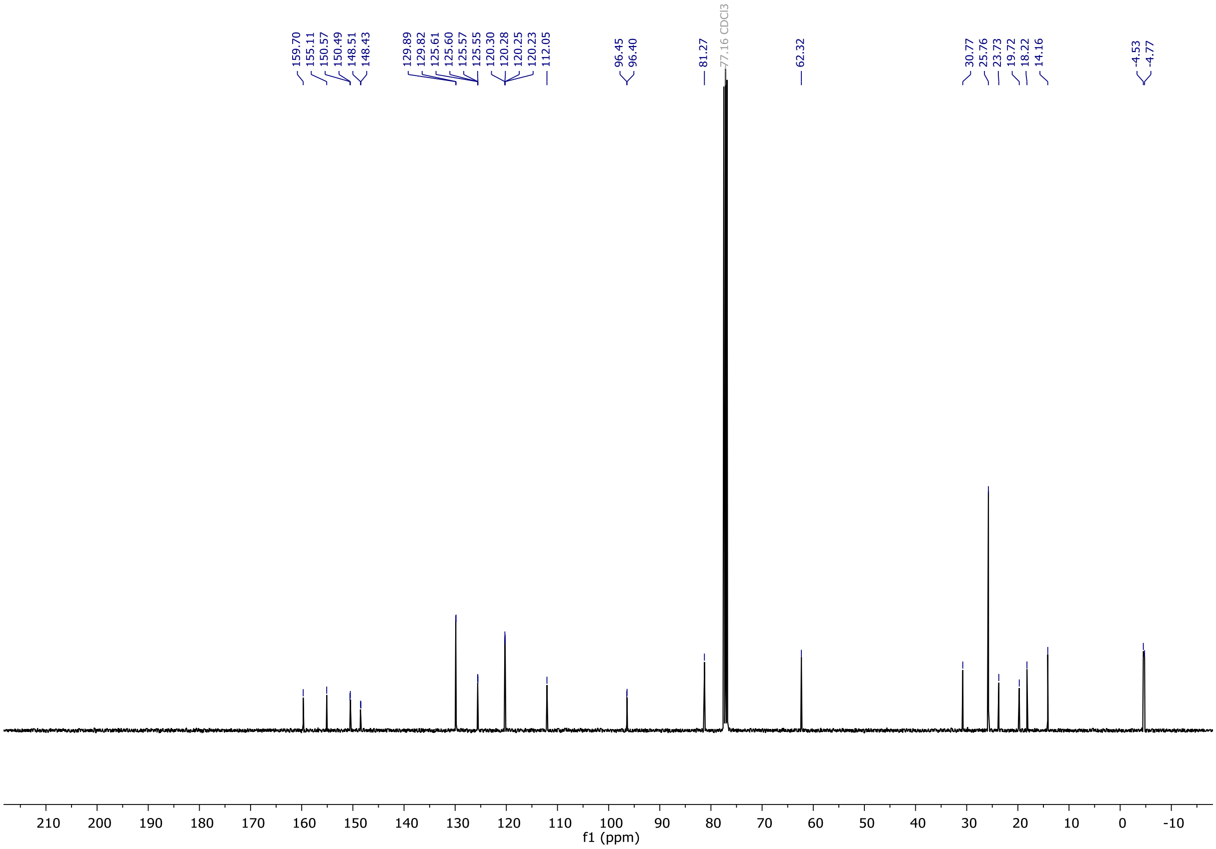

^{1}H NMR (400 MHz, CDCl$_3$, 25 °C) of enol ether **25**:

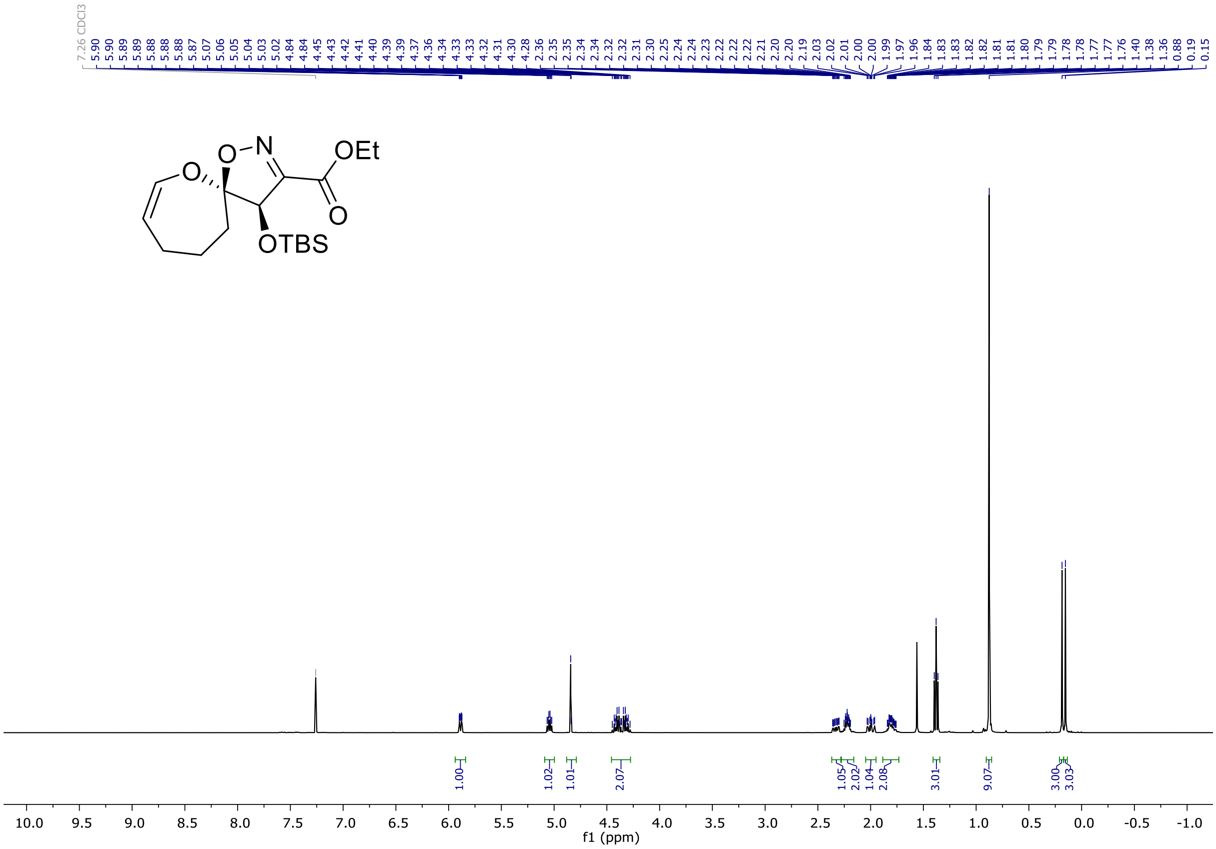

^{13}C NMR (101 MHz, CDCl$_3$, 25 °C) of enol ether **25**:

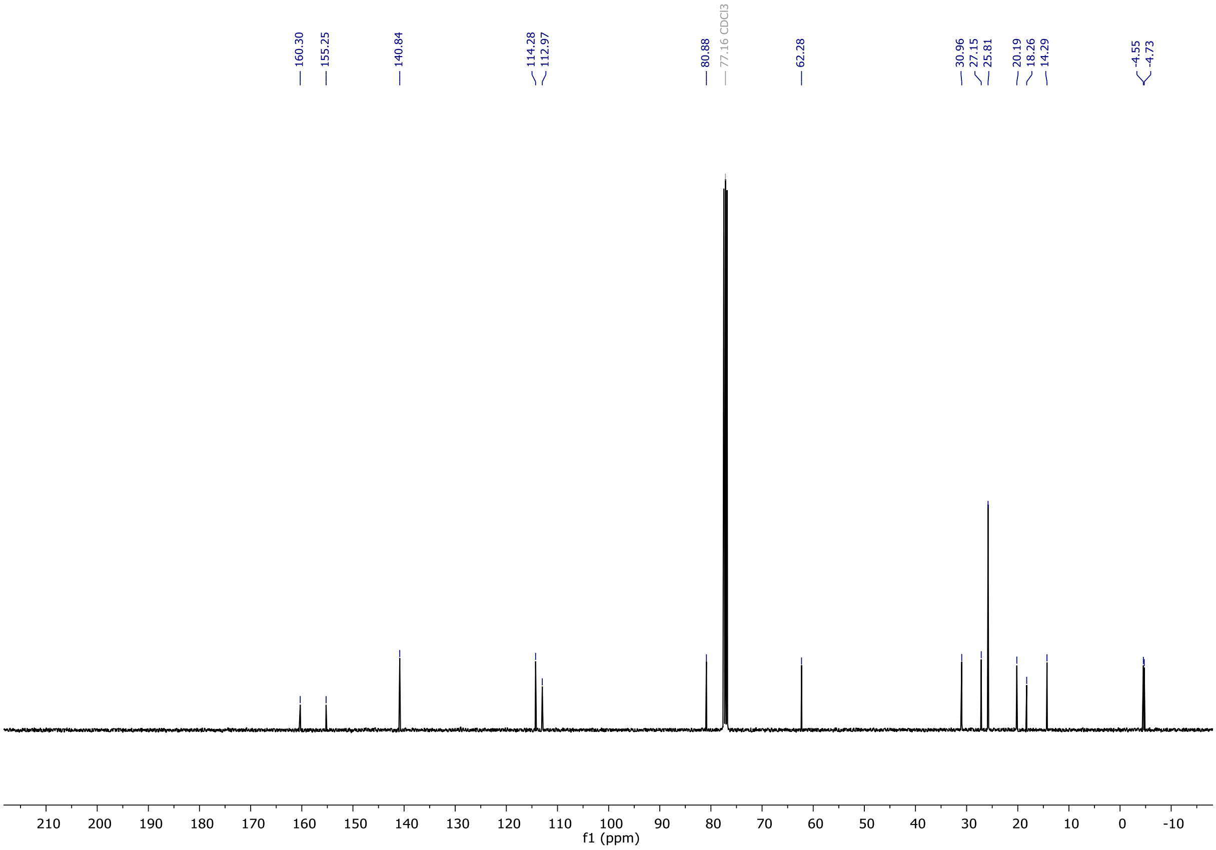

^{1}H NMR (400 MHz, $CDCl_3$, 25 °C) of vinylogous lactone **26**:

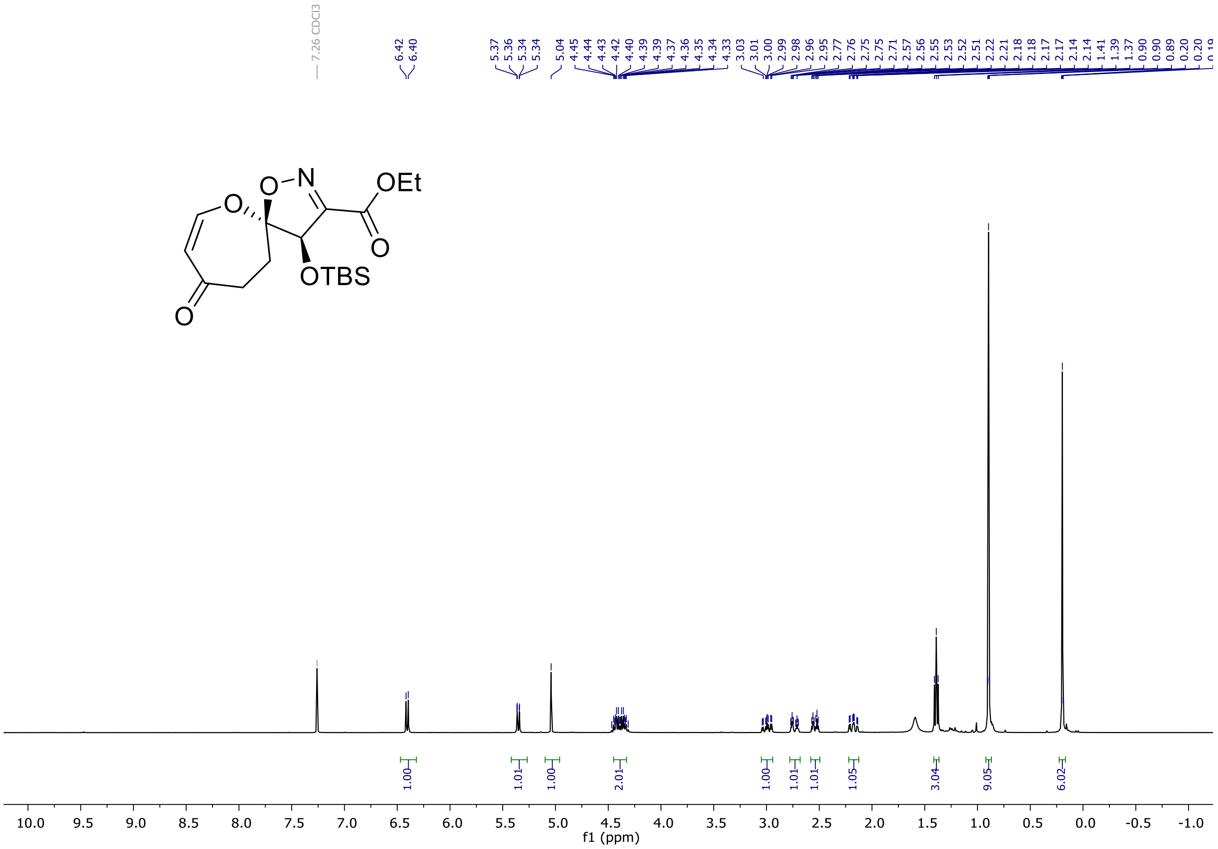

^{13}C NMR (101 MHz, $CDCl_3$, 25 °C) of vinylogous lactone **26**:

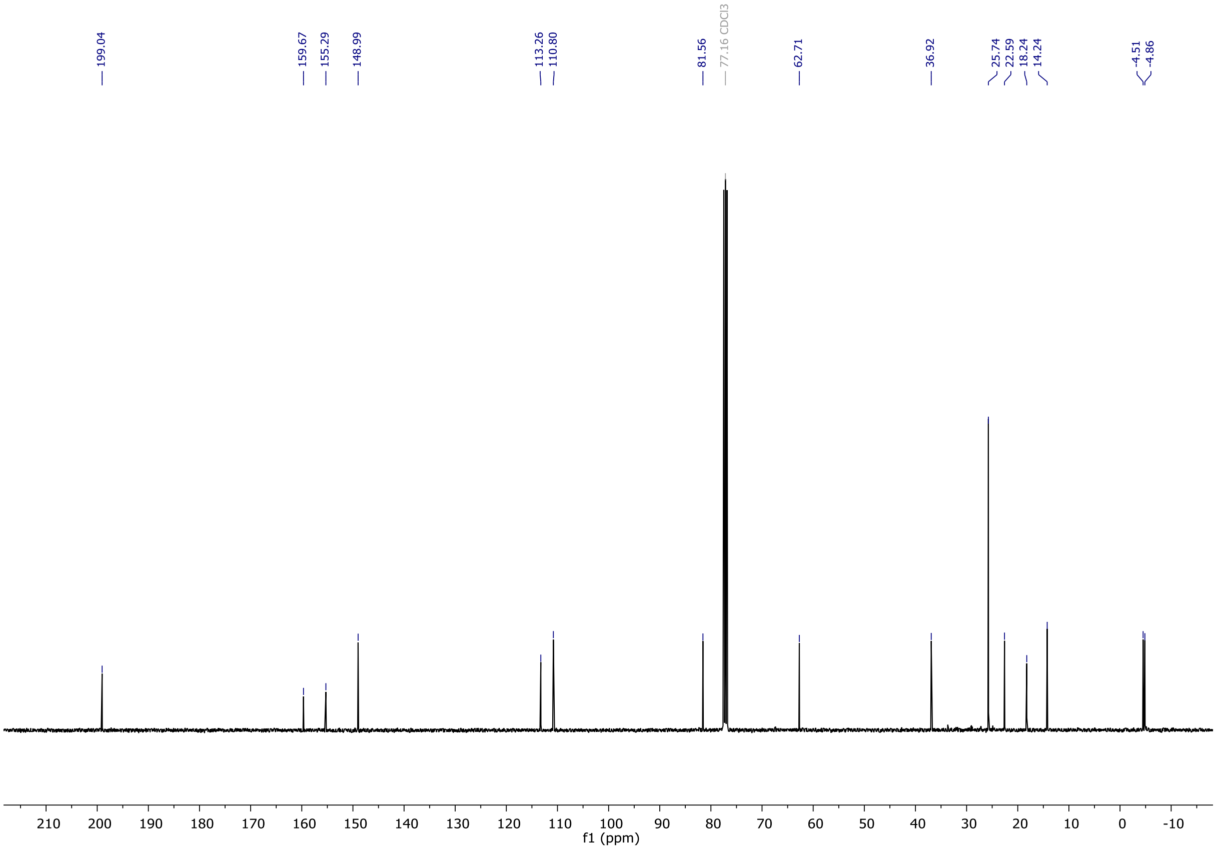

^{1}H NMR (400 MHz, $CDCl_3$, 25 °C) of lactone **27**:

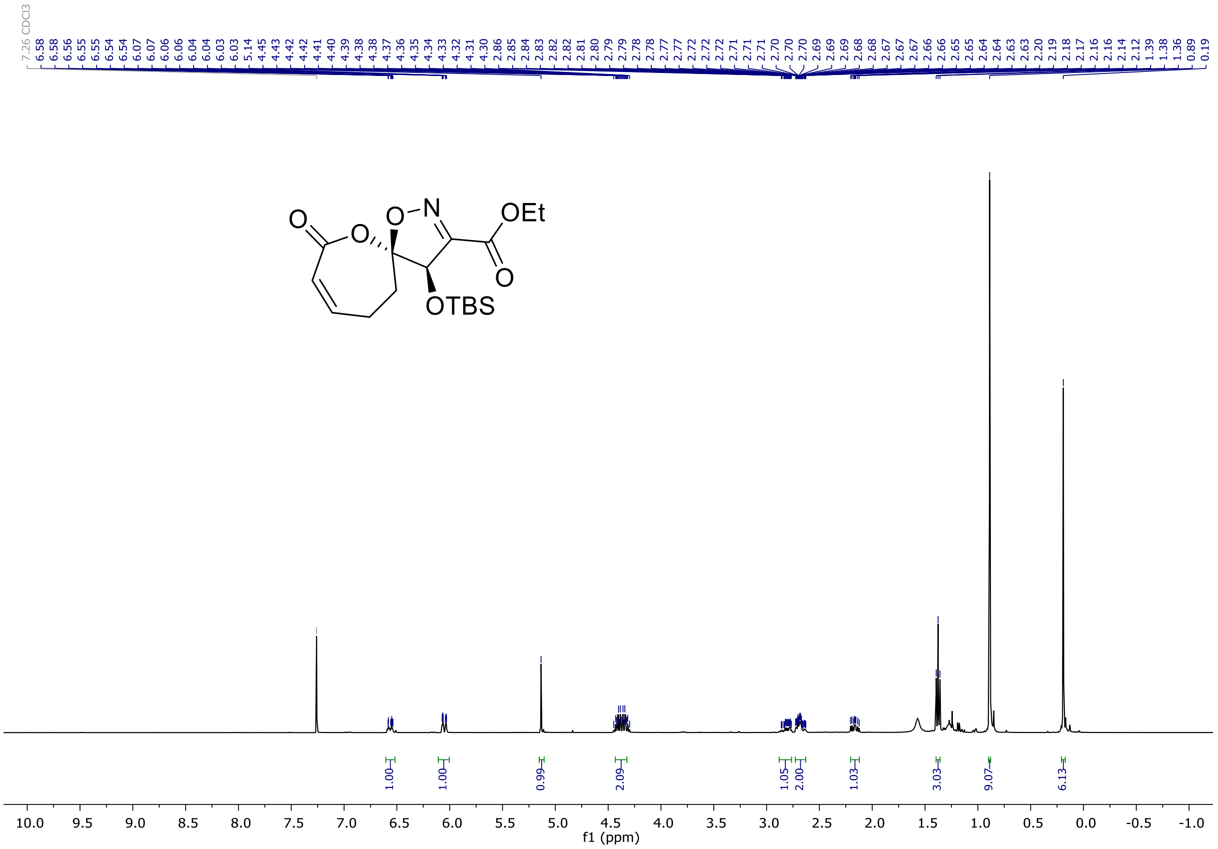

^{13}C NMR (101 MHz, $CDCl_3$, 25 °C) of lactone **27**:

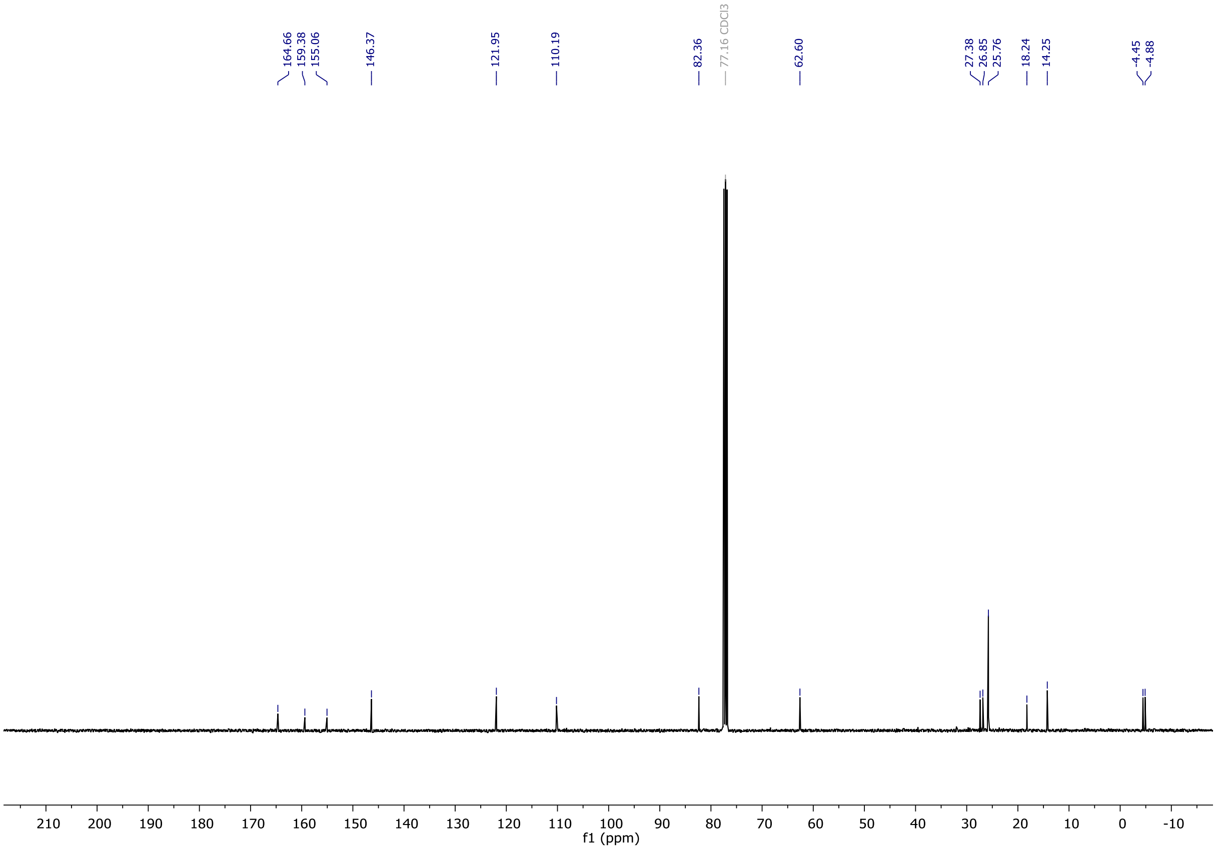

^{1}H NMR (400 MHz, $CDCl_3$, 25 °C) of vinylogous lactone **S16**:

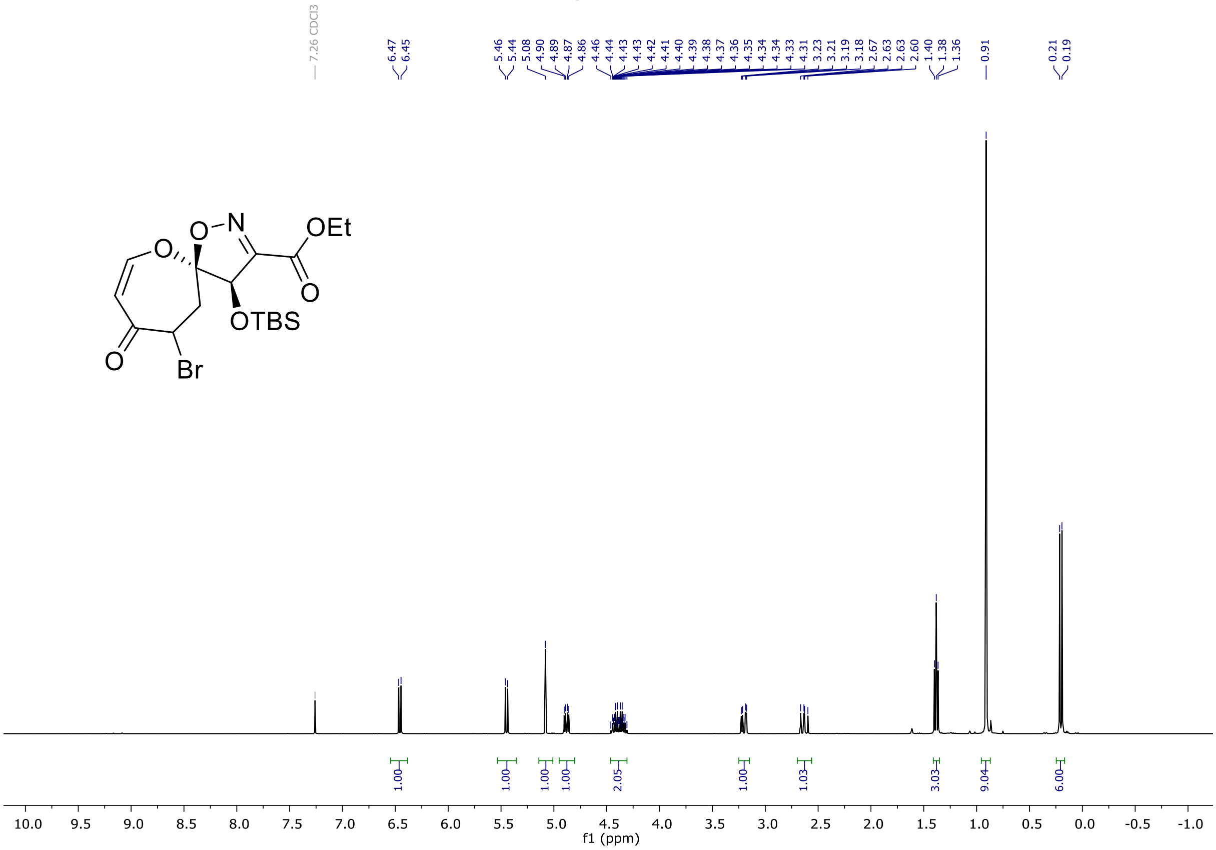

^{13}C NMR (101 MHz, $CDCl_3$, 25 °C) of vinylogous lactone **S16**:

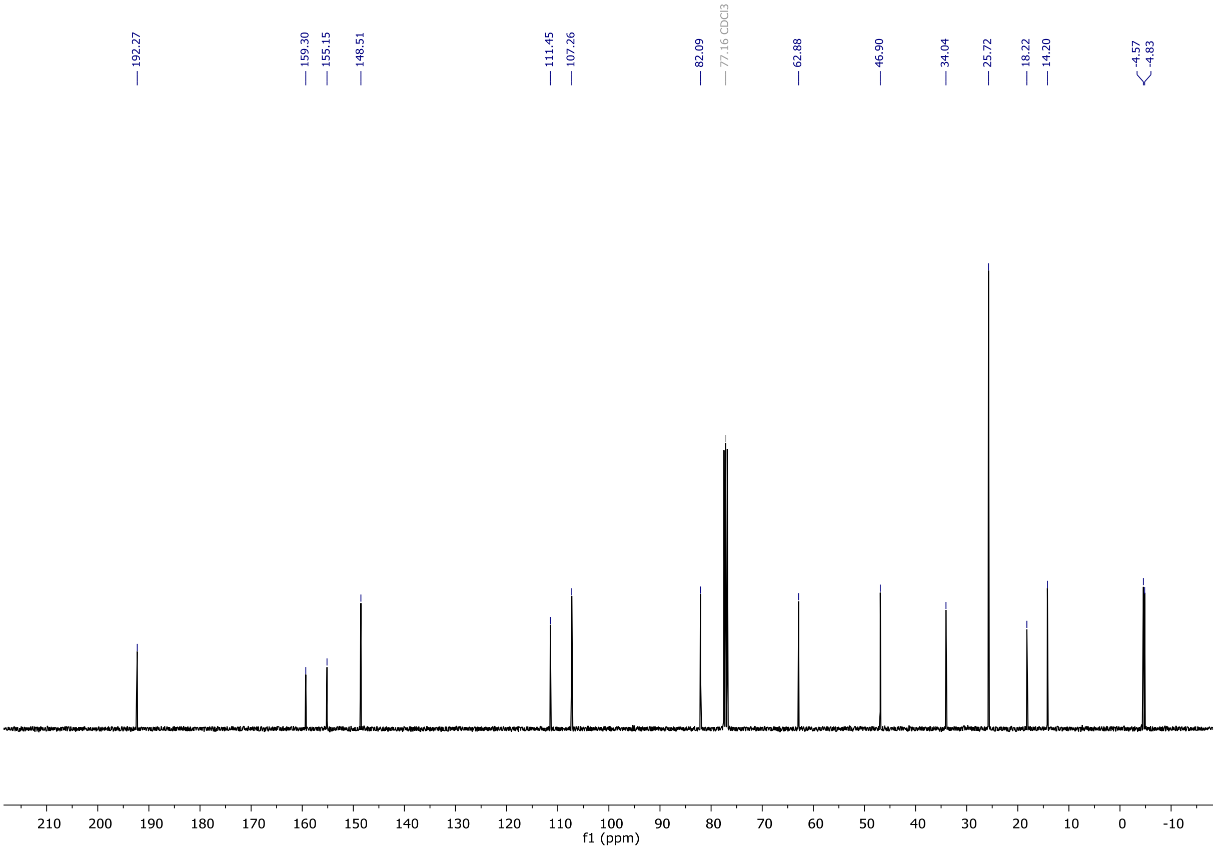

^{1}H NMR (400 MHz, $CDCl_3$, 25 °C) of vinylogous lactone **28**:

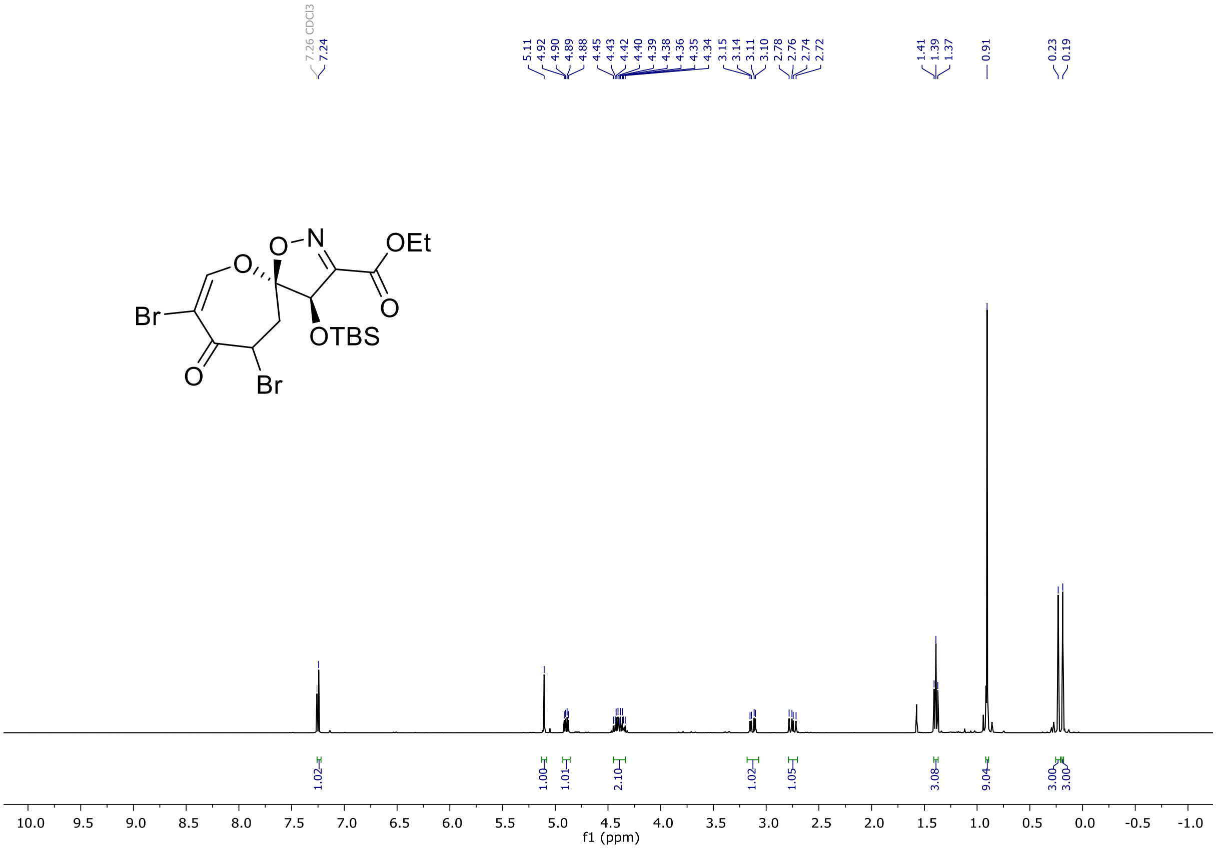

^{13}C NMR (101 MHz, $CDCl_3$, 25 °C) of vinylogous lactone **28**:

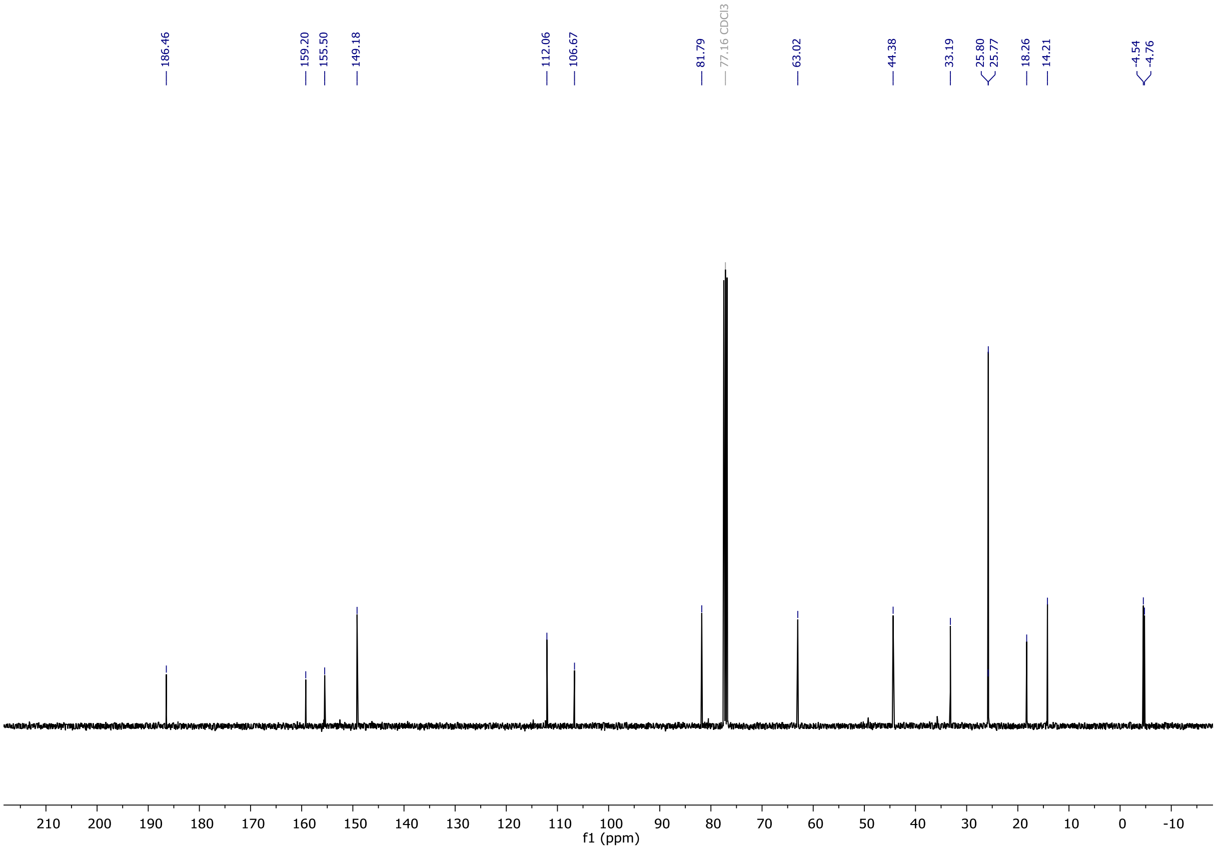

^{1}H NMR (400 MHz, $CDCl_3$, 25 °C) of ester **14**:

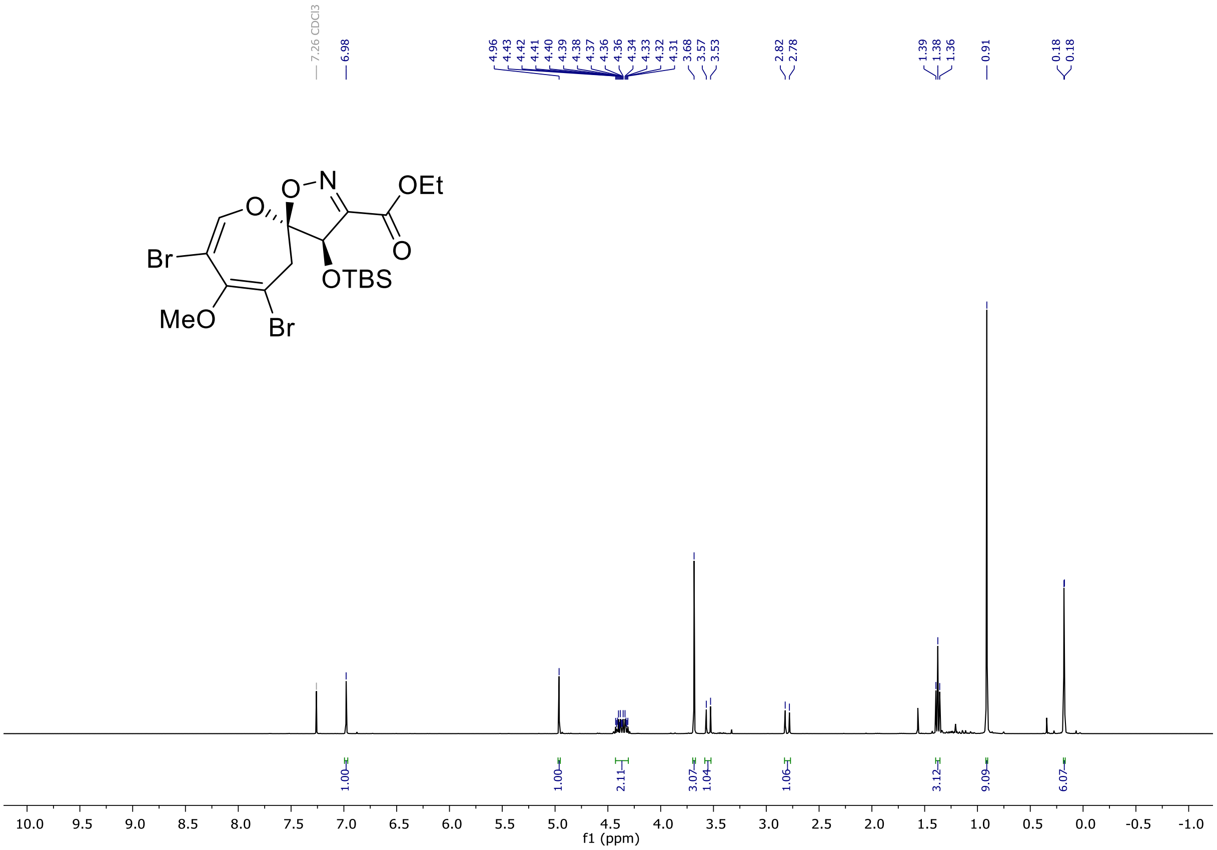

^{13}C NMR (101 MHz, $CDCl_3$, 25 °C) of ester **14**:

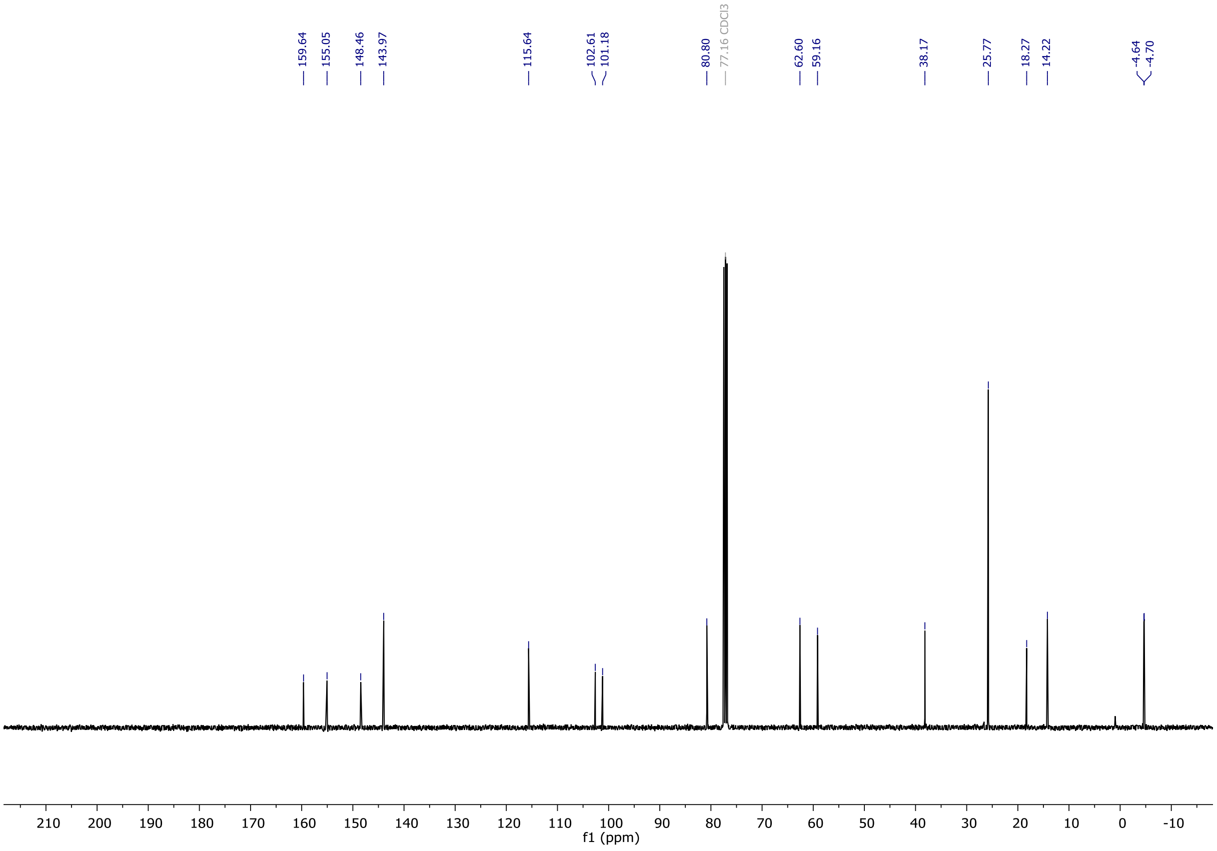

^{1}H NMR (400 MHz, CDCl$_3$, 25 °C) of alcohol **S17**:

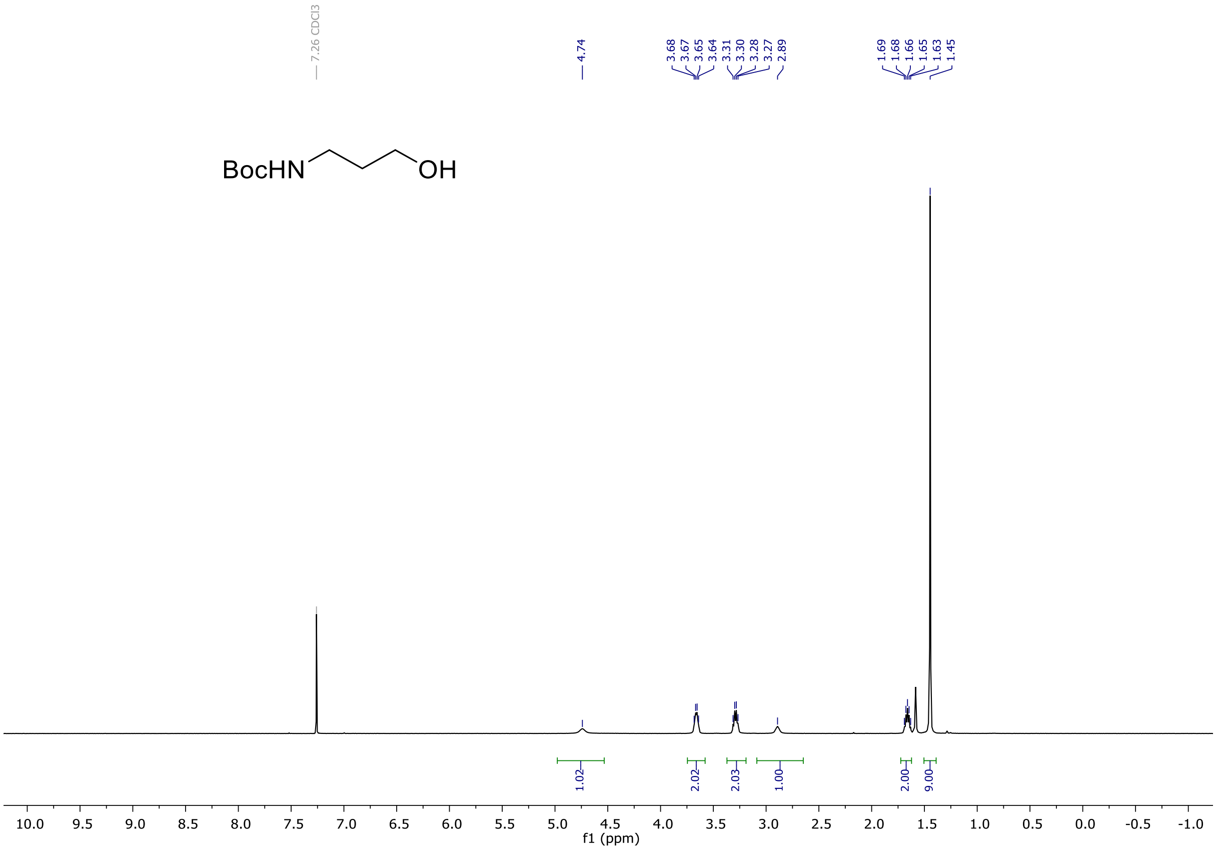

^{1}H NMR (400 MHz, CDCl$_3$, 25 °C) of bromide **S18**:

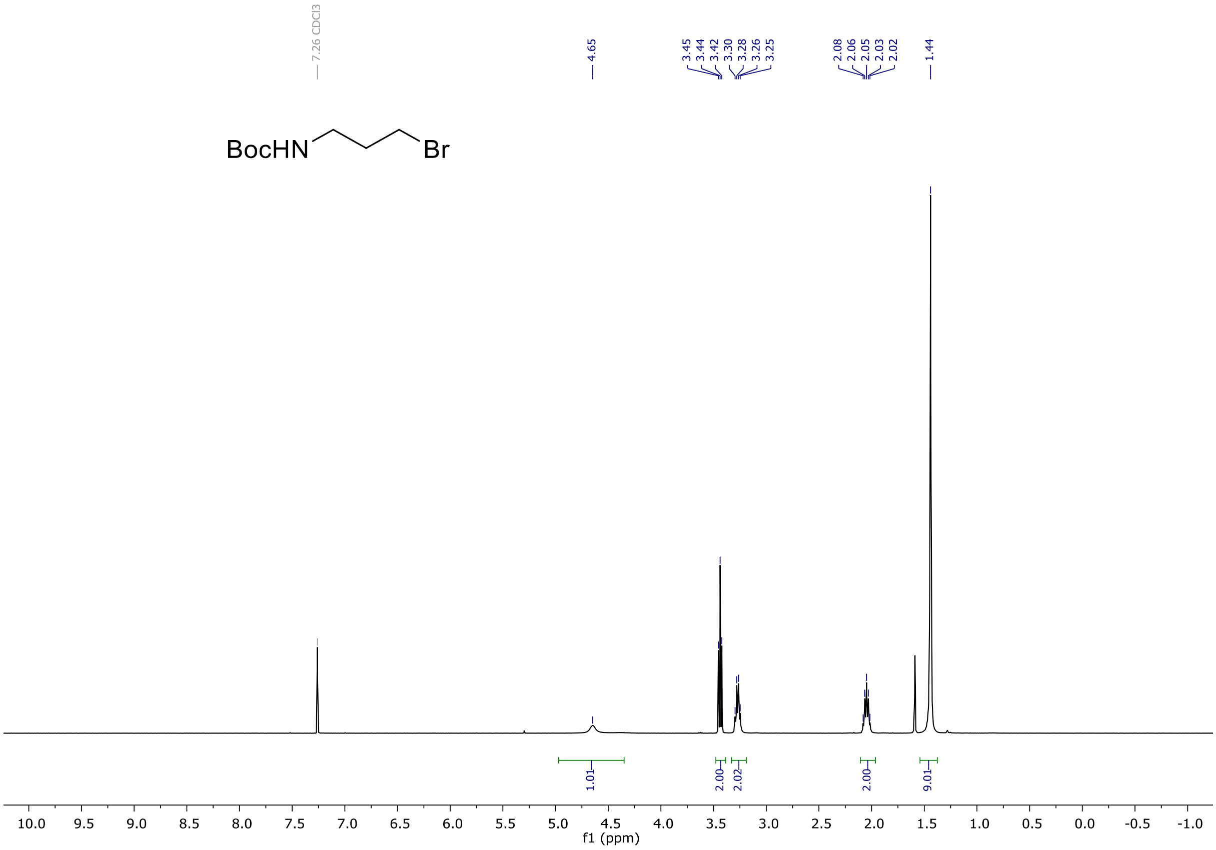

^{1}H NMR (400 MHz, DMSO-d_6, 25 °C) of phthalimide **S19**:

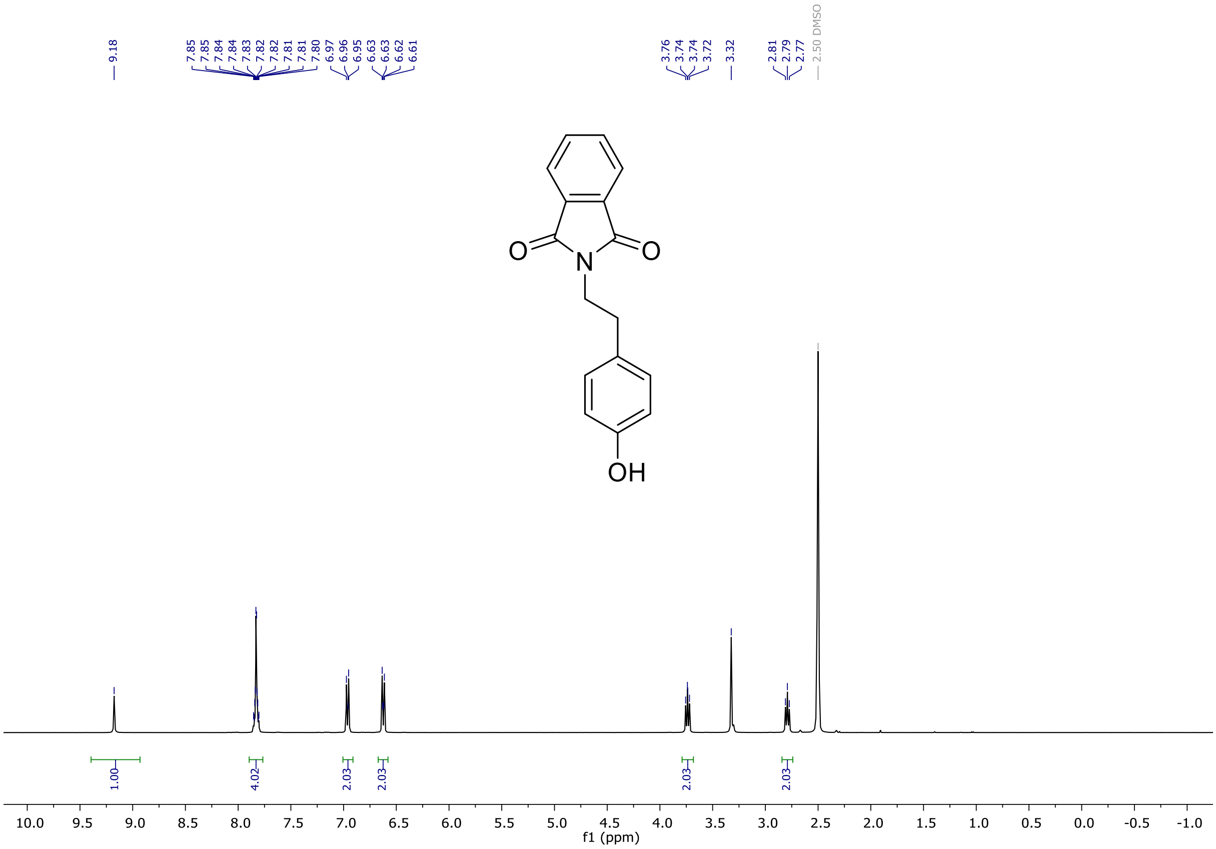

^{1}H NMR (400 MHz, $CDCl_3$, 25 °C) of phthalimide **S20**:

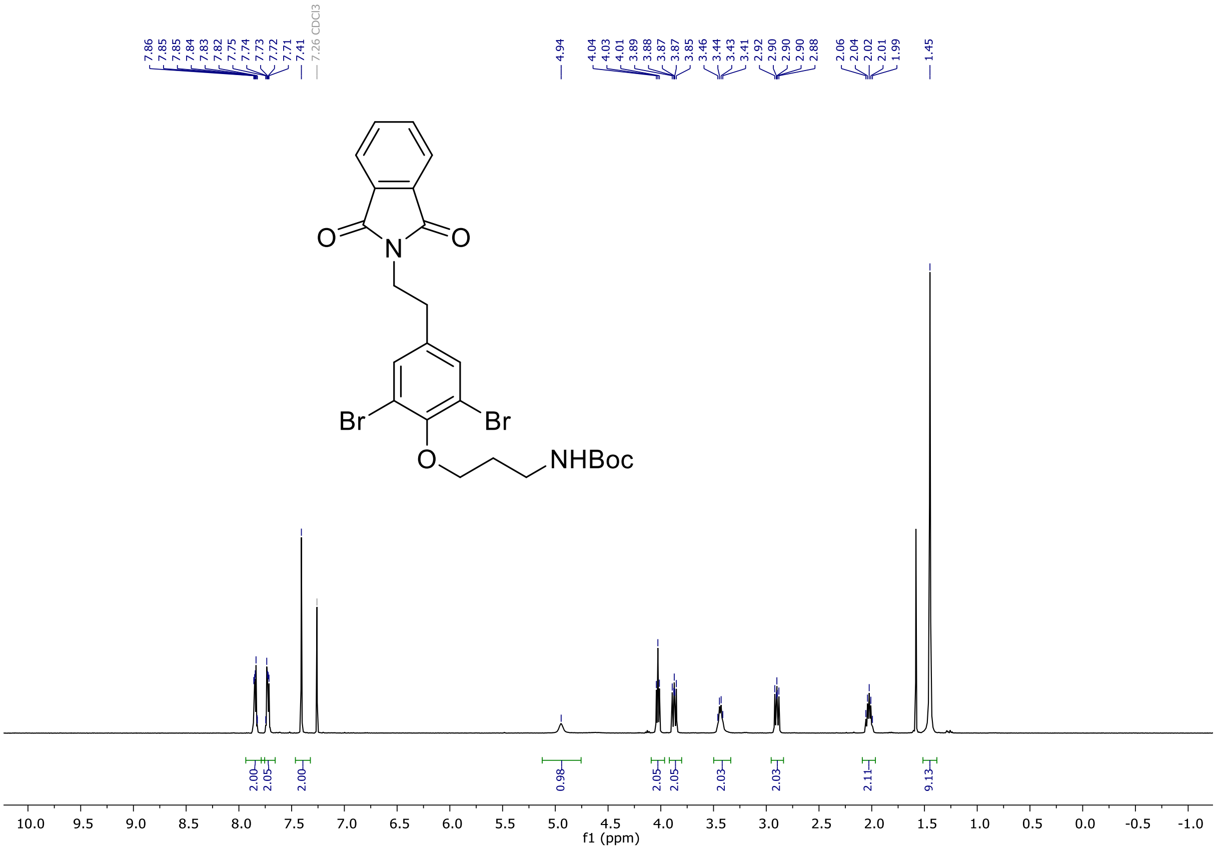

^{13}C NMR (101 MHz, $CDCl_3$, 25 °C) of phthalimide **S20**:

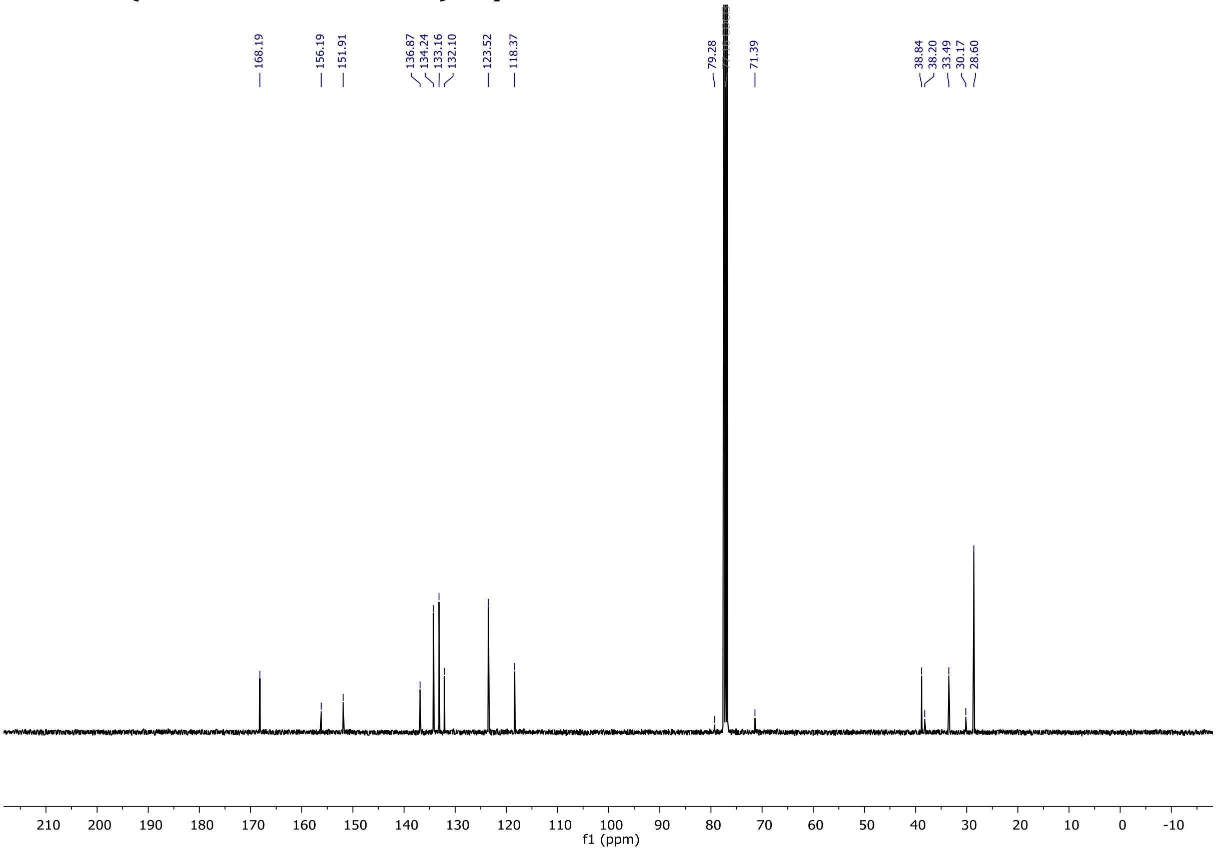

^{1}H NMR (400 MHz, CD_3OD, 25 °C) of ammonium salt **29**:

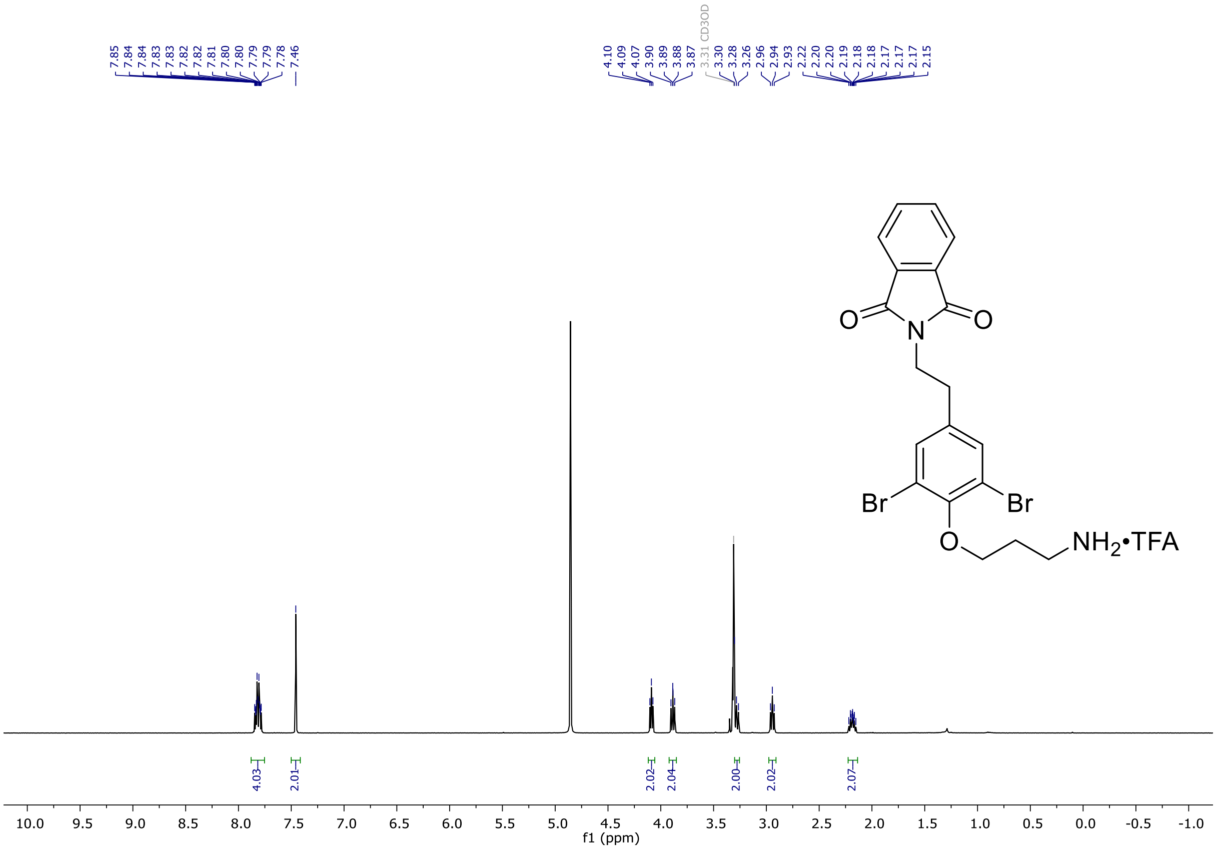

^{13}C NMR (101 MHz, CD_3OD, 25 °C) of ammonium salt **29**:

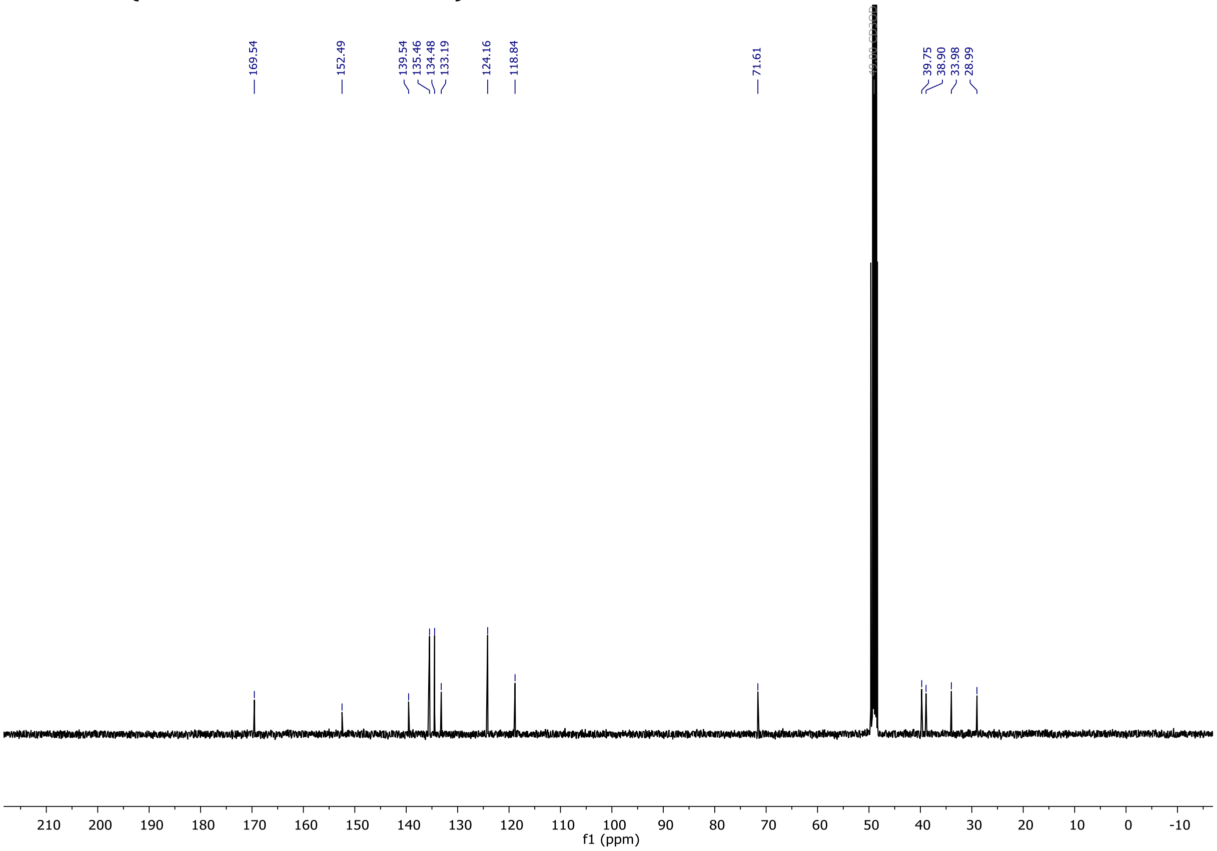

^{1}H NMR (400 MHz, $CDCl_3$, 25 °C) of amide **30**:

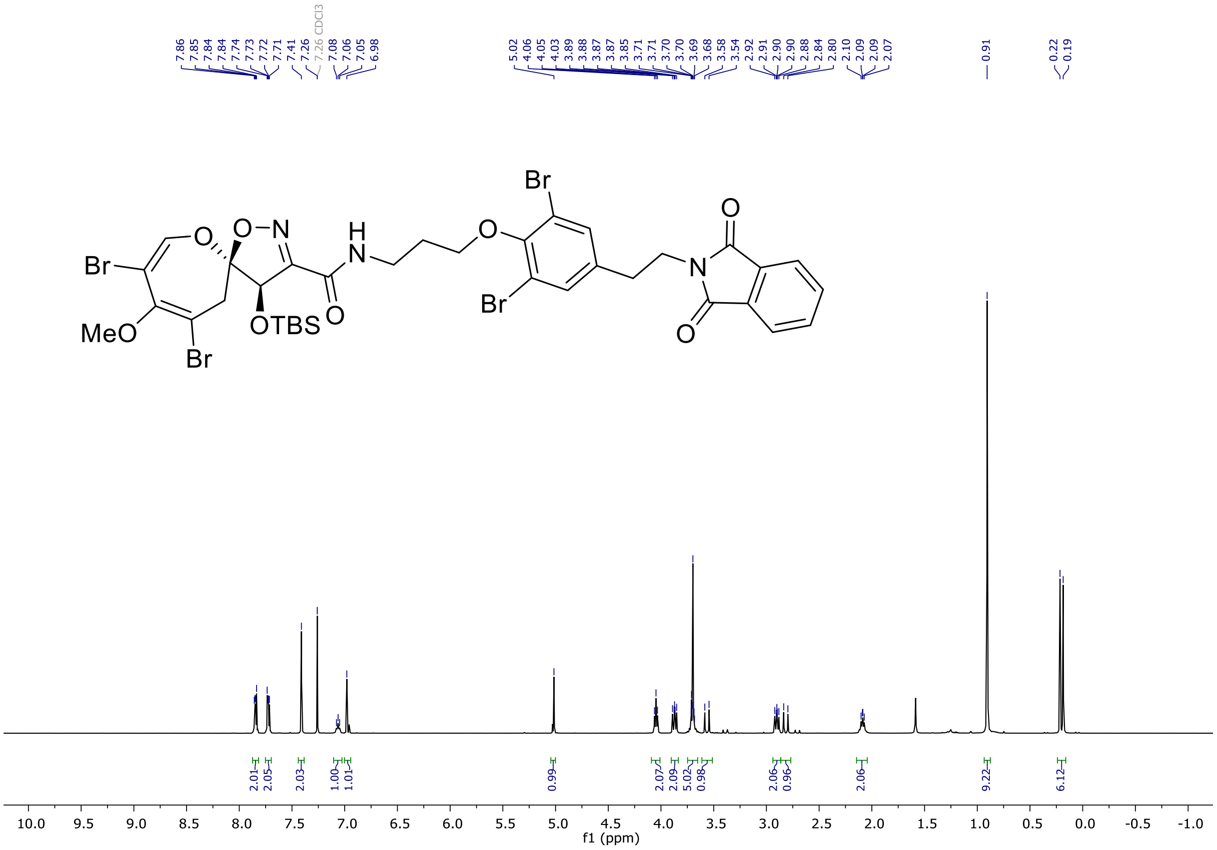

^{13}C NMR (101 MHz, $CDCl_3$, 25 °C) of amide **30**:

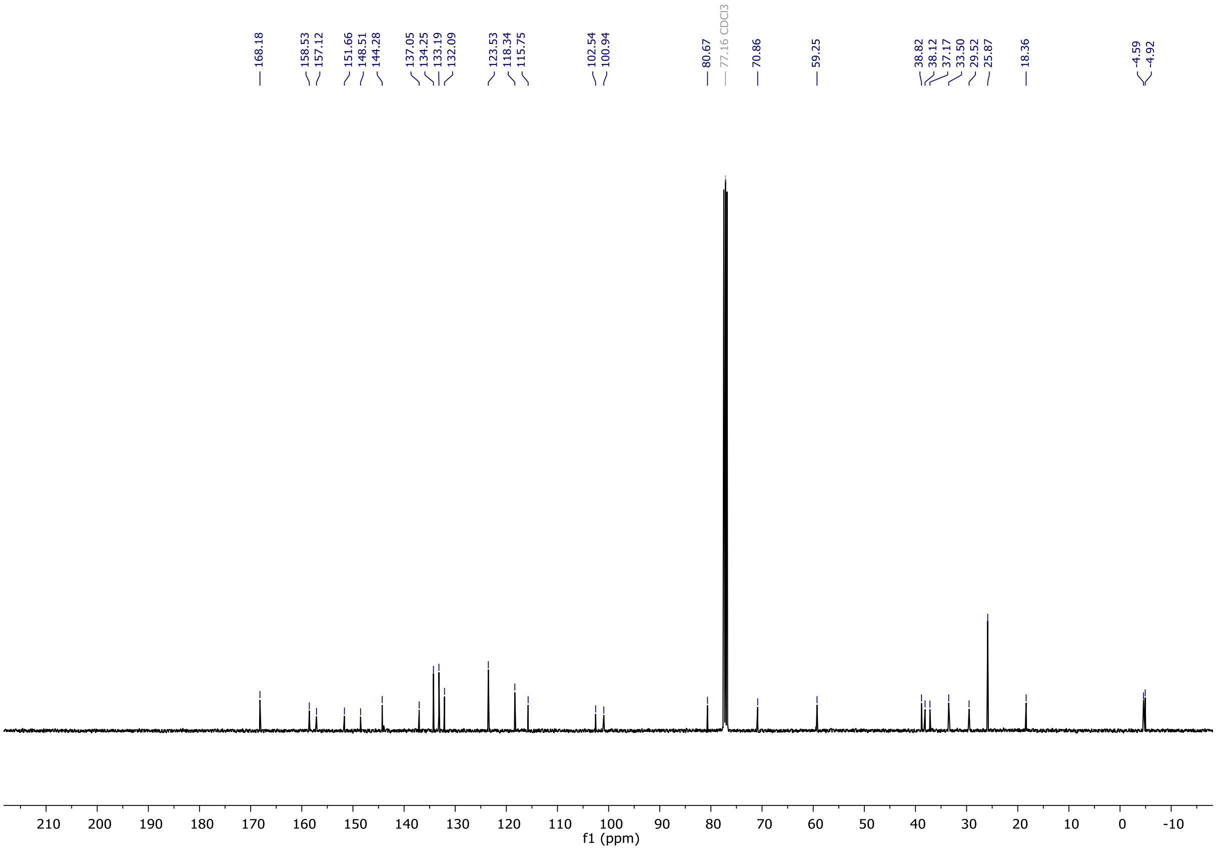

^{1}H NMR (400 MHz, CD_3OD, 25 °C) of psammaplysin A (**1**):

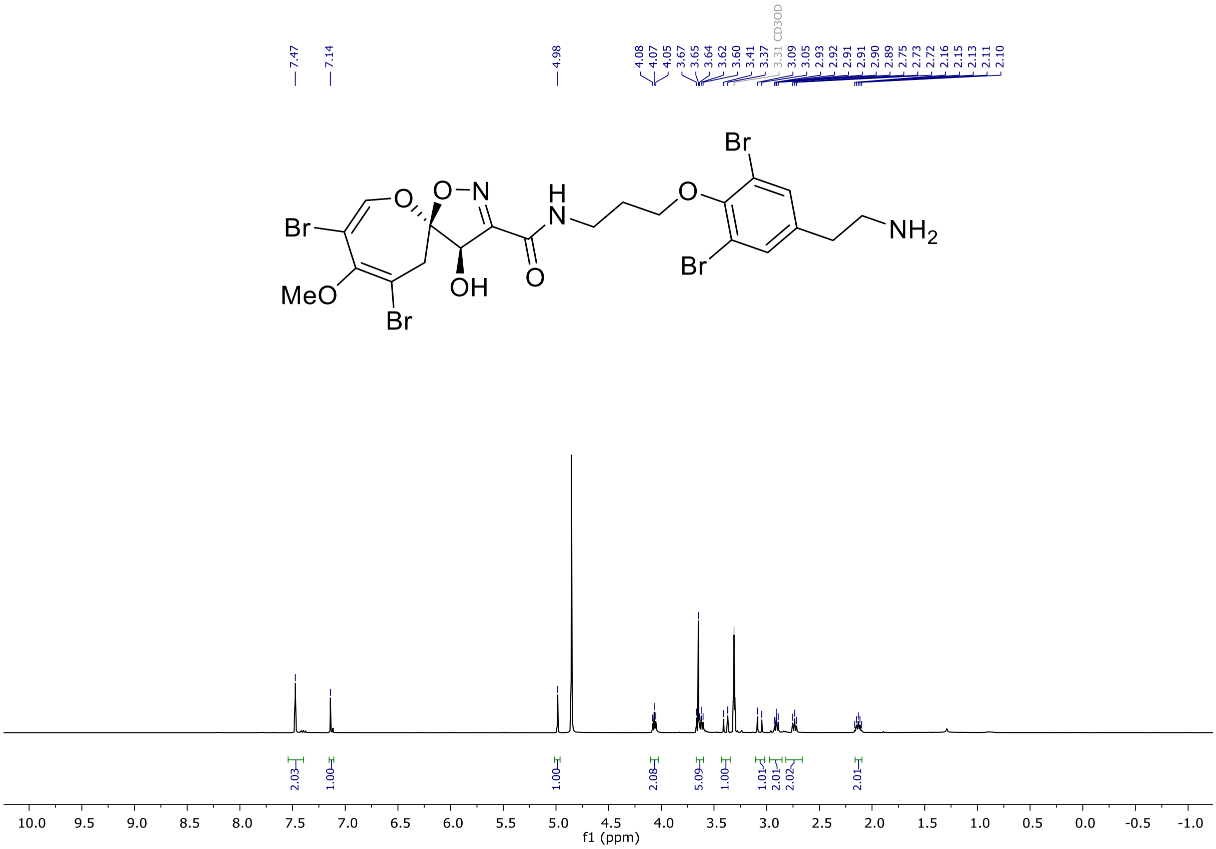

^{1}H NMR (400MHz, CD_2Cl_2, 25 °C) of psammaplysin A (**1**):

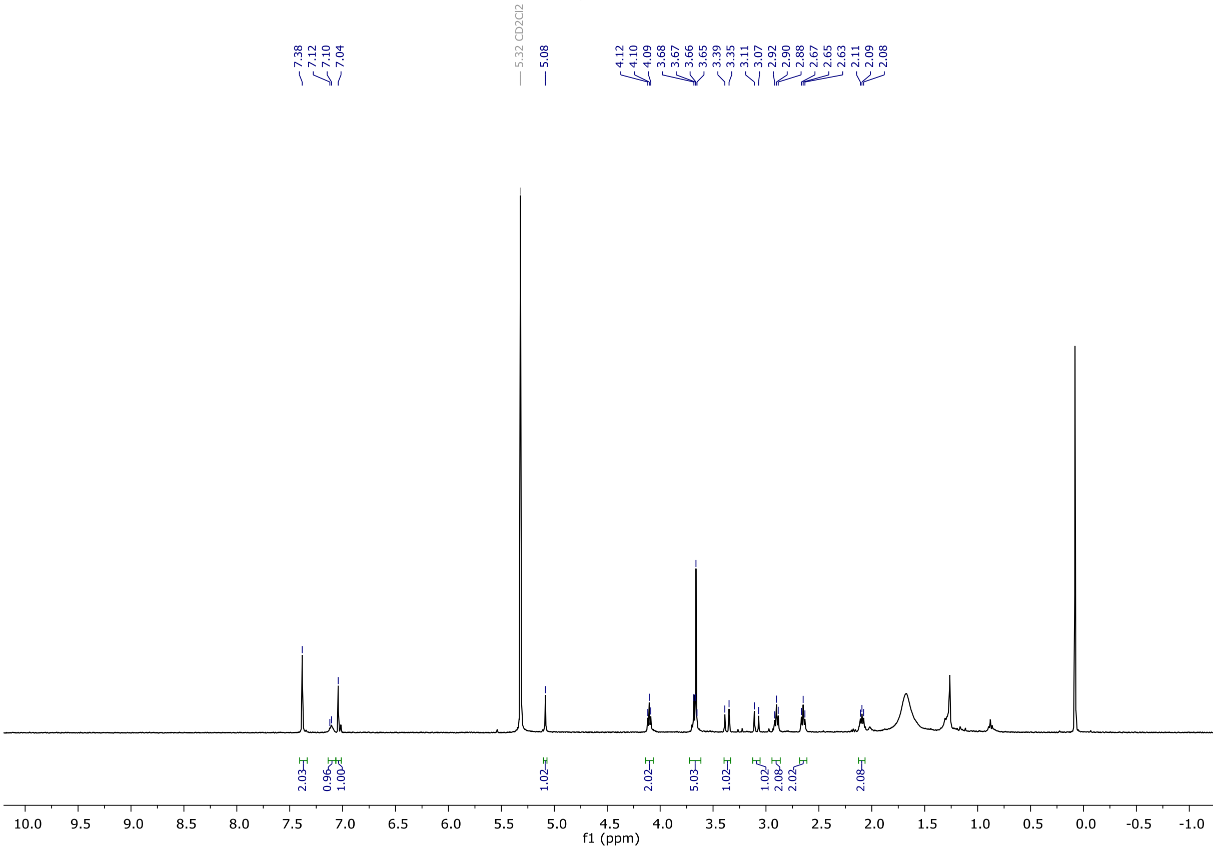

^{13}C NMR (101 MHz, CD_3OD, 25 °C) of psammaplysin A (**1**):

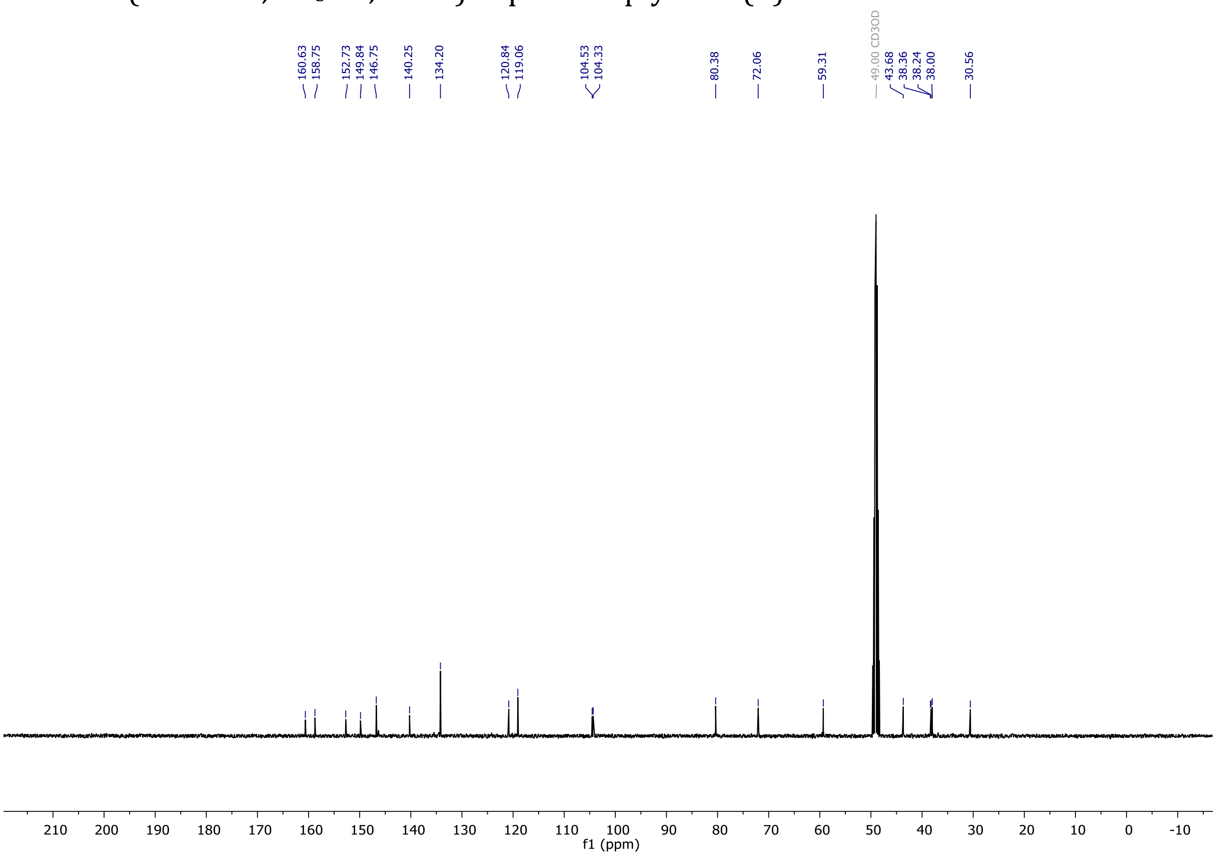

H-H COSY NMR (CD_3OD, 25 °C) of psammaplysin A (**1**):

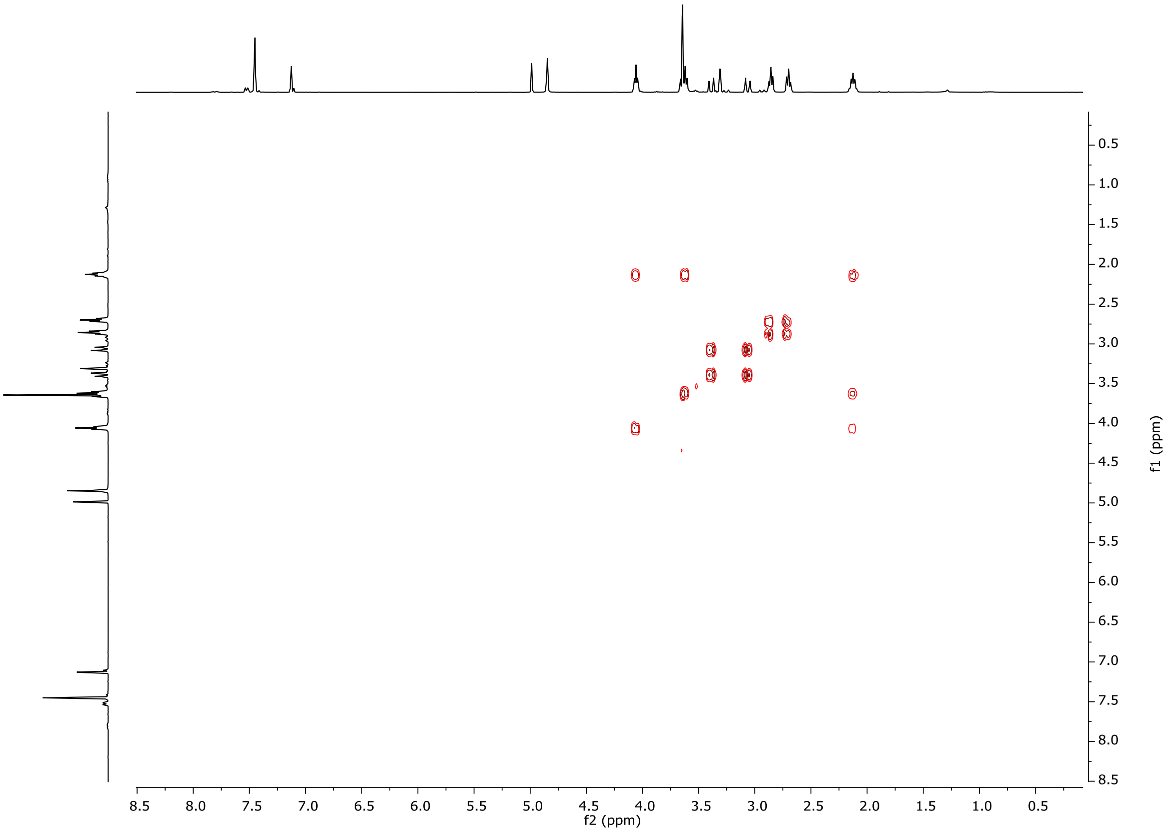

C-H HSQC NMR (CD_3OD, 25 °C) of psammaplysin A (**1**):

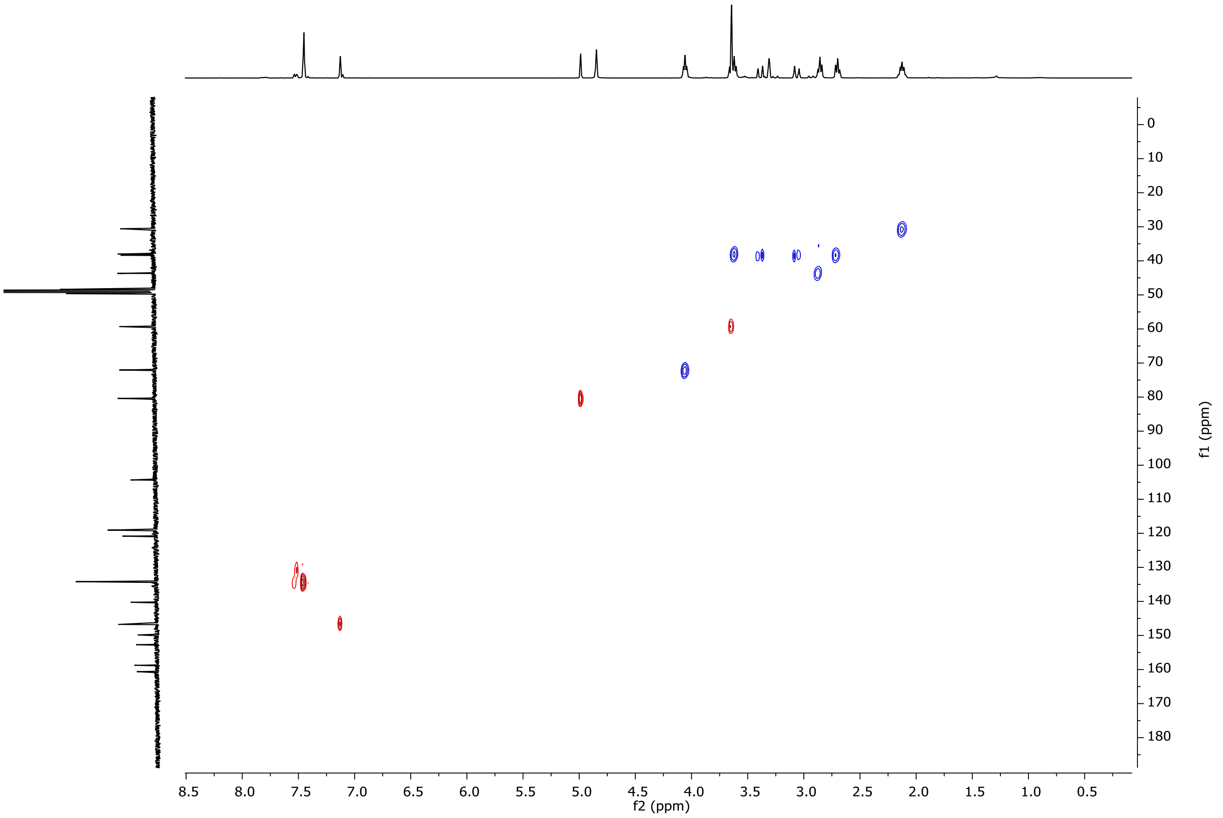

C-H HMBC NMR (CD_3OD, 25 °C) of psammaplysin A (**1**):

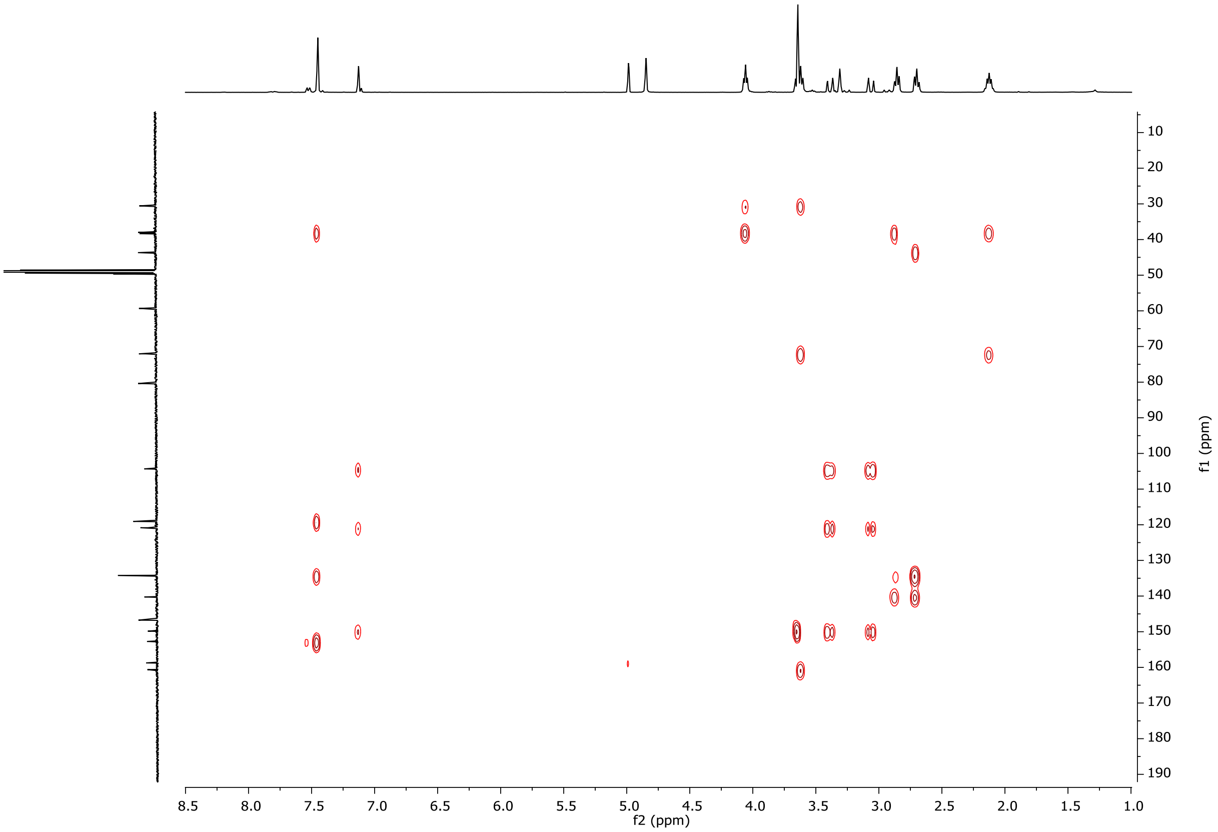

^{1}H NMR (400 MHz, CD_2Cl_2, 25 °C) of acetamide **S21**:

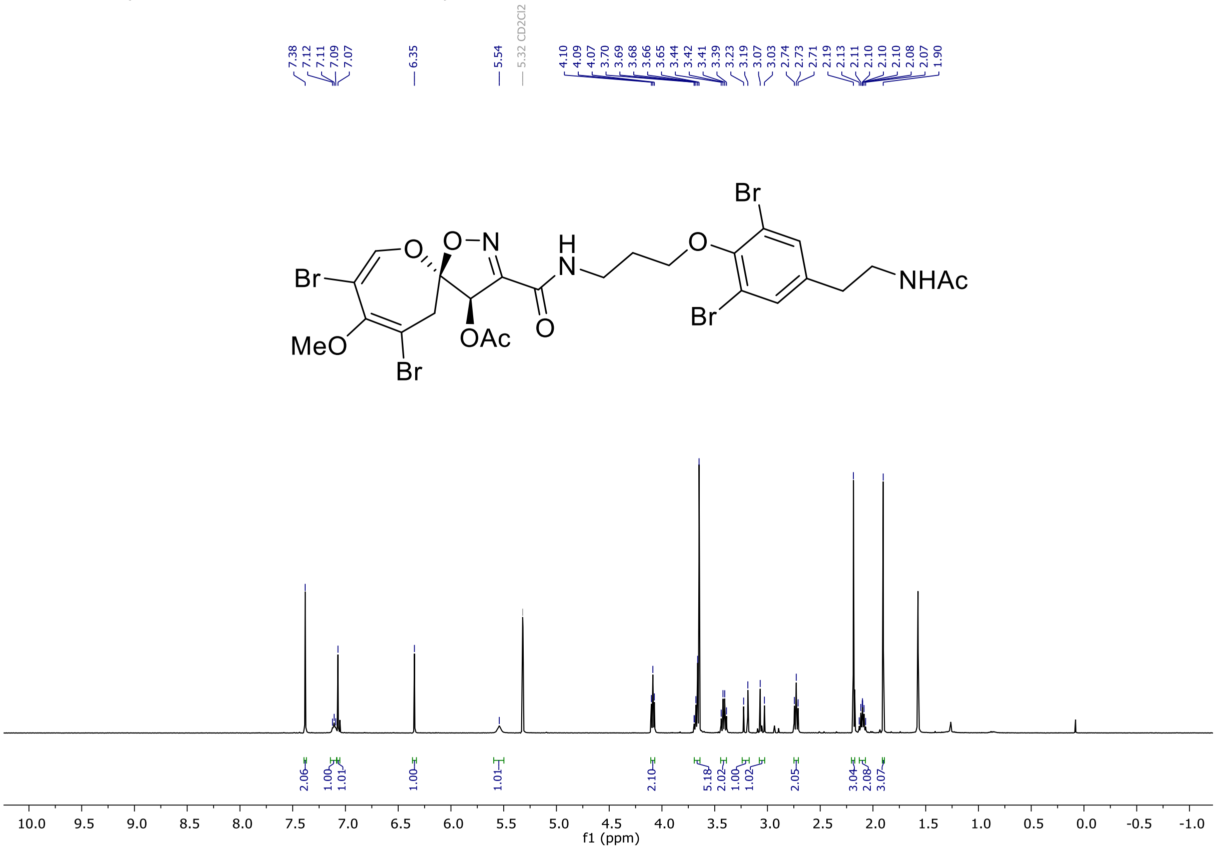

^{13}C NMR (101 MHz, CD_2Cl_2, 25 °C) of acetamide **S21**:

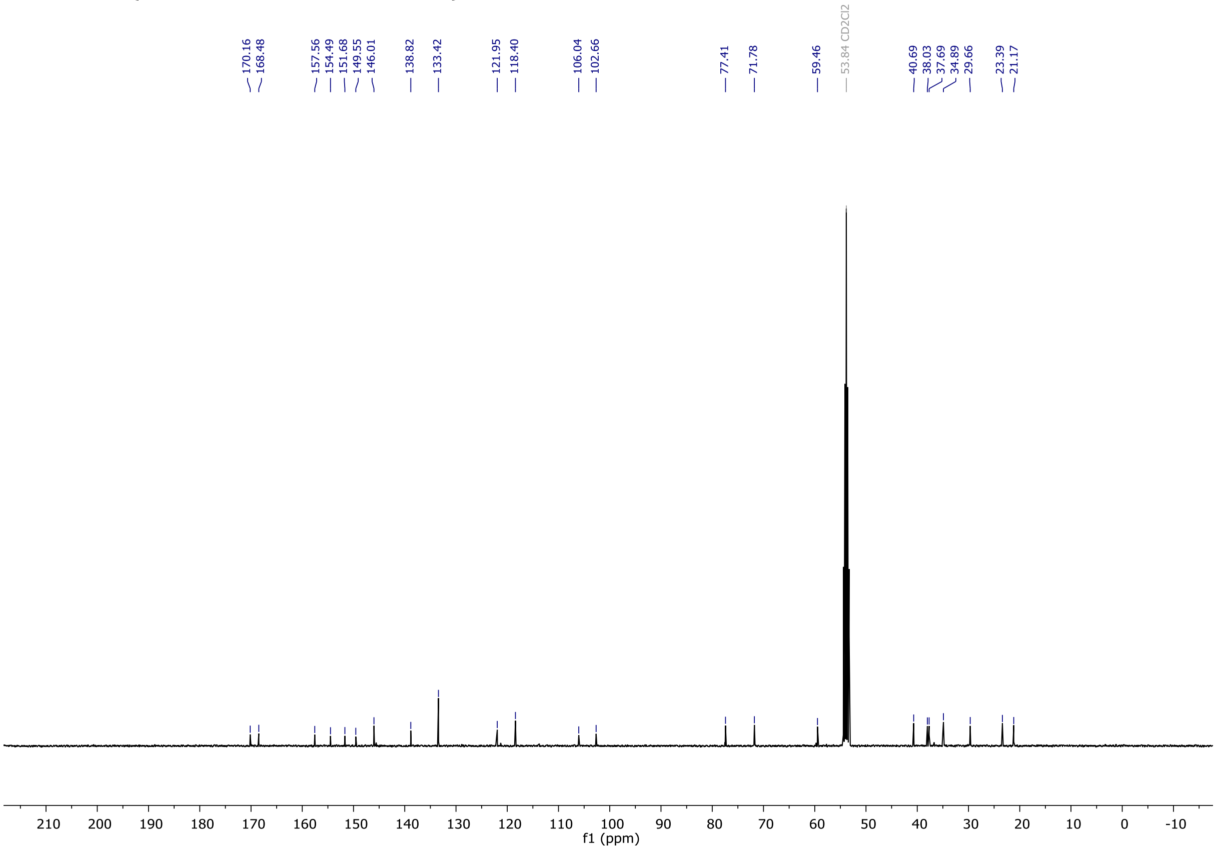

H-H COSY NMR (CD_2Cl_2, 25 °C) of acetamide **S21**:

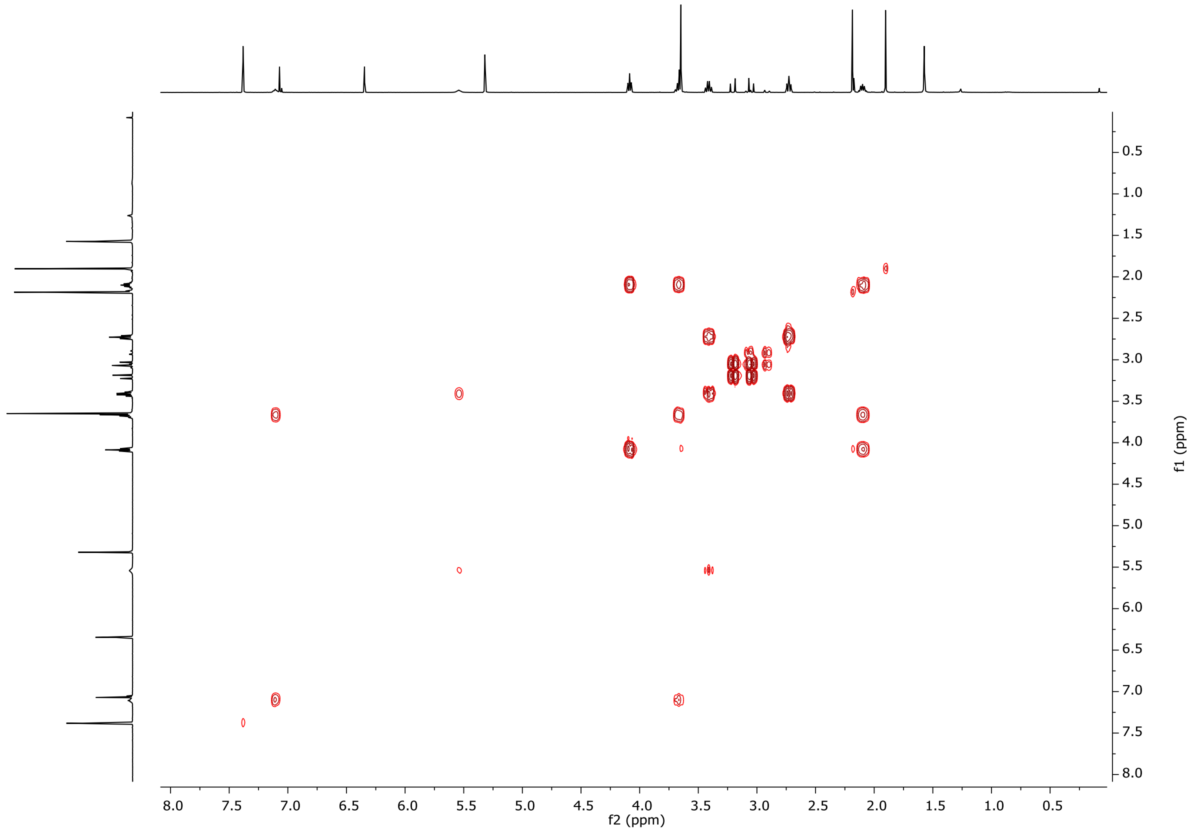

C-H HSQC NMR (CD_2Cl_2, 25 °C) of acetamide **S21**:

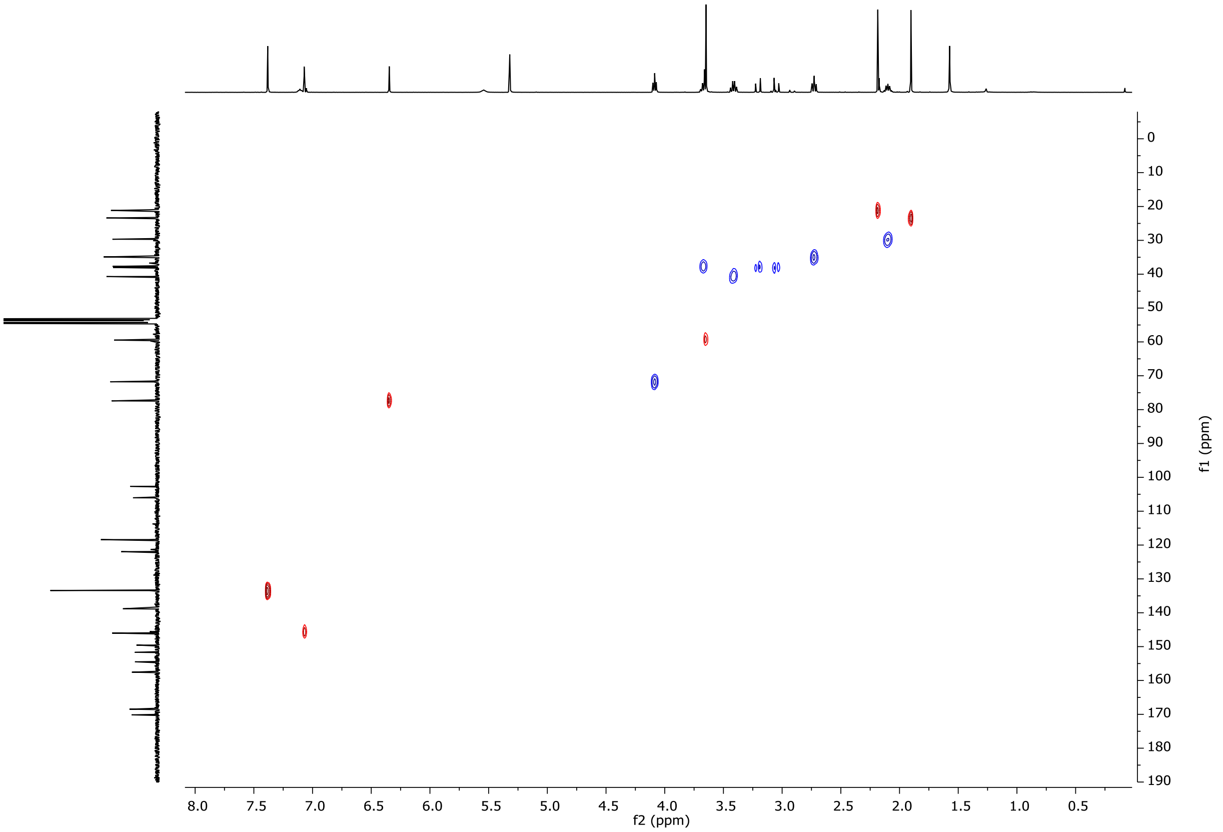

C-H HMBC NMR (CD_2Cl_2, 25 °C) of acetamide **S21**:

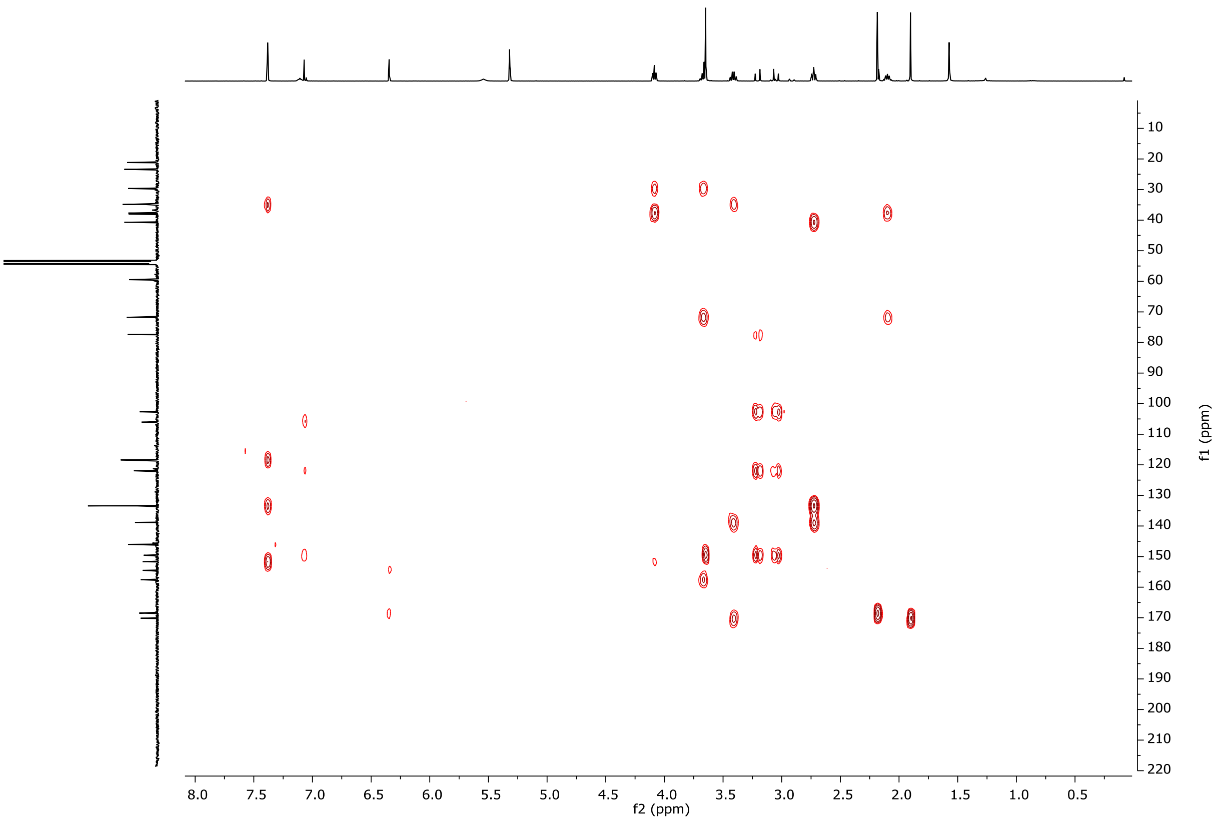

Crystallographic Data

Crystal data and structure refinement for aldehyde 7

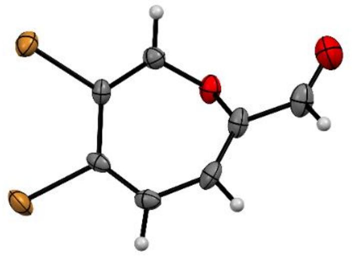

The thermal ellipsoids are shown at 50% probability level.

Empirical formula	$C_7H_4Br_2O_2$	
Formula weight	279.92	
Temperature	183(2) K	
Wavelength	0.71073 Å	
Crystal system	Orthorhombic	
Space group	Pbca (no. 61)	
Unit cell dimensions	a = 7.3783(6) Å	α = 90°.
	b = 7.4998(6) Å	β = 90°.
	c = 29.400(2) Å	γ = 90°.
Volume	1626.9(2) $Å^3$	
Z	8	
Density (calculated)	2.286 Mg/m^3	
Absorption coefficient	9.908 mm^{-1}	
F(000)	1056	
Crystal size	0.100 x 0.020 x 0.020 mm^3	
Theta range for data collection	2.771 to 24.496°.	
Index ranges	-8<=h<=8, -8<=k<=8, -34<=l<=34	
Reflections collected	20220	
Independent reflections	1363 [R(int) = 0.1110]	
Completeness to theta = 24.496°	99.9 %	
Absorption correction	Semi-empirical from equivalents	
Max. and min. transmission	0.801 and 0.599	
Refinement method	Full-matrix least-squares on F^2	
Data / restraints / parameters	1363 / 3 / 112	
Goodness-of-fit on F^2	1.078	
Final R indices [I>2sigma(I)]	R_1 = 0.0381, wR_2 = 0.0748	
R indices (all data)	R_1 = 0.0571, wR_2 = 0.0801	
Extinction coefficient	n/a	
Largest diff. peak and hole	1.193 and -0.599 e.$Å^{-3}$	

Crystal data and structure refinement for arene oxide 9

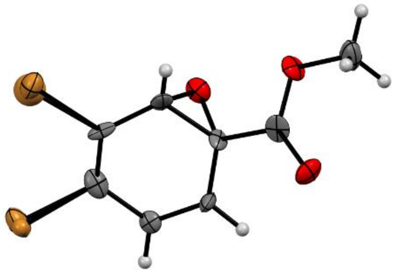

The thermal elipsoids are shown at 50% probability level.

Empirical formula	$C_8H_6Br_2O_3$	
Formula weight	309.95	
Temperature	183.00 K	
Wavelength	0.71073 Å	
Crystal system	Orthorhombic	
Space group	$Pna2_1$ (no. 33)	
Unit cell dimensions	a = 34.734(3) Å	α = 90°.
	b = 4.1471(4) Å	β = 90°.
	c = 6.4181(6) Å	γ = 90°.
Volume	924.50(15) $Å^3$	
Z	4	
Density (calculated)	2.227 Mg/m^3	
Absorption coefficient	8.737 mm^{-1}	
F(000)	592	
Crystal size	0.18 x 0.08 x 0.01 mm^3	
Theta range for data collection	2.345 to 24.972°.	
Index ranges	-41<=h<=41, -4<=k<=4, -7<=l<=7	
Reflections collected	12960	
Independent reflections	1612 [R(int) = 0.0564]	
Completeness to theta = 24.972°	99.9 %	
Absorption correction	Semi-empirical from equivalents	
Max. and min. transmission	0.7452 and 0.5665	
Refinement method	Full-matrix least-squares on F^2	
Data / restraints / parameters	1612 / 1 / 128	
Goodness-of-fit on F^2	1.317	
Final R indices [I>2sigma(I)]	R_1 = 0.0760, wR_2 = 0.1844	
R indices (all data)	R_1 = 0.0784, wR_2 = 0.1855	
Absolute structure parameter	0.06(9)	
Extinction coefficient	n/a	
Largest diff. peak and hole	1.965 and -1.455 e.$Å^{-3}$	

Crystal data and structure refinement for ester 16a

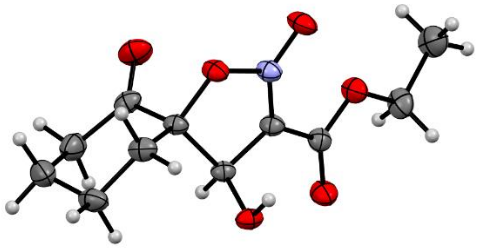

The thermal ellipsoids are shown at 50% probability level.

Empirical formula	$C_{11}H_{15}NO_6$	
Formula weight	257.24	
Temperature	183.00 K	
Wavelength	0.71073 Å	
Crystal system	Triclinic	
Space group	P-1 (no. 2)	
Unit cell dimensions	a = 9.2424(8) Å	α = 70.982(4)°.
	b = 11.2166(13) Å	β = 75.461(4)°.
	c = 12.8691(16) Å	γ = 76.005(3)°.
Volume	1202.1(2) Å^3	
Z	4	
Density (calculated)	1.421 Mg/m^3	
Absorption coefficient	0.117 mm^{-1}	
F(000)	544	
Crystal size	0.39 x 0.18 x 0.12 mm^3	
Theta range for data collection	1.950 to 27.138°.	
Index ranges	-11<=h<=11, -14<=k<=14, -16<=l<=16	
Reflections collected	39859	
Independent reflections	5310 [R(int) = 0.0553]	
Completeness to theta = 25.242°	99.9 %	
Absorption correction	Semi-empirical from equivalents	
Max. and min. transmission	0.9705 and 0.9277	
Refinement method	Full-matrix least-squares on F^2	
Data / restraints / parameters	5310 / 2 / 336	
Goodness-of-fit on F^2	1.097	
Final R indices [I>2sigma(I)]	R_1 = 0.0377, wR_2 = 0.0982	
R indices (all data)	R_1 = 0.0458, wR_2 = 0.1009	
Extinction coefficient	0.022(2)	
Largest diff. peak and hole	0.439 and -0.342 e.Å^{-3}	

References

1) Yi, C.-B.; She, Z.-Y.; Cheng, Y.-F.; Qu, J. Redox-Neutral α-C–H Functionalization of Pyrrolidin-3-ol. *Org. Lett.* **2018**, *20*, 668–671.

2) Yanai, H.; Sasaki, Y.; Yamamoto, Y.; Matsumoto, T. Chemoselective Two-Directional Reaction of Bifunctionalized Substrates: Formal Ketal-Selective Mukaiyama Aldol Type Reaction. *Synlett* **2015**, *26*, 2457–2461.

3) Wang, C.; Chen, Y.-H.; Wu, H.-C.; Wang, C.; Liu, Y.-K. The Quinary Catalyst–Substrate Complex Induced Construction of Spiro-Bridged or Cagelike Polyheterocyclic Compounds via a Substrate-Controlled Cascade Process. *Org. Lett.* **2019**, *21*, 6750–6755.

4) Millet, A.; Dailler, D.; Larini, P.; Baudoin, O. Ligand-Controlled α- and β-Arylation of Acyclic N-Boc Amines. *Angew. Chem. Int. Ed.* **2014**, *53*, 2678–2682.

5) Machida, S.; Usuba, K.; Blaskovich, M. A.; Yano, A.; Harada, K.; Sebti, S. M.; Kato, N.; Ohkanda, J. Module Assembly for Protein-Surface Recognition: Geranylgeranyltransferase I Bivalent Inhibitor for Simultaneous Targeting of Interior and Exterior Protein Surfaces. *Chem. Eur. J.* **2008**, *14*, 1392–1401.

6) Pániková, T.; Mitrová, K.; Halamová, T.; Mrzílková, K.; Pícha, J.; Chrudinová, M.; Kurochka, A.; Selicharová, I.; Žáková, L.; Jiráček, J. Insulin Analogues with Altered Insulin Receptor Isoform Binding Specifities and Enhanced Aggregation Stabilities. *J. Med. Chem.* **2021**, *64*, 14848–14859.

7) Roll, D. M.; Chang, C. W. J.; Scheuer, P. J.; Gray, G. A.; Shoolery, J. N.; Matsumoro, G. K.; Van Duyne, G. D.; Clardy, J. Structure of the Psammaplysins. *J. Am. Chem. Soc.* **1985**, *107*, 2916–2920.
